# Manganese-Catalyzed Dehydrogenative Silylation of Alkenes Following Two Parallel Inner-Sphere Pathways

**DOI:** 10.1021/jacs.1c09175

**Published:** 2021-10-13

**Authors:** Stefan Weber, Manuel Glavic, Berthold Stöger, Ernst Pittenauer, Maren Podewitz, Luis F. Veiros, Karl Kirchner

**Affiliations:** † Institute of Applied Synthetic Chemistry, Vienna University of Technology, Getreidemarkt 9, A-1060 Vienna, Austria; ‡ X-Ray Center, Vienna University of Technology, Getreidemarkt 9, A-1060 Vienna, Austria; § Institute of Chemical Technologies and Analytics, Vienna University of Technology, Getreidemarkt 9, A-1060 Vienna, Austria; ∥ Institute of Materials Chemistry, Vienna University of Technology, Getreidemarkt 9, A-1060 Vienna, Austria; ⊥ Centro de Química Estrutural, Instituto Superior Técnico, Universidade de Lisboa, Av. Rovisco Pais No. 1, 1049-001 Lisboa, Portugal

## Abstract

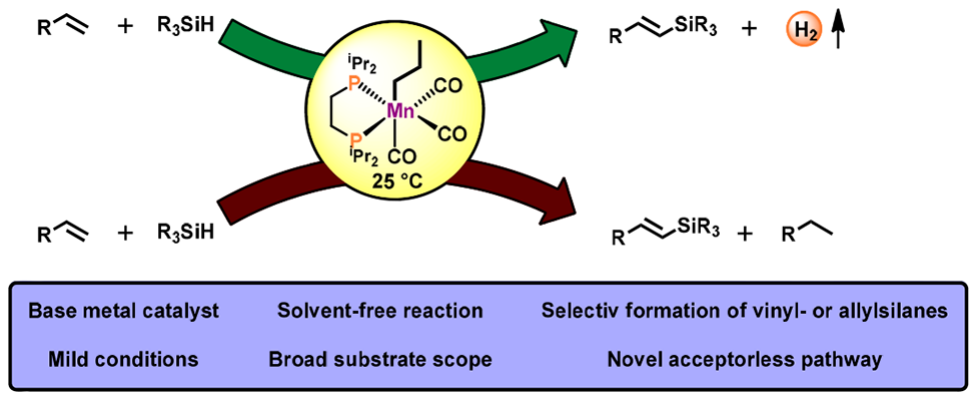

We report on an additive-free Mn(I)-catalyzed dehydrogenative silylation of terminal alkenes. The most active precatalyst is the bench-stable alkyl bisphosphine Mn(I) complex *fac-*[Mn(dippe)(CO)_3_(CH_2_CH_2_CH_3_)]. The catalytic process is initiated by migratory insertion of a CO ligand into the Mn–alkyl bond to yield an acyl intermediate which undergoes rapid Si–H bond cleavage of the silane HSiR_3_ forming the active 16e^–^ Mn(I) silyl catalyst [Mn(dippe)(CO)_2_(SiR_3_)] together with liberated butanal. A broad variety of aromatic and aliphatic alkenes was efficiently and selectively converted into *E*-vinylsilanes and allylsilanes, respectively, at room temperature. Mechanistic insights are provided based on experimental data and DFT calculations revealing that two parallel reaction pathways are operative: an acceptorless reaction pathway involving dihydrogen release and a pathway requiring an alkene as sacrificial hydrogen acceptor.

## Introduction

Organosilane-based compounds are widely employed in a broad variety of commercial products such as coating materials, paints, or medicinal applicants, which is attributed to high chemical and thermal stability as well as to their low toxicity.^1^ Furthermore, they may serve as powerful platform compounds in organic synthesis.^2^ Vinyl- and allylsilanes display important substance classes for the synthesis of small molecules and as building blocks in macromolecular chemistry.^3^ Unsaturated silanes may serve as substrates in the synthesis of carbonyl moieties and allylic alcohols^4^ or as nontoxic reagents in cross-coupling reactions.^5^

**Scheme 1:**
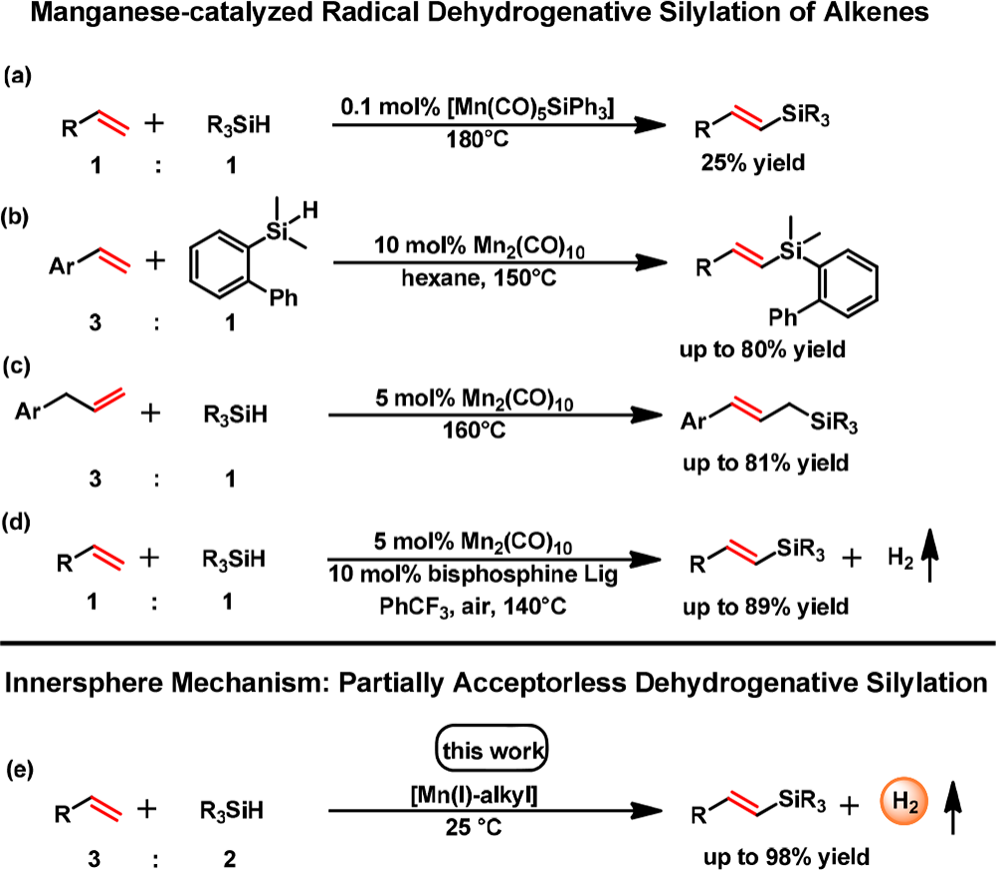
Manganese-Catalyzed DS of Alkenes

Hydrosilylation, catalyzed by transition metals, displays the most common reaction pathway for the synthesis of organosilanes whereas unsaturated silanes may be synthesized by hydrosilylation of alkynes.^6,7^ However, selectivity problems such as α- or β-addition to the triple bond or undesired addition of silane to the formed vinyl- or allylsilane often arise. Dehydrogenative silylation (DS) of alkenes displays an interesting alternative to that. Noble metals such as Rh,^8^ Ir,^9^ or Ru^10^ are commonly used in DS reactions. Nevertheless, base metals such as Fe^11^ or Co^12^ were also employed within the past decade. Although being an emerging field, Manganese-based DS reactions^13^ suffer from high catalyst loadings and harsh reaction conditions as depicted in Scheme 1. A drawback of DS in general is attributed to the fact that an excess of alkene or the addition of a sacrificial hydrogen acceptor (SHA) is required to scavenge the formed metal-hydride intermediate due to β-hydride elimination in the product releasing step.

The development of acceptorless dehydrogenative silylation (ADS) protocols releasing hydrogen gas as sole byproduct would be beneficial to increase the atom efficiency. So far, examples of ADS are exceedingly rare. Xu and co-workers reported on an elegant photoredox induced hydrogen-atom transfer (HAT) cascade in combination with cobalt catalysis for the ADS of alkenes yielding allylsilanes in high selectivity. However, 2 equiv of silane, 5 mol % of the catalyst, 20 mol % of HAT-catalyst, and the addition of a photocatalyst and pyridine as base were required for this procedure.^12b^ Very recently Xie and co-workers reported on a manganese-based ADS based on an HAT mechanism. A high atom efficiency could be achieved requiring, however, harsh reaction conditions (140 °C), high catalyst loading of [Mn_2_(CO)_10_] (5 mol %), and the use of the fluorinated solvent trifluoromethylbenzene.^13d^

**Scheme 2:**
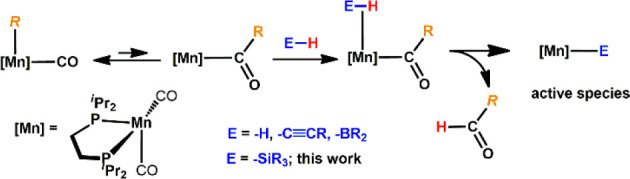
Formation of the Active Species *via* Migratory Insertion and Deprotonation of the Entering Ligand

We recently described the application of well-defined Mn(I)–alkyl complexes in the hydrogenation of nitriles,^14^ ketones,^15^ CO_2_,^16^ and alkenes.^17^ We took advantage of the fact that Mn(I)–alkyl carbonyl complexes undergo migratory insertion of the nucleophilic alkyl ligand into the polarized CO moiety, yielding a coordinatively unsaturated acyl complex, which may activate weakly polar E–H bonds (e.g., E = −H, −C≡C–R, −SiR_3_) (Scheme 2).^18^ We also demonstrated that Mn(I)–alkyl complexes are capable of activating C–H bonds of terminal alkynes converting aromatic and aliphatic terminal alkynes efficiently and selectively into head-to-head *Z*-1,3-enynes and head-to-tail gem-1,3-enynes.^19^ Most recently, we showed that Mn(I)–alkyl complexes also catalyze the hydroboration of terminal alkenes (involving B–H bond activation) and the 1,2-diboration of terminal alkynes with pinacolborane (involving C–H bond activation).^20^ Encouraged by these findings, we wondered if Si–H bonds may also be activated thereby initiating hydrosilylations and/or DS reactions of alkenes.

Here, we describe the activity of *fac*-[Mn(dippe)(CO)_3_(CH_2_CH_2_CH_3_)] (dippe = 1,2-bis(di-isopropylphosphino) (**1**) and *fac-*[Mn(dpre)(CO)_3_(CH_2_CH_2_CH_3_)] (dpre = 1,2-bis(di-*n*-propylphosphino) (**2**) as precatalysts for the DS of alkenes to afford selectively *E*-vinylsilanes. This is a rare example of a base-metal catalyzed DS of alkenes which proceeds at room temperature following two parallel catalytic cycles: an acceptorless reaction pathway involving dihydrogen release and a pathway requiring an alkene as a sacrificial hydrogen acceptor.

## Results and Discussion

The catalytic performance of alkyl complexes **1** and **2** for the DS of 4-chlorostyrene with HSiEt_3_ as model substrates was first investigated. Selected optimization experiments are depicted in Table 1. Gratifyingly, high selectivity toward the *E-*isomer could be achieved while the formation of hydrosilylated alkane was not observed. High reactivity at room temperature under solvent-free conditions was observed for complex **1**. Complex **2** turned out to be less active. Interestingly, the ratio of silane to alkene could be reduced to 1:1.5, which is uncommon in the field of DS reaction (Table 1, entry 4).

**Table 1:**
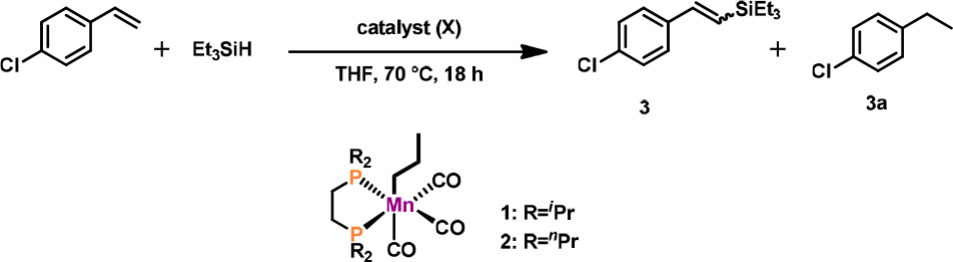
Optimization Reactions for DS of 4-Chlorostyrene with HSiEt_3_^a^

entry	[Mn] (mol %)	conversion (%)	*E*/*Z*	**3**:**3a**
1	**1** (3)	>99	97:3	2:1
2	**2** (3)	89	94:6	2.1:1
3^b^	**1** (2)	>99	>99:1	2:1
4^b^^,^^c^	**1** (2)	>99	>99:1	2:1
5^b^^,^^c^^,^^d^	**1** (2)	39^d^	>99:1	9:1
6^b^^,^^e^	**1** (2)	>99^e^	>99:1	2.2:1
7^b^^,^^f^	**1** (2)	>99	>99:1	2:1

a Reaction conditions: HSiEt_3_ (0.56 mmol, 1 equiv), 4-chlorostyrene (1.12 mmol, 2 equiv), and 0.5 mL of anhydrous THF, 70 °C, 18 h. Conversion of silane, *E*/*Z*-ratio of **3**:**3a** determined by GC/MS.

b Neat, 25 °C, 24 h.

c 4-Chlorostyrene (0.84 mmol, 1.5 equiv).

d 3 equiv of 3,3-dimethylbutene used as SHA, conversion to **3** reported.

e 3 equiv of HSiEt_3_, conversion of alkene reported.

f 5 equiv of 4-chlorostyrene

Typically, 2 (or more) equiv of alkene are employed to quench the *in situ* generated hydride species. An alternative approach in the literature is the utilization of a sacrificial hydrogen acceptor (SHA) such as 3,3-diemethylbuten or cyclooctene.^12a^ By using 3 equiv of 3,3-dimethylbutene as SHA, the ratio of **3**:**3a** was drastically increased to 9:1, but led to a conversion of only 39% to afford **3**, while full conversion of silane was detected. This is attributed to an undesired DS of the SHA (Table 1, entry 5). Increasing the ratio of HSiEt_3_ to alkene to 3:1 or increasing the ratio of alkene to silane 5:1 only led to negligible changes in the product distribution (Table, 1 entries 6 and 7).

Having established the optimized reaction conditions, scope, and limitation of the introduced system was investigated. In order to ensure a high conversion of silane, in the following 1.8 equiv of alkene was used. A broad variety of different aromatic substrates could be efficiently converted to the desired DS products with excellent selectivity toward the *E*-isomer. Styrene derivatives with electron-withdrawing groups (Table 2, **3** and **6**) or electron-donating groups (Table 2, **12**, **15**, and **18**) gave excellent yields. Functional groups such as halides, ethers, or amines were well-tolerated. However, the pyridine-based substrate was not converted indicating an undesirable interaction with the catalyst such as coordination of the pyridine moiety blocking a vacant side during reaction, thus, indicating a different reaction mechanism as described by Xie and co-workers for a radical based reaction, tolerating pyridines as substrates.^13d^ Moreover, 1,1- or 1,2-disubstitured alkenes or phenylacetylene did not show any reactivity in the investigated transformation.

**Table 2:**
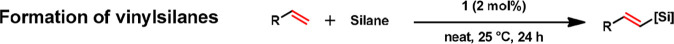

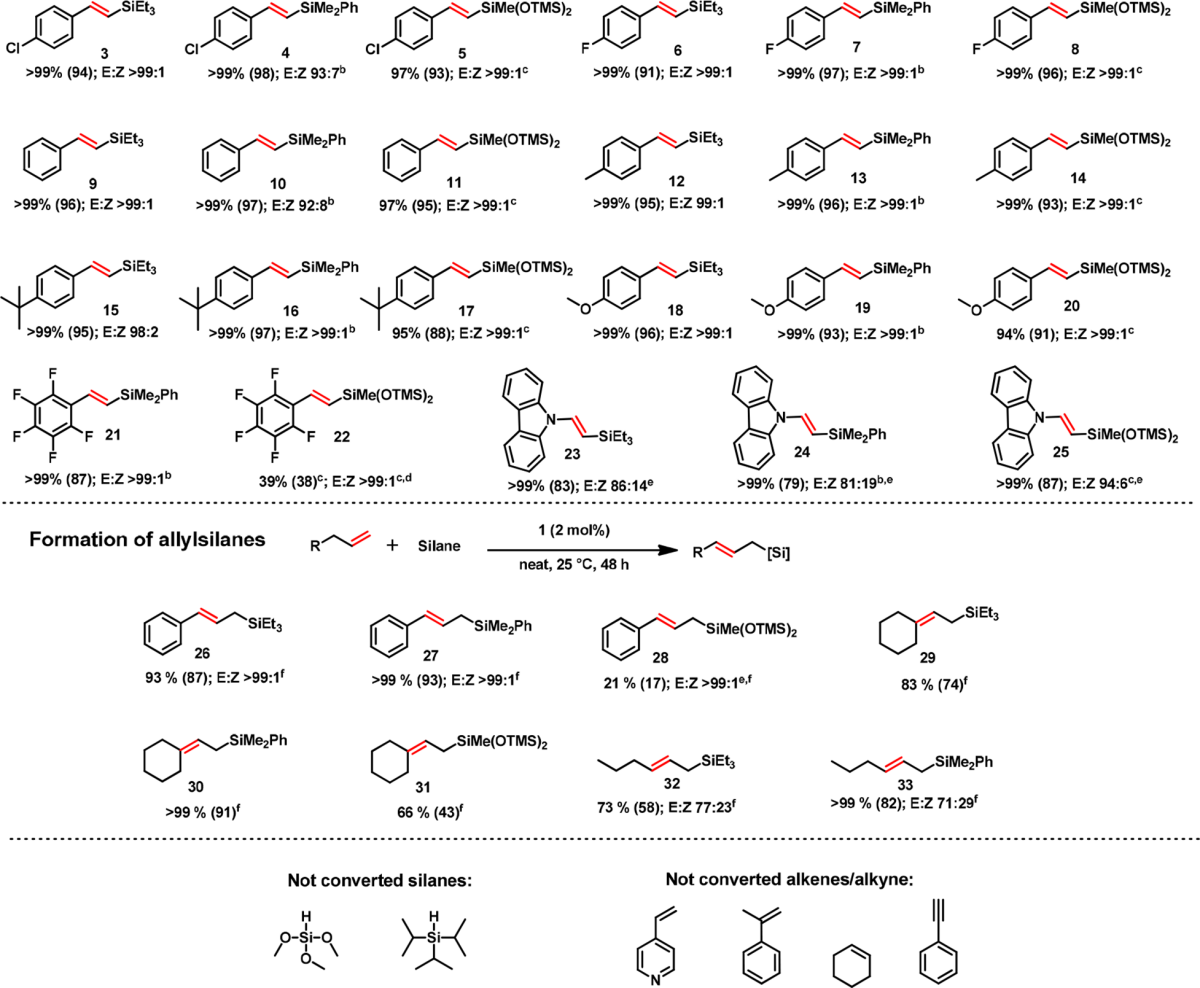
Substrate Scope of DS for Terminal Alkenes Catalyzed by **1**^a^

a Reaction conditions: silane (0.56 mmol, 1 equiv), alkene (1.01 mmol, 1.8 equiv), **1** (2 mol %), neat, 24 h, conversion of silane and *E*/*Z* ratio determined by GC/MS, isolated yield given in parentheses.

b 0.5 mol %.

c 48 h.

d Yield determined by ^1^H NMR analysis using 0.5 equiv of 1,4-dioxane as standard.

e 0.1 mL of THF as solvent.

f 48 h.

Exploring the reaction scope regarding different silanes revealed high reactivity for HSiMe_2_Ph whereas the catalyst loading could be decreased to 0.5 mol %. Thus, excellent yield could be achieved, for pentafluoro styrene as substrate (Table 2, **21**). Furthermore, excellent yields could be achieved, employing the commercially relevant 1,1,1,3,5,5,5-heptamethyltrisiloxane, although the reaction time had to be increased to achieve high conversions. Slightly lower reactivity toward styrenes, bearing an electron-donating group, could be detected in the case of trisiloxane (Table 2, **14**, **17**, and **20**). In all of the above-mentioned cases, the *E*/*Z* ratio was 92:8 or higher, whereas a moderate decrease in selectivity could be detected, when vinylcarbazol was used as substrate (Table 2, **23**–**25**).

In general, the highest selectivity could be achieved when employing the sterically demanding trisiloxane. Investigation of other tertiary silanes such as trimethoxy- or triisopropylsilane gave no conversion. Presumably, trimethoxysilane is sterically not demanding enough for this transformation, whereas, on the other hand, triisopropyl silane seems to be sterically too hindered.

Investigation of the substrate scope of aliphatic alkenes resulted in an unexpected reaction pattern. In all investigated cases, exclusively allylsilanes instead of vinylsilanes were obtained. This may be attributed to γ-hydride elimination rather than a β-hydride elimination.

Very high selectivity toward *E*-alkenes could be observed in the case of allylbenzene (Table 2, **26**–**28**). The usage of vinylcyclohexane resulted in the formation of a trisubstitued alkene (Table 2, **29**–**31**).

Moderate *E*-selectivity could be detected for 1-hexene as substrate. It should be noted that no hydrosilylated product could be detected upon substrate scope investigation of aromatic and aliphatic alkenes.

The homogeneity of the reaction was confirmed by addition of one drop of mercury where no decrease of reactivity and selectivity was observed for the DS of 4-chlorostyrene and HSiMe_2_Ph. In the presence of 1 equiv of PMe_3_ (with respect to substrate), only traces of product formation could be detected, which indicates an inner-sphere mechanism, due to coordination of PMe_3_ at a vacant side of the active species. The presence of 1 equiv of 2,2,6,6-tetramethylpiperidinyloxyl (TEMPO) did not influence the catalytic reaction, thus, ruling out a radical reaction pathway. This seems to be unique in the field of manganese catalyzed DS reactions of alkenes, since all literature known examples as shown in Scheme 1 appear to proceed *via* radical routes.^13^

**Scheme 3:**
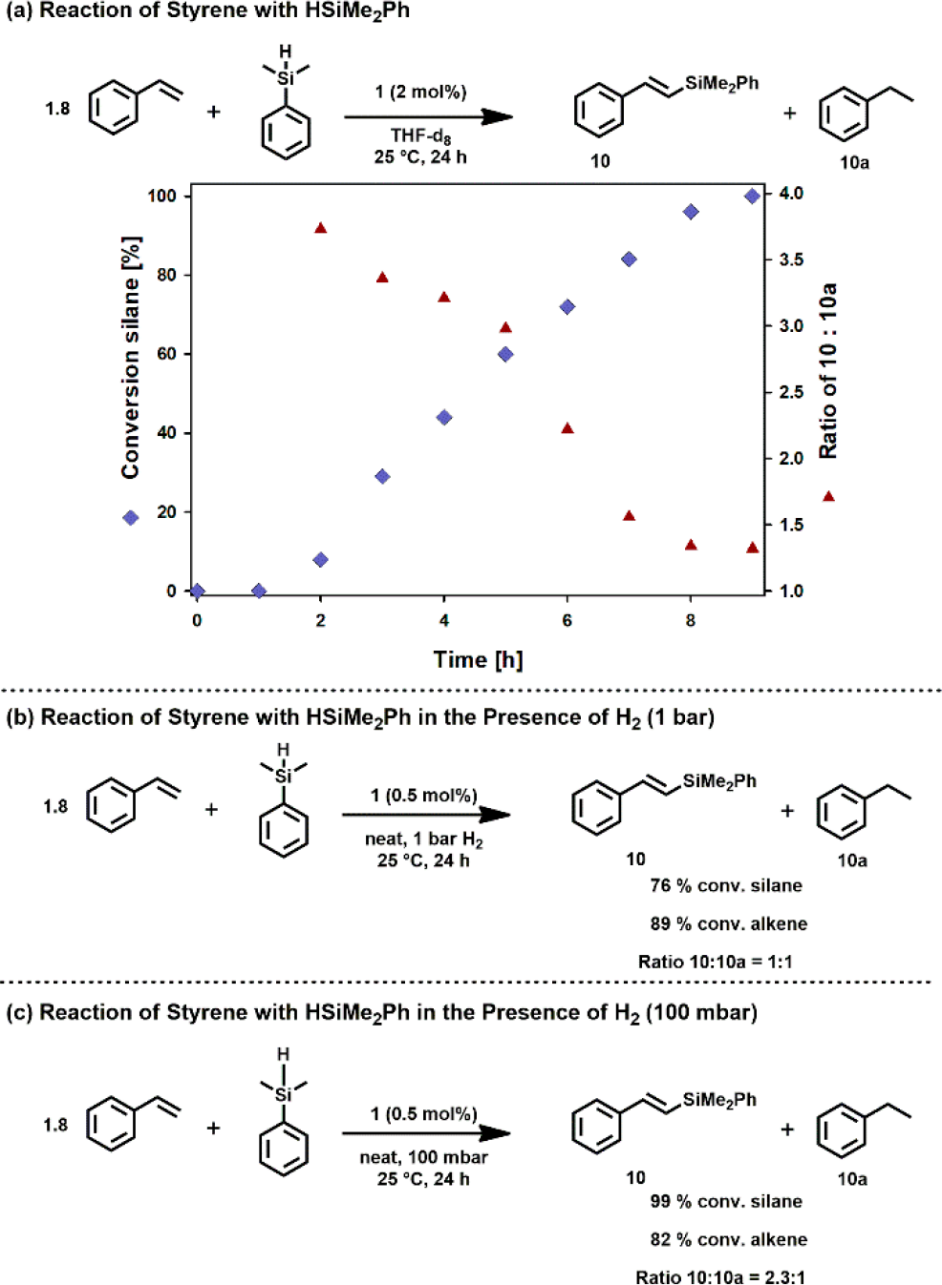
Kinetic Profile and Mechanistic Experiments

A kinetic profile of the reaction HSiMe_2_Ph and styrene is depicted in Scheme 3. After an offset period, the reaction proceeds in an almost linear fashion (Scheme 3a, blue squares). It should be noted that the reactivity of the system is lower by a factor of about 2–3 in THF as solvent when compared to the neat reaction. At low conversion of silane (and alkene respectively) a ratio of **10**:**10a** of 3.7:1 could be detected (Scheme 3a, red triangles). This indicates that in the initial stage of the reaction an acceptorless DS is the dominating reaction pathway. In fact, hydrogen gas could be detected in a headspace analysis of the reaction mixture, which clearly proves that an acceptorless pathway is involved in the catalytic reaction. As the reaction proceeds, the ratio of DS product to alkane decreases.

A similar behavior was observed for 4-fluorostyrene, 4-methylstyrene, or 4-methoxystyrene. The ratio of **10**:**10a** is dependent on the concentration of hydrogen in the system. Accordingly, when the reaction was carried out under 1 bar of hydrogen atmosphere an acceptorless DS no longer took place (Scheme 3b) and the products were formed in an almost 1:1 ratio. If the reaction was carried out under reduced pressure, a ratio of 2.3:1 of **10**:**10a** was observed (Scheme 3c).

**Scheme 4:**
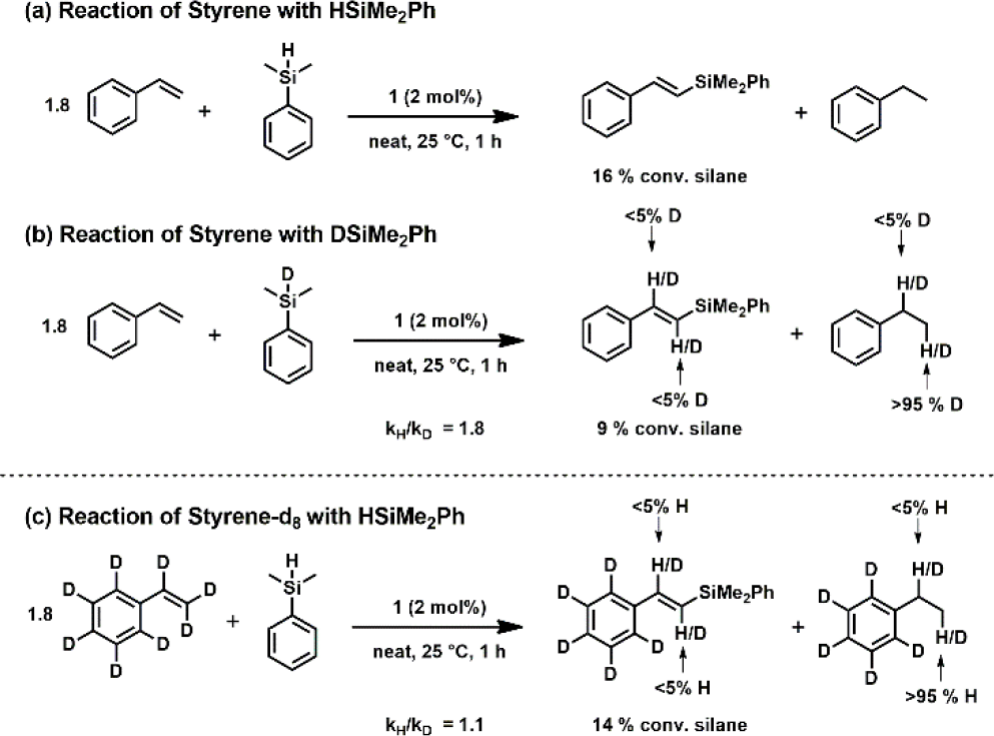
Determination of KIE and Deuterium Incorporation

To gain insight in the rate-determining step of the catalysis, kinetic isotope experiments were carried out. In two separate experiments, a kinetic isotope effect (KIE) of 1.8 for nondeuterated *vs* deuterated silane could be detected (Schemes 4a and 4b). This value is slightly lower than that for a cobalt-catalyzed DS as reported by Chirik and co-workers.^12a^ In an additional experiment, fully deuterated styrene was used as substrate where a KIE of merely 1.1 was detected. The rate-determining step seems to be the cleavage of the Si–H bond during the activation of the catalyst rather than hydride elimination upon product release. This is also supported by the offset in the kinetic profile depicted in Scheme 3a.

The incorporation of deuterium (or hydrogen) in products upon full conversion was studied with DSiMe_2_Ph. Selective incorporation of deuterium in the terminal position of ethylbenzene was observed, whereas negligible deuterium content was found in the DS product or the benzylic position of ethylbenzene (Scheme 4b). If styrene-*d*_8_ was used as substrate in combination with HSiMe_2_Ph, only traces of hydrogen were found in the DS product and a high level of hydrogen incorporation on the terminal carbon of ethylbenzene was observed (Scheme 4c).

**Scheme 5:**
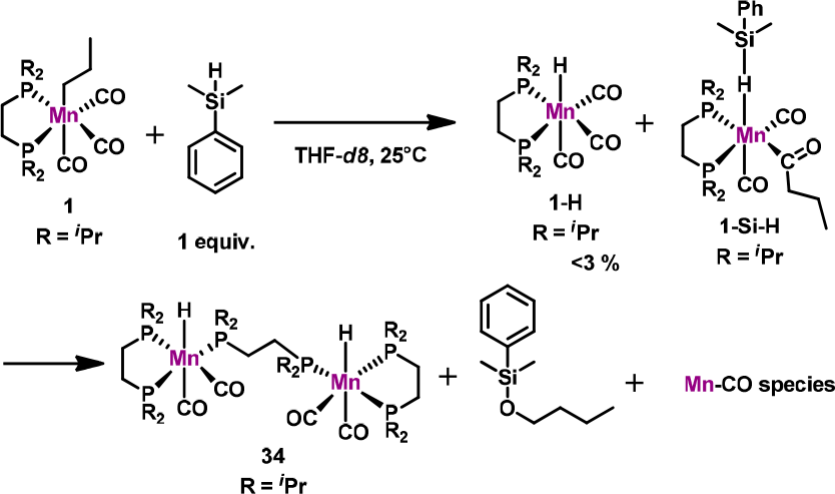
Stochiometric Reaction of **1** with HSiPhMe_2_

To gain further insight in the reaction mechanism, stochiometric reactions of **1** with silanes were carried out. If **1** was treated with an equimolar amount of HSiMe_2_Ph in THF-*d*_8_, a small amount (<3%) of the known tricarbonyl hydride complex [Mn(dippe)(CO)_3_H] was generated (Scheme 5).^21^ This complex did not show any catalytic activity in the DS reaction of alkenes. Furthermore, a new compound could be detected *via*^1^H and ^31^P{^1^H} NMR spectroscopy. This species gave rise to a doublet of doublet centered at −10.17 ppm in the ^1^H NMR spectrum (DFT calculated value is −9.6 ppm). The chemical shift is in the same region as described by Schubert and co-workers for the manganese complex [Mn(CpMe)(CO_2_)(H-SiR_2_SiR_2_H)] complex featuring an agostic Si–H bond.^22^ The ^31^P{^1^H} NMR spectrum exhibits two doublets at 121.3 and 108.7 ppm. ^1^H/^31^P 2D-NMR analysis revealed that both signals belong to a single species (for details see SI).

It has to be noted that protonation of the alkyl ligand of **1** by the silane, which would lead to liberation of propane and generation of a silyl complex, was not observed. In fact, such a reaction was described by Chirik and co-workers in the case of a cobalt-catalyzed DS of alkenes.^12a^

Since manganese(I)–alkyl complexes are known to undergo migratory insertion of the alkyl into the carbonyl ligand,^18^ we believe that the resonances of the above-described compound may be tentatively assigned to the acyl complex [Mn(dippe)(CO)_2_(η^1^-C(O)CH_2_CH_2_CH_3_)(η^1^-H-SiMe_2_Ph)] (**1-Si-H**) (Scheme 5). All attempts to isolate this complex failed due to the high reactivity of this species toward the unreacted silane.

The geometry of complex **1-Si-H** was optimized by means of DFT calculations (M06/6-311++G**//PBE0/SDD,6-31G**)^23^ (Figure S15, SI) and shows a σ-complex with the silane coordinated through the Si–H bond in an apical position *trans* to a CO ligand. This is reflected in the weakening of that bond in **1-Si-H**, compared with free silane. The Si–H distance rises from 1.50 Å in HSiMe_2_Ph to 1.54 Å in **1-Si-H**, while the corresponding Wiberg indices (WI)^24^ are 0.92 and 0.66, by the same order. Also, Mn–H and Mn–Si correspond to bonding interactions with distances of 1.80 and 3.12 Å, respectively, and Wiberg indices of 0.14 (Mn–H) and 0.07 (Mn–Si).

After approximately 50% conversion of silane, decomposition of **1-Si-H** took place affording the dimeric complex **34** which is catalytically inactive. This reaction was accompanied by hydrosilylation of the released *n*-butanal. The molecular structure of this complex was unequivocally established by X-ray crystallography and NMR spectroscopy (see SI). Upon full conversion of the silane, several intractable manganese carbonyl species were formed based on IR spectroscopy. *In situ* NMR analysis during the catalytic reaction also revealed the formation of **1-Si-H**. Traces of **34** could be detected after approximately 90% conversion.

**Figure 1:**
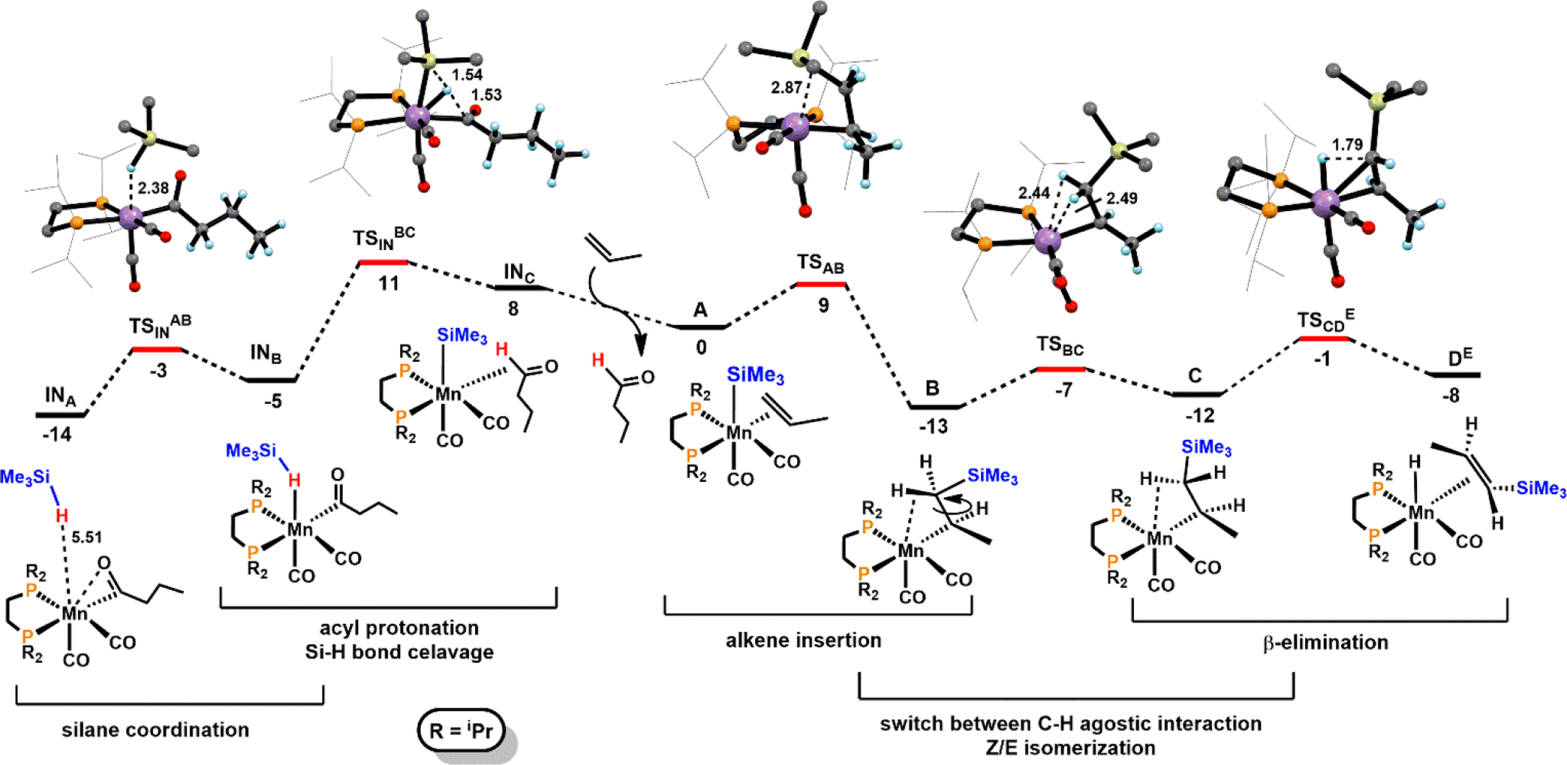
Free energy profile calculated at M06/6-311++G**//PBE0/SDD,6-31G** level for the formation of [Mn(dippe)(CO)_2_(SiMe_3_)(η^2^-CH_2_=CHCH_3_)] (**A**) and the *E*-Vinylsilene Intermediate [Mn(dippe)(CO)_2_(η^2^-CH(SiMe_3_)=CHCH_3_)] (**D**^**E**^). Free Energies (kcal/mol) are referred to **A**.

**Figure 2:**
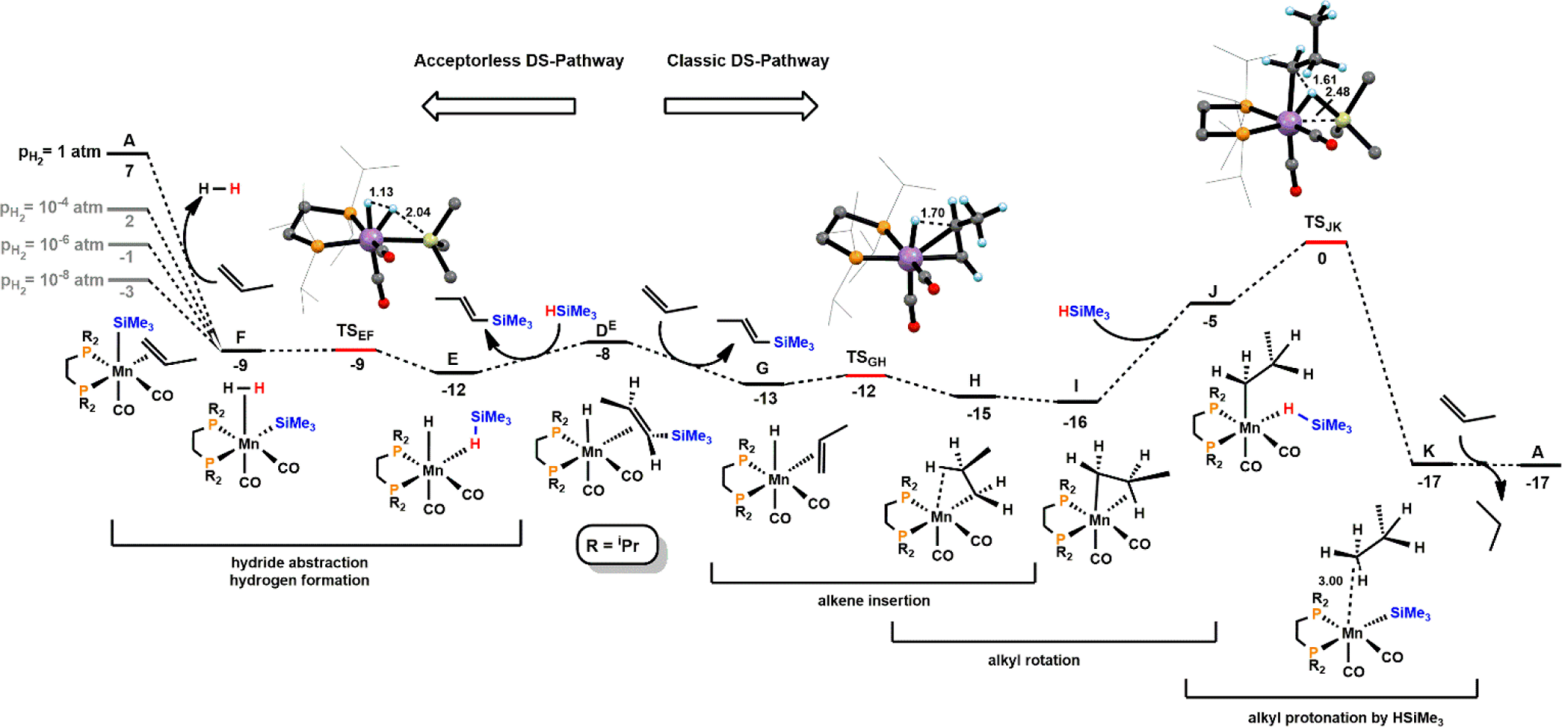
Free energy profile calculated at M06/6-311++G**//PBE0/SDD,6-31G** level for the acceptorless DS and classic DS pathways from intermediate **D**^**E**^. Free Energies (kcal/mol) are referred to **A**.

**Scheme 6:**
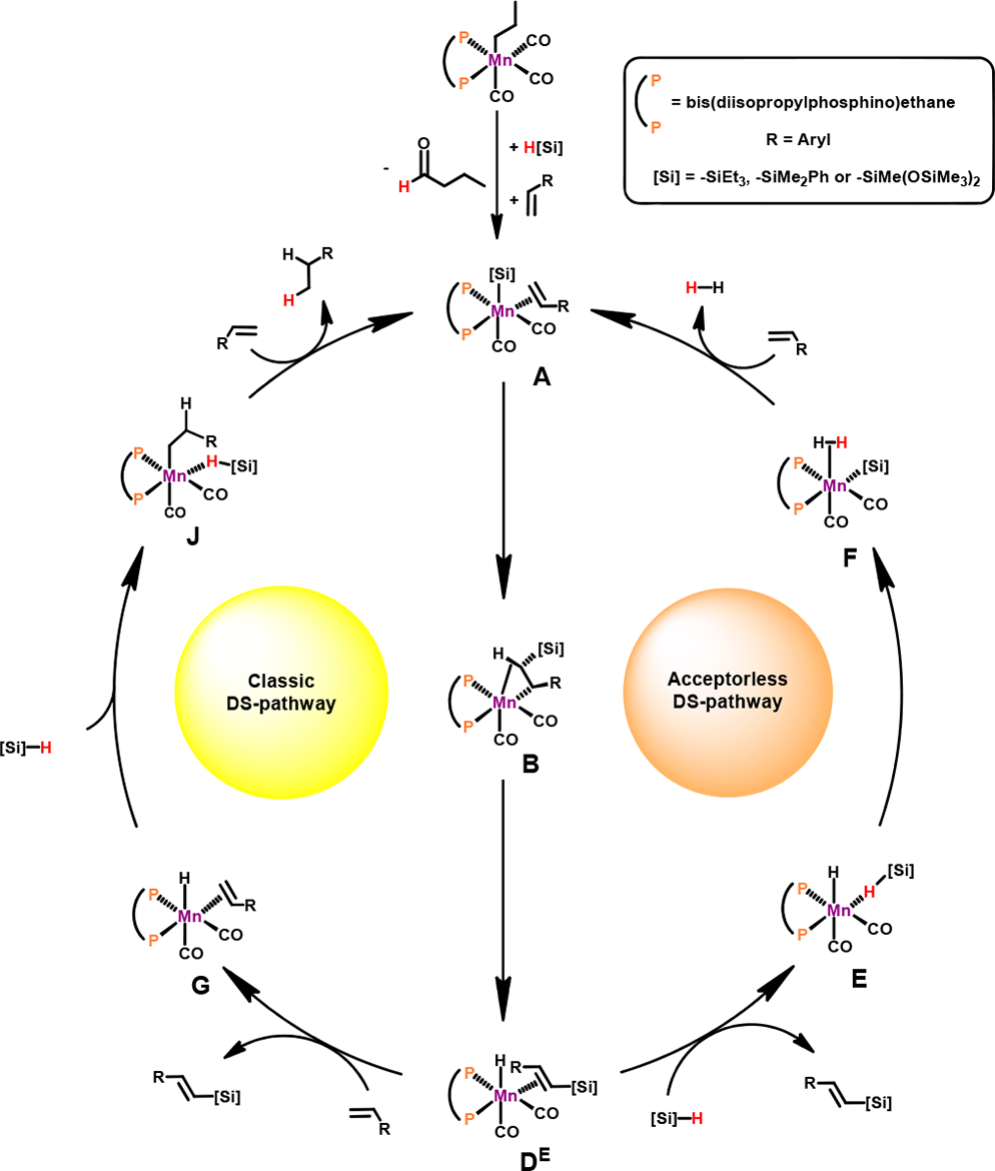
Proposed Mechanism for DS Following Two Parallel Pathways

The mechanism of the dehydrogenative silylation of terminal alkenes catalyzed by **1** was also investigated in detail by DFT calculations using propene and HSiMe_3_ as model substrates. The resulting free energy profiles are represented in Figures 1 and 2 while Scheme 6 depicts the simplified catalytic cycles (only key intermediates are shown).

Catalyst initiation, starting from **1** involving migratory insertion of the propyl ligand into a Mn–CO bond to form an acyl species stabilized by an agostic C–H bond, has been reported previously.^17^ The profile starts with **IN**_**A**_, a van der Waals pair with silane and the metallic fragment. Addition of HSiMe_3_ to the acyl intermediate affords complex **IN**_**B**_ bearing an η^1^-HSiMe_3_ ligand. **IN**_**B**_ is the analogue of **1-Si-H** with the model silane (HSiMe_3_) instead of the real one (HSiMe_2_Ph). They are equivalent by a Si–H distance within 0.01 Å (*d*_Si–H_ = 1.55 Å, in **IN**_**B**_). Addition of HSiMe_3_ to the acyl intermediate affords complex **IN**_**B**_ bearing an η^1^-HSiMe_3_ ligand. Coordination of HSiMe_3_ has a barrier of 11 kcal/mol (**TS**_**INAB**_) and a free energy balance of Δ*G* = 9 kcal/mol. H atom transfer from HSiMe_3_ to the C atom of the acyl ligand produces **IN**_**C**_, a C–H σ-complex of butanal. This step has a barrier of 16 kcal/mol and is endergonic by 13 kcal/mol. Ligand exchange from butanal to one molecule of propene, which is thermodynamically very favorable by −8 kcal/mol, yields the catalytically active species **A** thereby entering the catalytic cycles (Scheme 6).

In the next step of the reaction the silyl ligand in **A** migrates to the terminal olefin C atom resulting in an alkyl complex stabilized by a C–H agostic interaction in intermediate **B**. This is a very facile step with a barrier of 9 kcal/mol and a favorable free energy balance of Δ*G* = −13 kcal/mol. The following step corresponds to a switch between the C–H agostic interaction (reversible *Z* to *E* isomerization) and finally β-hydrogen elimination to afford the hydride *E*-vinylsilene intermediate [Mn(dippe)(CO)_2_(H)(η^2^–CH(SiMe_3_)=CHCH_3_)] (**D**^**E**^). It has to be noted that the formation of the corresponding *Z*-vinylsilane complex is kinetically favored with a barrier that is 8 kcal/mol lower. However, formation of the *E*-product is thermodynamically favored by 2 kcal/mol, reflecting the stability difference between the two free olefin isomers (for details see SI, Figure S16). Accordingly, in agreement with experimental data, *Z* to *E* isomerization takes place readily under the applied reaction conditions and the formation of free *E*-silanes is thermodynamically controlled.

In an acceptorless DS pathway (Scheme 6) addition of HSiMe_3_ to **D**^**E**^ results in the liberation of the *E*-vinylsilane and formation of complex **E** featuring an η^1^-HSiMe_3_ ligand. This process is thermodynamically favored by 4 kcal/mol. H atom transfer from the silane to the hydride ligand generates intermediate **F** bearing a silyl ligand and an η^2^-dihydrogen ligand. This last step has a negligible barrier (3 kcal/mol) and is slightly endergonic with Δ*G* = 3 kcal/mol. Closing of the catalytic cycle brings **F** back to **A** with liberation of dihydrogen and coordination of a new propene molecule in an unfavorable process with Δ*G* = 16 kcal/mol.

In fact, the DFT calculated free energy balance for the formation of product **10** and H_2_ from styrene and HSiMe_2_Ph is clearly positive: Δ*G* = 6.1 kcal/mol (eq S1, SI). However, such conditions are not fulfilled at low hydrogen pressure and accordingly such a reaction becomes feasible (*vide infra*).

The classic DS pathway is initiated upon substitution of the vinylsilane ligand by a new propene molecule resulting in the formation of the hydride alkene complex **G**. In the next step of the reaction the hydride migrates to the internal olefin C atom resulting in an alkyl complex stabilized by a C–H agostic interaction in intermediate **H**. This is a very facile step with a barrier of merely 1 kcal/mol and a favorable free energy balance of Δ*G* = −2 kcal/mol. Alkyl rotation affords intermediate **I** which reacts then with an incoming HSiMe_3_ molecule to the silane alkyl complex **J**. This transformation is endergonic by 11 kcal/mol. In the final step, hydrogen transfer from HSiMe_3_ to the alkyl ligand yields the silyl species **K** featuring a loosely bound propane. Addition of propene regenerates **A** with concomitant liberation of propane thereby closing the catalytic cycle. This step is thermoneutral.

By comparing both pathways, under a hydrogen partial pressure of 1.0 atm (default pressure in GAUSSIAN 09) the classic DS pathway is more favorable by 7 kcal/mol. However, at low hydrogen pressure of *p*_H2_ = 10^–4^, 10^–6^, and 10^–8^ atm, respectively, this value drops from 7 to −3 kcal/mol and thus the acceptorless pathway becomes competitive with the classic DS pathway in the early stage of the reaction (Figure 2). This is in full agreement with experimental findings.

## Conclusion

DS of alkenes displays an interesting approach to synthesize unsaturated silanes. However, the atom efficiency is lowered by the fact that an excess of substrate or addition of sacrificial agents is typically required. We have established a solvent-free manganese-catalyzed DS procedure of terminal alkenes under mild conditions (room temperature) with no additives needed. The most active precatalyst is the bench-stable alkyl bisphosphine Mn(I) complex *fac-*[Mn(dippe)(CO)_3_(CH_2_CH_2_CH_3_)]. The catalytic process is initiated by migratory insertion of a CO ligand into the Mn–alkyl bond to yield an acyl intermediate which undergoes rapid Si–H bond cleavage of the silane HSiR_3_ forming the active 16e^–^ Mn(I) silyl catalyst [Mn(dippe)(CO)_2_(SiR_3_)] together with liberated butanal. The implemented system operates at room temperature without any additives and low catalyst loadings. A broad variety of different styrene derivatives yielding selectivity *E*-vinylsilanes and aliphatic systems, giving allylsilanes, were efficiently reacted with different tertiary silanes. Mechanistic studies which include *in situ* NMR measurements, determination of reaction kinetics, analysis of decomposed active species, and deuterium labeling experiments in addition to computational investigations provided insights into the reaction mechanism. A proposed mechanism is presented following two parallel pathways proceeding under acceptorless conditions involving hydrogen release and *via* a pathway requiring an alkene as a sacrificial hydrogen acceptor. Thus, in many instances the ratio of vinylsilane or allysilane to alkane approaches a 2:1 rather than a 1:1 ratio as normally observed. In contrast to other manganese-based procedures, the reaction proceeds *via* an inner-sphere mechanism rather than *via* radical routes resulting in mild reaction conditions and excellent selectivity.

## Supporting Information Available

Synthetic procedures, ^1^H, ^13^C{^1^H}, and ^31^P{H} NMR spectra of all compounds and crystallographic data (PDF)
Cartesian coordinates for DFT-optimized structures (XYZ)

## Supplementary Material

ja1c09175_si_001.pdf

ja1c09175_si_002.xyz

## Accession Codes

CCDC 2094811 contains the supplementary crystallographic data for this paper. These data can be obtained free of charge *via*www.ccdc.cam.ac.uk/data_request/cif, or by emailing data_request@ccdc.cam.ac.uk, or by contacting The Cambridge Crystallographic Data Centre, 12 Union Road, Cambridge CB2 1EZ, UK; fax: +44 1223 336033.
