# Supplementary material for: Manganese-Catalyzed Dehydrogenative Silylation of Alkenes Following Two Parallel Inner-Sphere Pathways
Source: J Am Chem Soc. 2021 Oct 13;143(42):17825–32. doi: 10.1021/jacs.1c09175 (PMC8554758; doi:10.1021/jacs.1c09175)
Supplement: Supplementary file 1 — ja1c09175_si_001.pdf [file ja1c09175_si_001.pdf]

Manganese-Catalyzed Dehydrogenative Silylation of Alkenes Following Two Parallel Inner-Sphere Pathways

Stefan Weber,[a] Manuel Glavic,[a] Berthold Stöger,[b] Ernst Pittenauer,[c] Maren Podewitz,[d] Luis F. Veiros[e] and Karl Kirchner[a,*]

[a]Institute of Applied Synthetic Chemistry, Vienna University of Technology, Getreidemarkt 9, A-1060 Vienna, AUSTRIA

[b]X-Ray Center, Vienna University of Technology, Getreidemarkt 9, A-1060 Vienna, AUSTRIA

[c]Institute of Chemical Technologies and Analytics, Vienna University of Technology, Getreidemarkt 9, A-1060 Vienna, AUSTRIA

[d]Institute of Materials Chemistry, Vienna University of Technology, Getreidemarkt 9, A-1060 Vienna, AUSTRIA

[e]Centro de Química Estrutural, Instituto Superior Técnico, Universidade de Lisboa, Av. Rovisco Pais No. 1, 1049-001 Lisboa, PORTUGAL

email: karl.kirchner@tuwien.ac.at

Supporting Information

1 Experimental Section S2

1.1 General Information S2

1.2 General Procedure for Catalytic Reactions S3

2 Further Optimization Reactions S3

3 Characterization of Organic Products S4

4 Mechanistic studies S10

4.1 Headspace analysis S10

4.2 KIE experiments S11

4.3 Treatment of Complex 1 with $HSiMe_2Ph$ S14

4.4 Synthesis of 34 .... S17

4.5 NMR analysis upon reaction progress S20

4.6 Investigation of *in-situ E*/*Z*-isomerization S23

5 X-ray structure determination S24

6 Theoretical Calculations S24

7 References S26

8 NMR Spectra of all Organic Products 30

1 Experimental Section

1.1 General Information

All reactions were performed under an inert atmosphere of argon by using Schlenk techniques or in a MBraun inert-gas glovebox. The solvents were purified according to standard procedures. All alkene-substrates and silanes were purchased from Sigma-Aldrich, Acros Organics or TCI and used as purchased without further purification. The deuterated solvents were purchased from Eurisotope and dried over 3 Å molecular sieves. Complexes **1**, **2**,[1] and [DIPPEMn(CO)$_3$H][2] were synthesized according to literature.^{1}H and ^{13}C{^{1}H}, and ^{31}P{^{1}H} NMR spectra were recorded on Bruker AVANCE-250, AVANCE-400, and AVANCE-600 spectrometers. ^{1}H and ^{13}C{^{1}H} NMR spectra were referenced internally to residual protio-solvent, and solvent resonances, respectively, and are reported relative to tetramethylsilane (δ = 0 ppm). ^{31}P{^{1}H} NMR spectra were referenced externally to H_3PO_4 (85%) (δ = 0 ppm).

High resolution-accurate mass data mass spectra were recorded on a hybrid Maxis Qq-aoTOF mass spectrometer (Bruker Daltonics, Bremen, Germany) fitted with an ESI-source. Measured accurate mass data of the $[M]^+$ ions for confirming calculated elemental compositions were typically within ±5 ppm accuracy. The mass calibration was done with a commercial mixture of perfluorinated trialkyl-triazines (ES Tuning Mix, Agilent Technologies, Santa Clara, CA, USA).

GC–MS analysis was conducted on an ISQ LT Single quadrupole MS (Thermo Fisher) directly interfaced to a TRACE 1300 Gas Chromatographic systems (Thermo Fisher), using a Rxi-5Sil MS (30 m, 0.25mm ID) cross-bonded dimethyl polysiloxane capillary column.

Headspace analysis was performed on a Pfeiffer Typ QME 200 Mass-spectrometer equipped with a balzers Prisma and a tungsten filament (details see 4.1).

1.2 General Procedure for Catalytic Reactions

Inside an argon-flushed glovebox, a screwcap-vial (8 mL) was charged with catalyst (0.5-3 mol%), alkene substrate (1.01 mmol, 1.8 equiv.), 0.5 mL solvent (if used) and silane (0.56 mmol, 1 equiv.) in this order. A stirring-bar was added, the vial was sealed, transferred outside the glovebox and the reaction mixture was heated to the indicated temperature (if required) and stirred for the indicated time. The reaction was quenched by exposure to air. 1 µl of the sample was taken for GC-MS analysis.

Isolation of product

After exposing the sample to air for 1 h, the product was purified by filtration though a pad of silica (approx. 1.5 g, silica 60 µm) followed by elution with *n*-pentane (4-5 mL) and careful removement of the solvent, if not stated otherwise.

2 Further Optimization Reactions

Table S 1. Additional Optimization Reactions

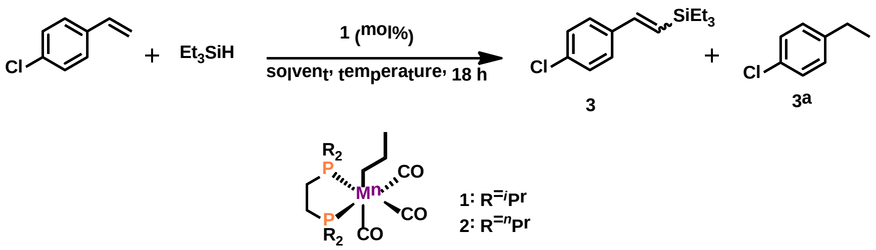

entry	catalyst loading (mol%)	Temperature (°C)	Solvent	Conversion (%)	E/Z ratio	**3**:**3a**
1	3	70	Toluene	81	98:2	1.9:1
2	3	70	DME	91	96:4	2:1
3	3	70	$CHCl_3$	traces	-	-
4	3	70	iPrOH	traces	-	-
5[b]	2	70	THF	>99	97:3	2:1
6[b]	1	70	THF	>99	>99:1	1.8:1
7[b,c]	2	50	THF	>99	97:3	2:1
8[b,c]	2	25	THF	>99	99:1	2:1
9[b,c]	1	25	neat	89	>99:1	2.1:1

Reaction conditions: triethylsilane (0.56 mmol, 1 equiv.), 4-chlorostyrene (1.12 mmol, 2 equiv.), 0.5 ml anhydrous solvent, 18 h. Conversion of silane, E/Z- and ratio of **3**:**3a** determined by GC/MS. [b]4-Chlorostyrene (0.84 mmol, 1.5 equiv.). [c]24h.

3 Characterization of Organic Products

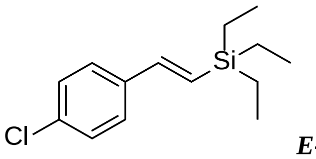

***E*-(4-chlorostyryl)triethylsilane[3] (3)**, colorless oil, 133 mg (94 %) ^{1}H NMR (δ, 400 MHz, CD_2Cl_2, 20 °C): 7.31 (d, *J* = 8.4 Hz, 2H), 7.22 (d, *J* = 8.6 Hz, 2H), 6.78 (d, *J* = 19.3 Hz, 1H), 6.35 (d, *J* = 19.3 Hz, 1H), 0.90 (t, *J* = 7.9 Hz, 9H), 0.59 (q, *J* = 16.5 Hz, 6H). ^{13}C{^{1}H} NMR (δ, 101 MHz, CD_2Cl_2, 20°C): 143.8, 137.6, 133.71, 129.0, 127.9, 127.5, 7.5, 3.8.

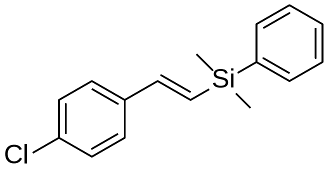

***E*-(4-chlorostyryl)dimethyl(phenyl)silane[4] (4)**, colorless oil, 149 mg (98 %) ^{1}H NMR (δ, 250 MHz, CD_2Cl_2, 20 °C):7.66 – 7.58 (m, 3H), 7.49 – 7.31 (m, 7H), 6.96 (d, *J* = 19.2 Hz, 1H), 6.64 (d, *J* = 19.1 Hz, 1H), 0.49 (s, 6H). ^{13}C{^{1}H} NMR (δ, 101 MHz, CD_2Cl_2, 20°C): 146.1, 140.7, 139.2, 136.2, 136.0, 131.4, 131.0, 130.6, 130.2, 130.1, -0.6.

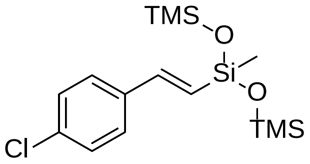

***E*-3-(4-chlorostyryl)-1,1,1,3,5,5,5-heptamethyltrisiloxane[5] (5)** colorless oil, 187 mg (93 %) ^{1}H NMR (δ, 250 MHz, CD_2Cl_2, 20 °C): 7.41 (d, *J* = 8.5 Hz, 2H), 7.33 (d, *J* = 8.8 Hz, 2H), 6.94 (d, *J* = 19.2 Hz, 1H), 6.28 (d, *J* = 19.2 Hz, 1H), 0.20 (s, 3H), 0.15 (s, 18H). ^{13}C{^{1}H} NMR (δ, 101 MHz, CD_2Cl_2, 20°C): 142.9, 136.2, 133.1, 128.0, 127.2, 126.9, 0.9, -1.0.

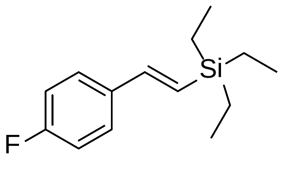

***E*-(4-fluorostyryl)triethylsilane[6] (6)**, colorless oil, 121 mg (91 %) %) ^{1}H NMR (δ, 400 MHz, CD_2Cl_2, 20 °C): 7.34 (dd, *J* = 8.7, 5.6 Hz, 2H), 6.93 (t, *J* = 8.7 Hz, 2H), 6.78 (d, *J* = 19.3 Hz, 1H), 6.27 (d, *J* = 19.3 Hz, 1H), 0.90 (t, *J* = 7.9 Hz, 9H), 0.58 (q, *J* = 7.9 Hz, 6H). 162.8 (d, J_{C-F} = 256.5 Hz), 144.2, 135.2, 128.0, 126.1, 115.6, 115.4, 7.4, 3.7. ^{19}F{^{1}H} NMR (δ, 235 MHz, CD_2Cl_2, 20°C): -115.0.

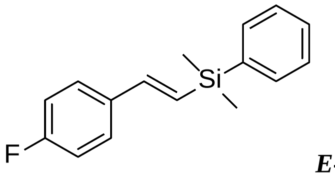

***E*-(4-fluorostyryl)dimethyl(phenyl)silane[4] (7)**, colorless oil, 140 mg (97 %) ^{1}H NMR (δ, 250 MHz, CD_2Cl_2, 20 °C): 7.64 – 7.60 (m, 2H), 7.52 – 7.49 (m, 3H), 7.43 – 7.39 (m, 2H), 7.08 (t, *J* = 8.8 Hz, 2H), 6.98 (d, *J* = 19.5 Hz, 1H), 6.57 (d, *J* = 19.2 Hz, 1H), 0.48 (s, 6H). ^{13}C{^{1}H} NMR (δ, 101 MHz, CD_2Cl_2, 20°C): 163.1, (d, J_{C-F} = 248.5 Hz) 144.3, 139.0, 135.1, 134.3, 129.9, 129.5, 128.6, 128.5, 128.3, 127.4, 115.9, 115.7, -2.4. ^{19}F{^{1}H} NMR (δ, 235 MHz, CD_2Cl_2, 20°C): -114.5.

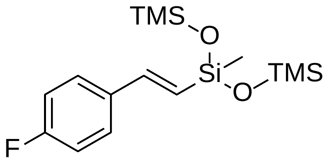

***E*-3-(4-fluorostyryl)-1,1,1,3,5,5,5-heptamethyltrisiloxane[5] (8)** colorless oil, 187 mg (96 %) ^{1}H NMR (δ, 250 MHz, CD_2Cl_2, 20 °C):7.51 – 7.40 (m, 2H), 7.08 – 7.01 (m, 2H), 6.95 (d, *J* = 18.7 Hz, 1H), 6.21 (d, *J* = 19.2 Hz, 1H), 0.20 (s, 3H), 0.15 (s, 18H). ^{13}C{^{1}H} NMR (δ, 101 MHz, CD_2Cl_2, 20°C): 163.3 (d, $J_{C\text{-}F}$ = 248.5 Hz), 144.1, 135.0, 128.6, 128.6, 126.9, 115.9, 115.67, 2.0, 0.1. ^{19}F{^{1}H} NMR (δ, 235 MHz, CD_2Cl_2, 20°C): -141.4.

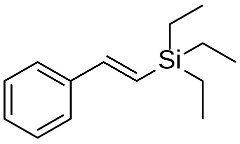

***E*-triethyl(styryl)silane[6] (9)** colorless oil, 117 mg (96 %) ^{1}H NMR (δ, 250 MHz, CD_2Cl_2, 20 °C):7.53 – 7.45 (m, 2H), 7.42 – 7.24 (m, 3H), 6.97 (d, *J* = 19.4 Hz, 1H), 6.50 (d, *J* = 19.3 Hz, 1H), 1.05 (t, *J* = 7.9 Hz, 8H), 0.73 (q, *J* = 7.3 Hz, 6H). ^{13}C{^{1}H} NMR (δ, 101 MHz, CD_2Cl_2, 20°C): 145.5, 139.3, 129.1, 128.5, 126.9, 126.6, 7.9, 4.1.

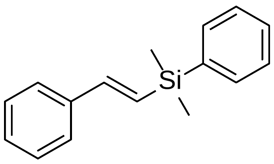

***E*-dimethyl (phenyl)(styryl)silane[4] (10)**, colorless oil, 130 mg (97 %) ^{1}H NMR (δ, 250 MHz, CD_2Cl_2, 20 °C):7.70 – 7.59 (m, 2H), 7.58 – 7.48 (m, 3H), 7.48 – 7.30 (m, 5H), 7.03 (d, *J* = 19.2 Hz, 1H), 6.67 (d, *J* = 19.1 Hz, 1H), 0.50 (s, 6H). ^{13}C{^{1}H} NMR (δ, 101 MHz, CD_2Cl_2, 20°C): 145.5, 139.0, 138.6, 134.2, 129.3, 128.8, 128.5, 128.1, 127.4, 126.7, -2.5.

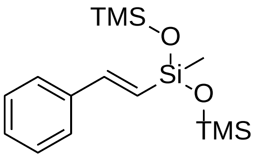

***E*-1,1,1,3,5,5,5-heptamethyl-3-styryl-trisiloxane[5] (11)** colorless oil, 172 mg (95 %) ^{1}H NMR (δ, 250 MHz, CD_2Cl_2, 20 °C):7.55 – 7.43 (m, 2H), 7.41 – 7.23 (m, 3H), 7.00 (d, *J* = 19.2 Hz, 1H), 6.30 (d, *J* = 19.2 Hz, 1H), 0.21 (s, 3H), 0.16 (s, 17H). ^{13}C{^{1}H} NMR (δ, 101 MHz, CD_2Cl_2, 20°C): 145.7, 138.9, 129.2, 129.0, 127.3, 127.2, 2.3, 0.4.

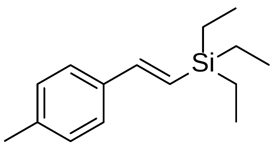

***E*-triethyl(4-methylstyryl)silane[3] (12)**, colorless oil, 124 mg (95 %) ^{1}H NMR (δ, 400 MHz, CD_2Cl_2, 20 °C):7.38 (d, *J* = 8.3 Hz, 2H), 7.18 (d, *J* = 7.9 Hz, 2H), 6.92 (d, *J* = 19.3 Hz, 1H), 6.41 (d, *J* = 19.3 Hz, 1H), 2.37 (s, 3H), 1.03 (t, *J* = 7.9 Hz, 9H), 0.71 (q, *J* = 7.6 Hz, 6H). ^{13}C{^{1}H} NMR (δ, 101 MHz, CD_2Cl_2, 20°C): 144.7, 137.9, 135.9, 129.1, 126.1, 124.4, 20.9, 7.2, 3.5.

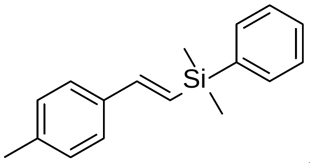

***E*-dimethyl (4-methyl) (phenyl)silane[4] (13)**, colorless oil, 136 mg (96 %) ^{1}H NMR (δ, 250 MHz, CD_2Cl_2, 20 °C): 7.73 – 7.58 (m, 2H), 7.42 – 7.39 (m, 6H), 7.20 (d, *J* = 7.0 Hz, 2H), 6.99 (d, *J* = 19.2 Hz, 1H), 6.59 (d, *J* = 19.1 Hz, 1H), 2.39 (s, 3H), 0.48 (s, 6H). ^{13}C{^{1}H} NMR (δ, 101 MHz, CD_2Cl_2, 20°C): 145.6, 139.3, 138.7, 136.0, 134.4, 129.7, 129.4, 128.2, 126.8, 126.1, 21.0, -2.4.

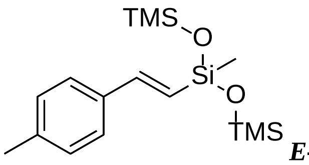

***E*-1,1,1,3,5,5,5-heptamethyl-3-(-methylstyryl)-trisiloxane[5] (14)** colorless oil, 176 mg (93 %) ^{1}H NMR (δ, 250 MHz, CD_2Cl_2, 20 °C):7.36 (d, *J* = 8.1 Hz, 2H), 7.17 (d, *J* = 7.6 Hz, 2H), 6.96 (d, *J* = 19.3 Hz, 1H), 6.23 (d, *J* = 19.2 Hz, 1H), 2.35 (s, 3H), 0.20 (s, 3H), 0.15 (s, 18H). ^{13}C{^{1}H} NMR (δ, 101 MHz, CD_2Cl_2, 20°C): 145.4, 138.8, 135.9, 129.6, 126.9, 125.6, 21.4, 2.0, 0.2.

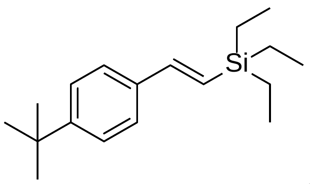

***E*-triethyl(4-(*tert-butyl)*styryl)silane[3] (15)**, colorless oil, 146 mg (95 %) ^{1}H NMR (δ, 400 MHz, CD_2Cl_2, 20 °C):7.32 – 7.24 (m, 4H), 6.80 (d, *J* = 19.3 Hz, 1H), 6.30 (d, *J* = 19.3 Hz, 1H), 1.22 (s, 9H), 0.90 (t, *J* = 7.9 Hz, 9H), 0.58 (q, *J* = 7.9 Hz, 6H). ^{13}C{^{1}H} NMR (δ, 101 MHz, CD_2Cl_2, 20°C): 151.1, 144.6, 135.9, 125.9, 125.4, 124.7, 31.0, 7.15, 3.5.

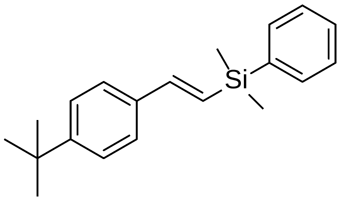

***E*-(4-(*tert-butyl)*styryl)dimethyl(phenyl)silane[7] (16)**, colorless oil, 160 mg (97 %) ^{1}H NMR (δ, 250 MHz, CD_2Cl_2, 20 °C):7.69 – 7.64 (m, 3H), 7.49 -7.41 (m, 6H), 7.04 (d, *J* = 19.1 Hz, 1H), 6.64 (d, *J* = 19.1 Hz, 1H), 1.39 (s, 12H), 0.52 (s, 6H). ^{13}C{^{1}H} NMR (δ, 101 MHz, CD_2Cl_2, 20°C): 152.2, 145.8, 139.58, 136.3, 134.6, 129.7, 128.5, 126.9, 126.7, 126.2, 35.2, 31.8, -2.0.

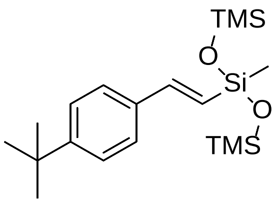

***E*-3-(4-(*tert-butyl)*styryl)-1,1,1,3,5,5,5-heptamethyl-trisiloxane[5] (17)** colorless oil, 187 mg (88 %) ^{1}H NMR (δ, 250 MHz, CD_2Cl_2, 20 °C):7.48 – 7.39 (m, 4H), 6.99 (d, *J* = 19.2 Hz, 1H), 6.26 (d, *J* = 19.2 Hz, 1H), 1.34 (s, 9H), 0.22 (s, 3H), 0.17 (s, 18H). ^{13}C{^{1}H} NMR (δ, 101 MHz, CD_2Cl_2, 20°C): 151.6, 144.8, 135.4, 126.2, 125.5, 34.5, 31.0, 1.6, -0.3.

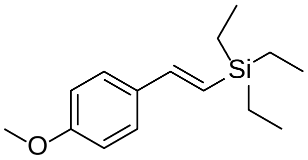

***E*-(4-methoxy*)*styryl)dimethyl(phenyl)silane[8] (18)**, colorless oil, 133 mg (97 %) ^{1}H NMR (δ, 250 MHz, CD_2Cl_2, 20 °C):7.40 (d, *J* = 8.9 Hz, 2H), 6.88 (d, J = 19.2 Hz, 1H), 6.87 (d, J = 8.9 Hz, 2H) , 6.28 (d, *J* = 19.3 Hz, 1H), 3.81 (s, 3H), 1.01 (t, J = 8.3 Hz, 9H), 0.68 (q, *J* = 7.7 Hz, 6H). ^{13}C{^{1}H} NMR (δ, 101 MHz, CD_2Cl_2, 20°C): 160.2, 144.8, 132.1, 129.3, 128.1, 123.5, 114.4, 55.8, 7.8, 4.1.

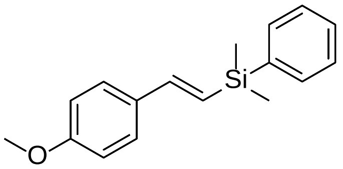

***E*-(4-methoxy*)*styryl)dimethyl(phenyl)silane[4] (19)**, colorless oil, 140 mg (93 %) ^{1}H NMR (δ, 250 MHz, CD_2Cl_2, 20 °C):7.66 – 7.53 (m, 2H), 7.46 – 7.33 (m, 4H), 6.99 – 6.79 (m, 3H), 6.45 (d, *J* = 19.2 Hz, 1H), 3.82 (s, 3H), 0.45 (s, 6H). ^{13}C{^{1}H} NMR (δ, 101 MHz, CD_2Cl_2, 20°C): 160.2, 145.1, 139.3, 134.3, 131.5, 129.3, 129.0, 128.1, 124.5, 114.2, 56.3, -2.4.

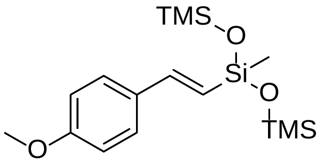

***E*-3-(4-methoxystyryl)-1,1,1,3,5,5,5-heptamethyl-trisiloxane[5] (20)** colorless oil, 181 mg (88 %) ^{1}H NMR (δ, 250 MHz, CD_2Cl_2, 20 °C):7.41 (d, *J* = 8.8 Hz, 2H), 6.94 (d, J = 19.5 Hz, 1H) 6.98 (d, J = 8.6 Hz), 6.12 (d, *J* = 19.2 Hz, 1H), 3.82 (s, 3H), 0.19 (s, 3H), 0.15 (s, 13H). ^{13}C{^{1}H} NMR (δ, 101 MHz, CD_2Cl_2, 20 °C): 160.4, 144.9, 131.4, 128.2, 124.2, 114.3, 2.0, -0.4.

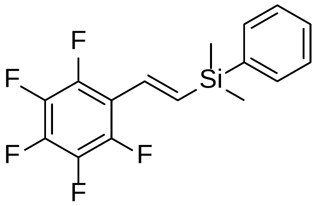

***E*-(pentafluorostyryl)dimethyl(phenyl)silane (21)**, colorless oil, 160 mg (87 %) ^{1}H NMR (δ, 250 MHz, CD_2Cl_2, 20 °C): 7.68 – 7.54 (m, 2H), 7.48 – 7.34 (m, 3H), 7.00 (d, *J* = 20.1 Hz, 1H), 6.83 (d, *J* = 19.7 Hz, 1H), 0.49 (s, 6H). ^{13}C{^{1}H} NMR (δ, 101 MHz, CD_2Cl_2, 20°C): 146.7 - 146.5 (m, C-F), 144.2 - 144.1 (m, C-F), 141.9 – 141.7 (m, C-F), 140.5 – 140.4 (m, C-F), 140.1 – 139.9 (m, C-F), 137.7, 137.0 – 136.7 (m, C-F), 134.3, 133.4, 129.7, 128.8, 128.7, 128.3, 128.1, -2.9. ^{19}F{^{1}H} NMR (δ, 235 MHz, CD_2Cl_2, 20°C): -144.9 – -145.0 (m), -156.9(t, J= 21.5 Hz), -163.9– -164.2 (m). Elemental Analysis: calculated for [$C_{16}H_{13}F_5Si$]: C, 58.53; H, 3.99. found: C, 58.49; H, 4.01.

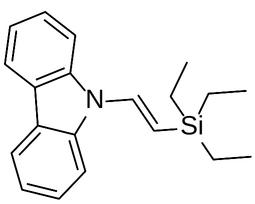

E-9-(2-(triethylsilyl)vinyl)-9H-carbazole (23), purified *via* column chromatography, using 10 g silca (60 µm) as solid phase and *n*-pentane/Et_2O 40:1 as eluent, $R_{f(product)}$= 0.84, colorless oil, 143 mg

(83 %) ^{1}H NMR (δ, 250 MHz, CD_2Cl_2, 20 °C): 8.11 (d, *J* = 6.5 Hz, 2H), 7.75 (d, *J* = 9.1 Hz, 2H), 7.51 (ddd, *J* = 8.4, 7.2, 1.3 Hz, 2H), 7.38 – 7.30 (m, 3H), 6.01 (d, *J* = 17.3 Hz, 1H), 1.12 (t, *J* = 7.9 Hz, 9H), 0.82 (q, *J* = 7.9 Hz, 6H). ^{13}C{^{1}H} NMR (δ, 101 MHz, CD_2Cl_2, 20°C): 140.0, 135.1, 126.9, 124.7, 121.4, 120.9, 111.4, 111.0, 8.0, 4.4. Elemental Analysis: calculated for [$C_{20}H_{25}NSi$]: C, 78.12; H, 8.19; N, 4.55. found: C, 78.15; H 8.17; N, 4.55.

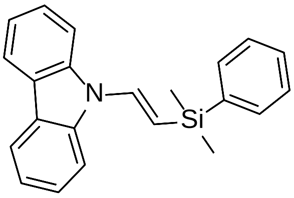

***E*-9-(2-(dimethyl(phenyl)silyl)vinyl)-9H-carbazole[9] (24)**, purified *via* column chromatography, using 10 g silca (60 µm) as solid phase and *n*-pentane/Et_2O 40:1 as eluent, $R_{f(product)}$= 0.81, colorless waxy solid, 160 mg (79 %) ^{1}H NMR (δ, 250 MHz, CD_2Cl_2, 20 °C): 8.13-8.09 (m, 2H), 7.79 – 7.67 (m, 3H), 7.56 – 7.24 (m, 8H), 6.21 (d, *J* = 17.2 Hz, 1H), 0.60 (s, 6H). ^{13}C{^{1}H} NMR (δ, 63 MHz, CD_2Cl_2, 20°C): 139.3, 135.1, 134.0, 129.2, 127.9, 126.3, 125.8, 124.1, 120.9, 120.2, 120.1, 119.8, 110.9, 110.6, 110.2, -2.3.

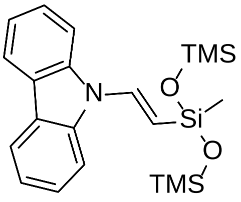

***E*-9-(2-(1,1,1,3,5,5,5-heptamethyltrisiloxan-3-yl)vinyl)-9H-carbazole) (25)**, purified *via* column chromatography, using 10 g silica (60 µm) as solid phase and *n*-pentane/Et_2O 40:1 as eluent, $R_{f(product)}$= 0.78, colorless oil, 201 mg (87 %) ^{1}H NMR (δ, 400 MHz, CD_2Cl_2, 20 °C): 8.13 (d, *J* = 7.0 Hz, 2H), 7.79 (d, *J* = 8.3 Hz, 2H), 7.59 (d, *J* = 17.1 Hz, 1H), 7.54 (d, *J* = 7.1 Hz, 2H), 7.39 – 7.34 (m, 2H), 5.94 (d, *J* = 17.2 Hz, 1H), 0.35 (s, 3H), 0.26 (s, 18H). ^{13}C{^{1}H} NMR (δ, 63 MHz, CD_2Cl_2, 20°C): 133.9, 135.9, 127.0, 124.8, 121.5, 120.8, 111.5, 110.6, 2.3, 1.0. Elemental Analysis: calculated for [$C_{21}H_{31}NO_2Si_3$]: C, 60.96; H, 7.55; N, 3.39. found: C, 60.97; H, 7.53; N, 3.41.

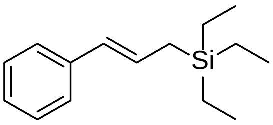

***E*-cinnamyltriethylsilane[10] (26)**, colorless oil, 113 mg (87 %) ^{1}H NMR (δ, 250 MHz, CD_2Cl_2, 20 °C): 7.32-7.11 (m, 5H), 6.37-6.22 (m, 2H), 1.74 (d, J = 7.6 Hz, 2H), 1.00 (t, J = 8.0 Hz, 9H), 0.68 (q, *J* = 7.8 Hz, 6H). ^{13}C{^{1}H} NMR (δ, 63 MHz, CD_2Cl_2, 20°C): 139.3, 129.1, 128. 9, 128.6, 126.8, 126.0, 19.0, 7.7, 3.9.

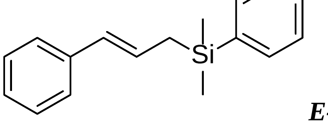

***E*-cinnamyldimethyl(phenyl)silane[11] (27)**, colorless oil, 132 mg (93 %) ^{1}H NMR (δ, 250 MHz, CD_2Cl_2, 20 °C): 7.60 – 7.18 (m, 10H), 6.43 – 6.19 (m, 2H), 2.01 – 1.93 (m, 2H), 0.39 (s, 6H). ^{13}C{^{1}H} NMR (δ, 63 MHz, CD_2Cl_2, 20°C): 138.9, 138.8, 134.0, 133.90, 133.4, 129.5, 129.4, 129.2, 128.8, 128.6, 128.2, 128.1, 128.0, 127.6, 126.6, 125.8, 125.7, 23.3, -3.2.

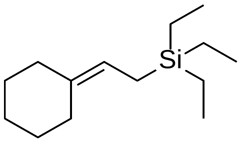

(2-cyclohexylideneethyl)triethylsilane[12] (29), colorless oil, 93 mg (74 %) ^{1}H NMR (δ, 250 MHz, CD_2Cl_2, 20 °C): 5.10 (tt, J = 8.5, 1.2 Hz, 1H), 2.14 – 2.04 (m, 4H), 1.57 – 1.46 (m, 6H), 0.95 (t, J = 7.9 Hz, 9H), 0.54 (q, *J* = 8.3 Hz, 6H). $^{13}C\{^1H\}$ NMR (δ, 63 MHz, CD_2Cl_2, 20°C): 136.4, 116.8, 37.3, 28.8, 28.3, 27.6, 27.1, 12.4, 7.2, 3.2.

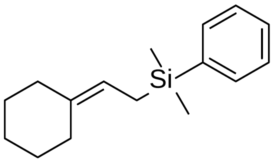

(2-cyclohexylideneethyl)dimethyl(phenyl)silane[13] (30), colorless oil, 125 mg (91 %) ^{1}H NMR (δ, 250 MHz, CD_2Cl_2, 20 °C): 7.62 – 7.47 (m, 2H), 7.38 – 7.34 (m, 3H), 5.11 (t, *J* = 8.5 Hz, 1H), 2.06 (q, *J* = 6.6, 5.4 Hz, 2H), 1.66 (d, *J* = 8.5 Hz, 2H), 1.56 – 1.33 (m, 8H), 0.28 (s, 6H). $^{13}C\{^1H\}$ NMR (δ, 63 MHz, CD_2Cl_2, 20°C): 137.6, 133.6, 133.0, 129.2, 128.8, 127.6, 116.0, 37.3, 28.8, 28.3, 27.5, 27.0, 16.7, -3.5.

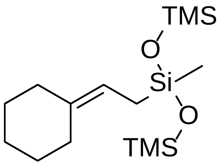

3-(2-cyclohexylideneethyl)-1,1,1,3,5,5,5-heptamethyltrisiloxane[14] (31) colorless oil, 79 mg (43 %) ^{1}H NMR (δ, 250 MHz, CD_2Cl_2, 20 °C):5.08 (t, *J* = 8.3 Hz, 1H), 2.10 (t, *J* = 5.2 Hz, 4H), 1.53 (s, 7H), 1.39 (d, *J* = 8.3 Hz, 2H), 0.14 (m, 3H), 0.11 (s, 18H). $^{13}C\{^1H\}$ NMR (δ, 63 MHz, CD_2Cl_2, 20°C): 137.5, 115.5, 37.3, 28.7, 28.4, 27.6, 27.0, 18.6, 1.5, -0.9.

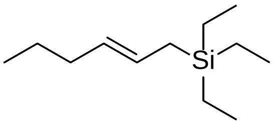

***E*-triethyl(hex-2-en-1-yl)silane [10] (32)**, colorless oil, 64 mg (58 %) ^{1}H NMR (δ, 250 MHz, C_6D_6, 20 °C): 5.49 – 5.19 (m, 2H), 2.00 – 1.87 (m, 2H), 1.50 – 1.38 (m, 2H), 1.38 – 1.23 (m, 2H), 1.01 – 0.79 (m, 12H), 0.48 (q, *J* = 8.4 Hz, 6H). $^{13}C\{^1H\}$ NMR (δ, 63 MHz, C_6D_6, 20°C): 128.7, 126.2, 35.0, 23.2, 17.3, 13.5, 7.3, 3.2.

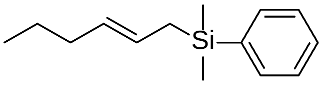

***E*-hex-2-en-1-yldimethyl(phenyl)silane[15] (33)** colorless oil, 100 mg (82 %) ^{1}H NMR (δ, 250 MHz, $CDCl_3$, 20 °C): 7.56 – 7.45 (m, 2H), 7.39 – 7.30 (m, 2H), 5.50 – 5.15 (m, 2H), 2.01 – 1.79 (m, 2H), 1.75 – 1.58 (m, 2H), 1.32 (h, *J* = 7.2 Hz, 2H), 0.85 (t, *J* = 7.1 Hz, 3H), 0.25 (s, 6H). $^{13}C\{^1H\}$ NMR (δ, 63 MHz, $CDCl_3$, 20°C): 133.7, 129.7, 128.9, 128.3, 127.7, 125.5, 124.7, 34.9, 23.1, 21.6, 13.7, -3.4.

4 Mechanistic studies

4.1 Headspace analysis

Inside an argon-flushed glovebox, a screwcap-vial (8 mL) was charged with **1** (5mg, 2mol%), styrene (116 µL, 1.01 mmol, 1.8 equiv) and phenyldimethylsilane (86.1 µL, 0.56 mmol, 1 equiv.) in this order. A stirring-bar was added, the vial was sealed with a cap, containing a septum, transferred outside the glovebox and the reaction mixture was stirred for 3h. 0. 6 mL of the headspace were taken up by a syringe and ejected into the MS.

Pressure in MS: 2 x 10^{-6} mbar without gas mixture (0.0 mbar), 3.5 x 10^{-5} mbar with sample (8.3 mbar)

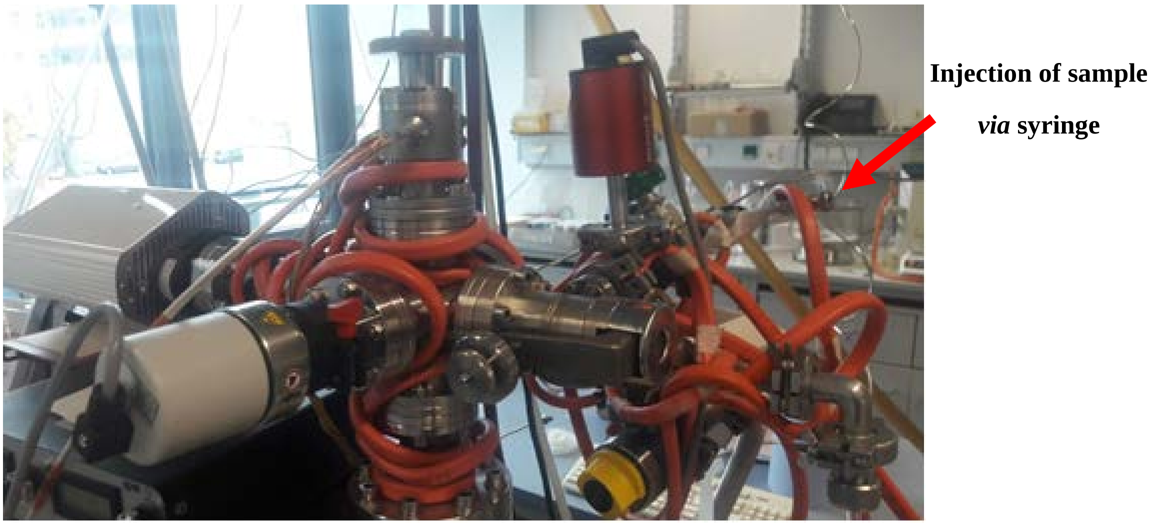

Figure S 1. Picture of MS-setup for headspace analysis.

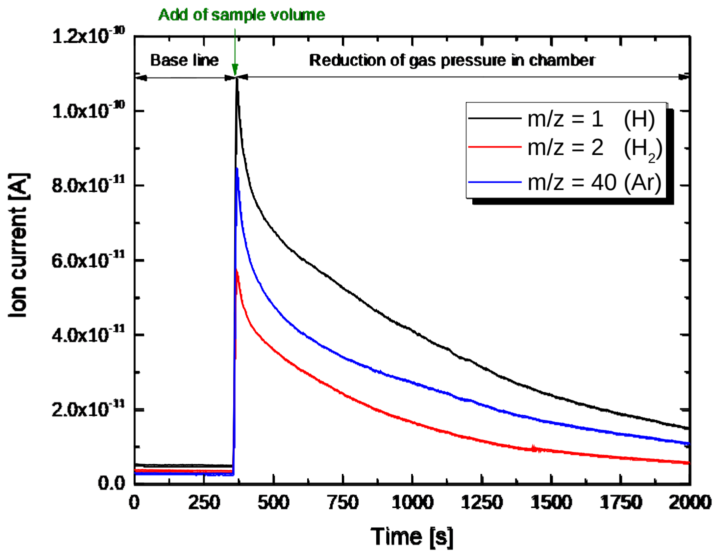

Figure S 2. Detection of Hydrogen in Headspace of sample.

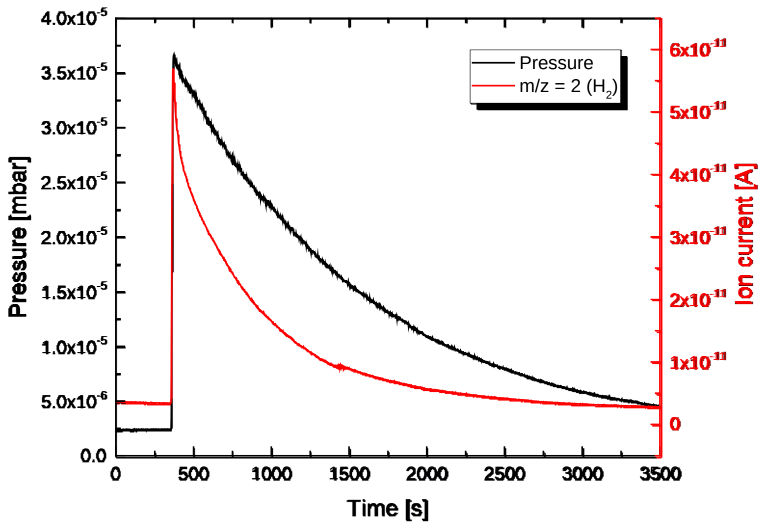

Figure S3. Detection of Hydrogen in Headspace of sample.

4.2 KIE experiments

Inside an argon-flushed glovebox, a screwcap-vial (8 mL) was charged with **1** (5mg, 2 mol%), styrene (116 µL, 1.01 mmol, 1.8 equiv.) and phenyldimethylsilane (86.1 µL, 0.56 mmol, 1 equiv.) in this order. A stirring-bar was added, the vial was sealed with a cap and the mixture was stirred for 1 hour. 1 µl of the sample was taken for GC-MS analysis. After 24 h, 40 µl of the sample was taken and ^{1}H- and $^{13}C\{^{1}H\}$-NMR were measured to determine the incorporation of hydrogen or deuterium in the reaction mixture.

In case of deuterated silane (phenyldimethylsilane-*d*, >99% *D*)[16a] 77.5 mg (0.56 mmol, 1 equiv.) and in case of Styrene-*d8* (>98% D, purchases from Acros Organics) 113.7 mg (1.01 mmol, 1.8 equiv., 98% D) were used.

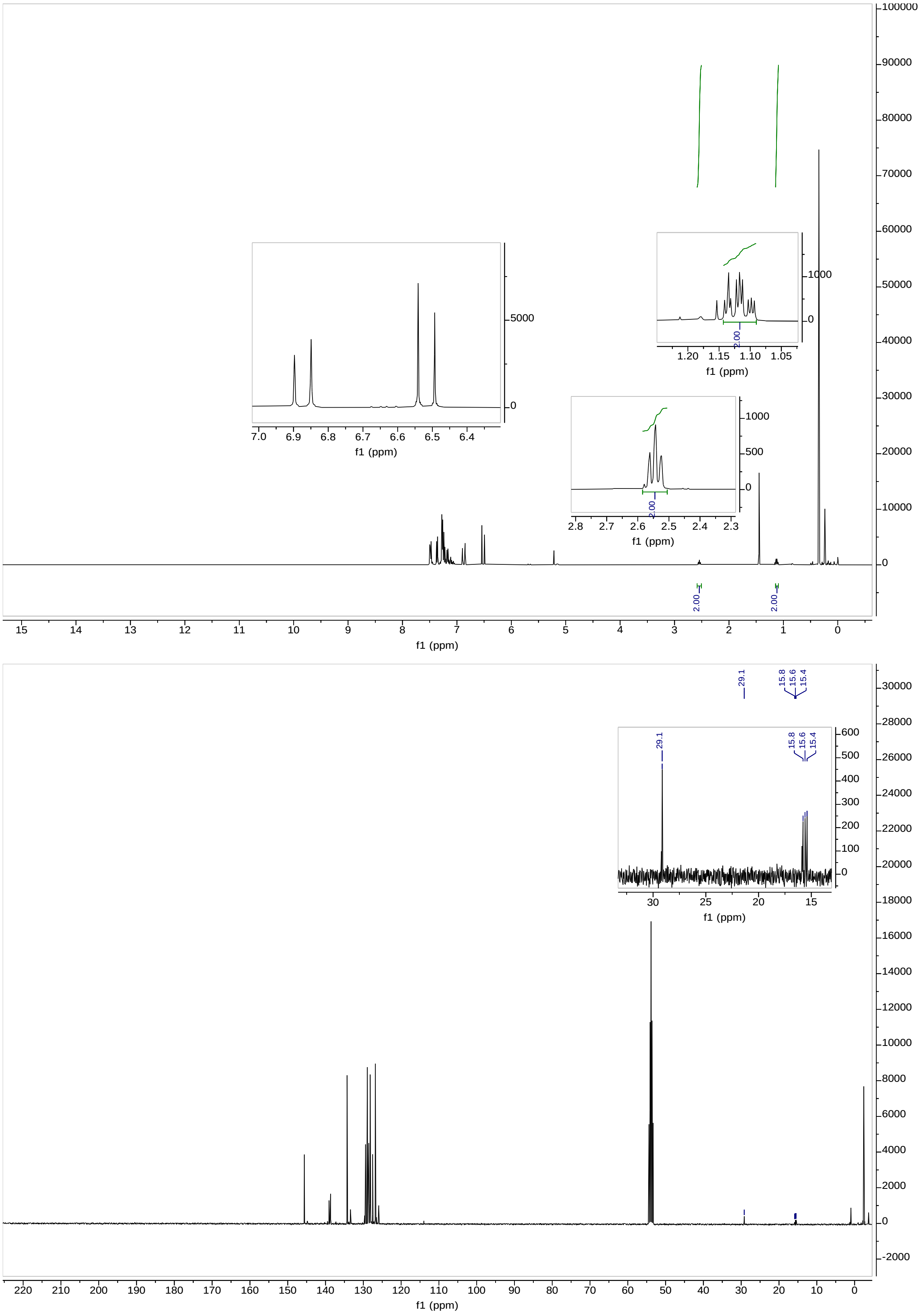

Figure S 4. ^{1}H- and $^{13}C\{^{1}H\}$-NMR analysis for reaction mixture of Styrene with $PhMe_2SiD$ in CD_2Cl_2

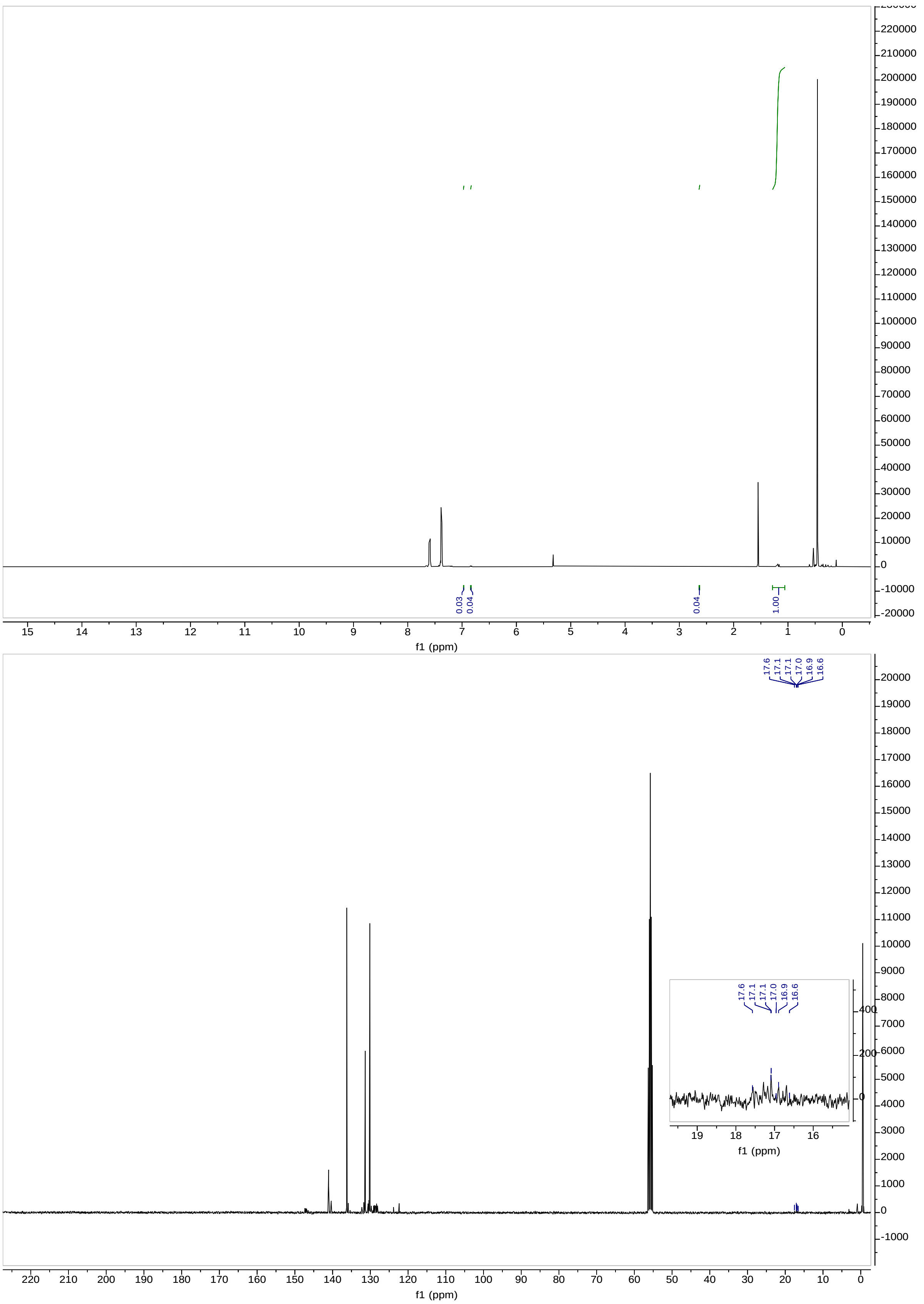

Figure S 5. ^{1}H- and $^{13}C\{^{1}H\}$-NMR analysis for reaction mixture of Styrene-*d8* with $HSiMe_2Ph$ in CD_2Cl_2

4.3 Treatment of Complex 1 with $HSiMe_2Ph$

Inside an argon-flushed glove box, complex **1** (10 mg) was dissolved in 0.6 mL THF-*d8* and transferred into an NMR tube. Phenyldimethylsilane (7 µL, 1 equiv.) was added, resulting in a colorless solution. The NMR tube was sealed and transferred out of the glove box. ^{1}H-,$^{31}P\{^{1}H\}$- and $^{1}H/^{31}P$-HMBC were periodically measured. After approx. 30 min, the color of the solution changed to a bright yellow.

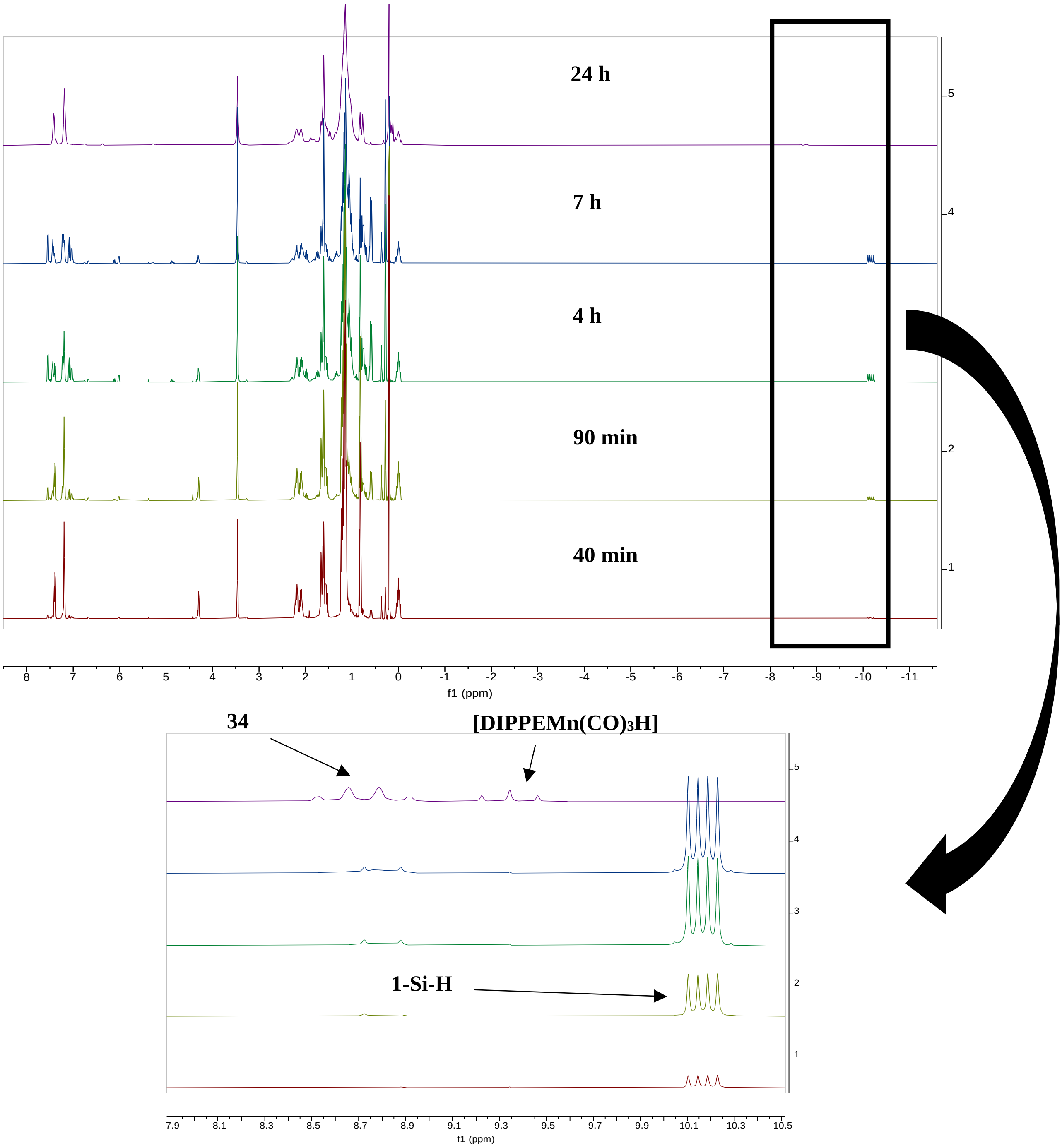

Figure S 6. ^{1}H-NMR during reaction of **1** with 1 equiv. $HSiMe_2Ph$

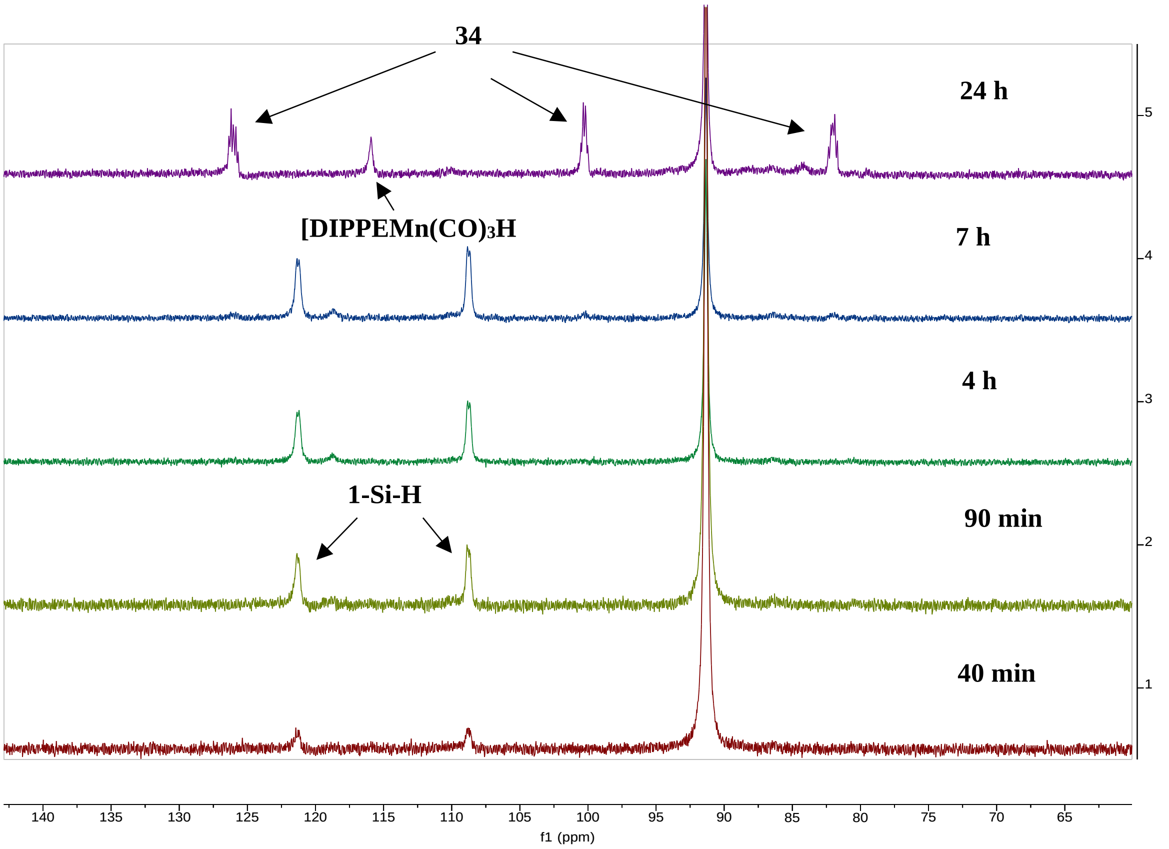

Figure S 7. $^{31}P\{^{1}H\}$-NMR during reaction of **1** with 1 equiv. $HSiMe_2Ph$.

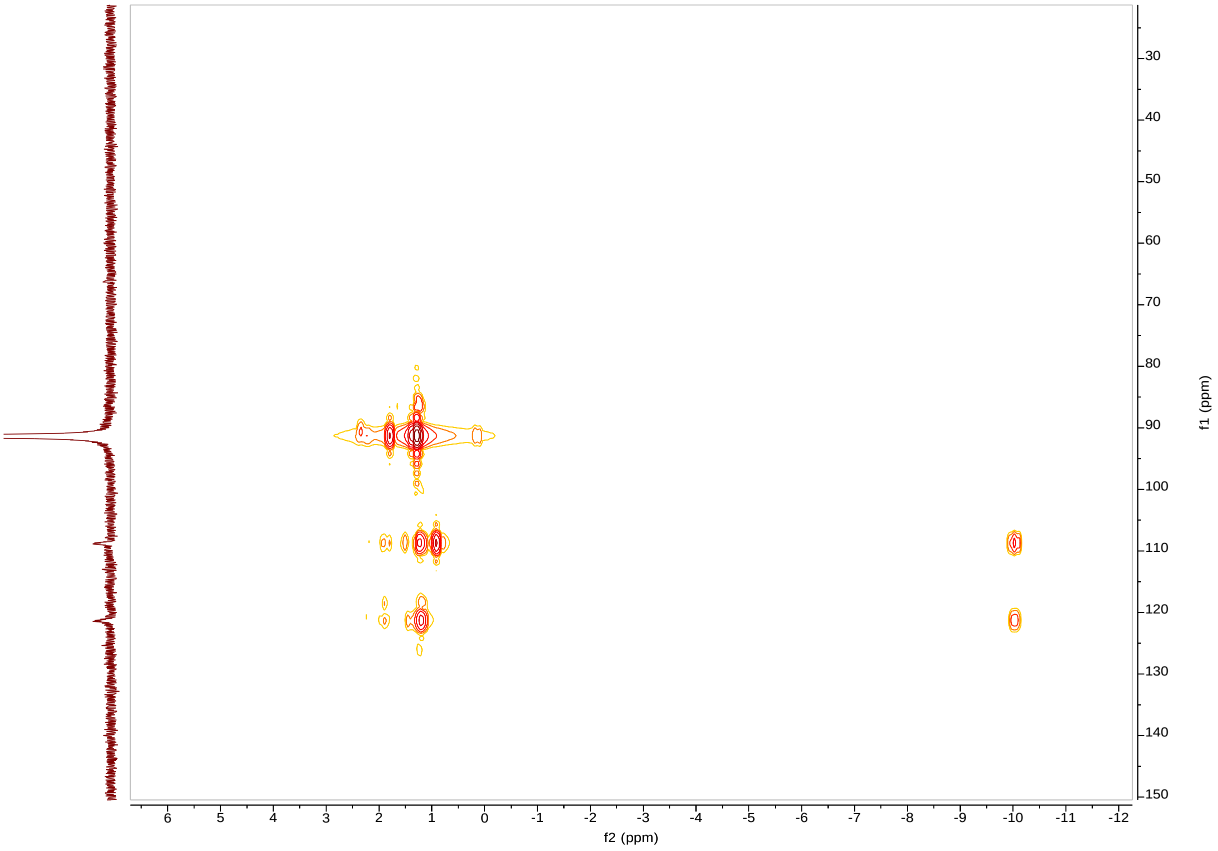

Figure S 8. $^{1}H/^{31}P$-HMBC-NMR of **1** with 1 equiv. $HSiMe_2Ph$ after 4 h.

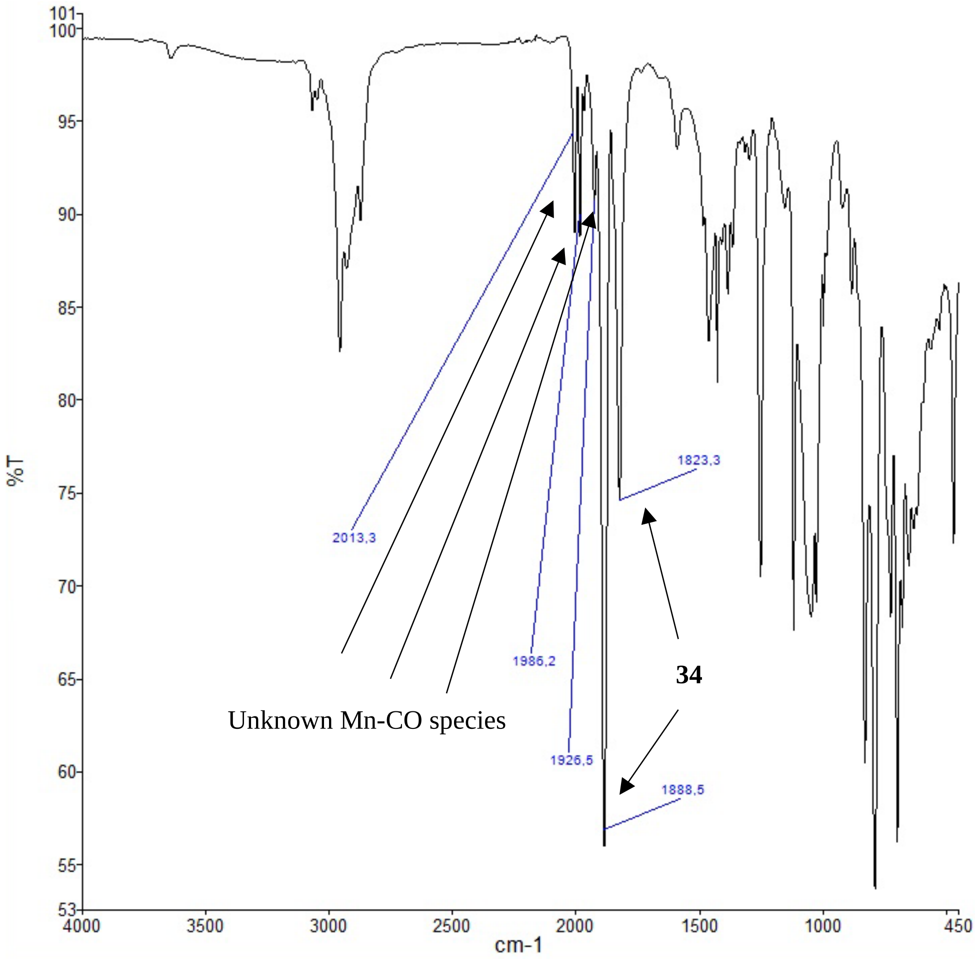

Figure S 9. ATR-IR analysis of stochiometric reaction of **1** with 1 equiv. $HSiMe_2Ph$

4.4 Synthesis of 34

Inside an argon-flushed glove box, complex **1** (20 mg) was dissolved in 0.6 mL THF-*d8* and transferred into an NMR tube. Phenyldimethylsilane (42 µL, 3 equiv.) was added, resulting in a colorless solution. The NMR tube was sealed and transferred out of the glove box. ^{1}H-,^{31}P{^{1}H}- and ^{1}H/^{31}P-HMBC were periodically measured. After approx. 30 min, the color of the solution changed to a bright yellow. After 24 h, full conversion of complex **1** could be detected. The reaction mixture was filtrated *via* a syringe filter into a Schlenk tube and all volatiles were removed, giving a sticky, colorless solid. The solid was three times washed with 2 mL *n*-pentane and dried *in vaccuo*, yielding 6 mg of **34** as colorless solid. [DIPPEMn(CO)$_3$H] (#) and an unknown silane-based byproduct (*) could not be separated from the title compound. Single crystal for X-ray analysis were obtained upon slow evaporation of a toluene solution.

^{1}H NMR (δ, 600 MHz, THF-*d8*, 20 °C): 1.45-0.94 (m, 48H), -8.72 (qq, J = 87.6 Hz, 10.5 Hz, 2H).^{13}C{^{1}H} NMR (δ, 151 MHz, THF-*d8*, 20 °C): 30.8 (vd), 30.2(vt), 29.9, 29.3(vd), 28.2(vt), 27.6(vt), 26.(vt), 22.7 19.6 (vd), 18.5(vm), CO not observed.^{31}P{^{1}H} NMR (δ, 243 MHz, THF-*d8*, 20 °C): 127.1-125.9 (m, 1P), 100.5-99.8 (m, 1P), 82.5-81.7 (m, 1P). ATR-IR (solid, cm^{-1}): 1889 (ν_{CO}), 1823 (ν_{CO}). HRMS (TOF ESI+): *m/z* calculated for $C_{46}H_{98}$Mn2O_4P_6 [M-2H]$^{+\bullet}$: 1010.4651, found 1010.4648.

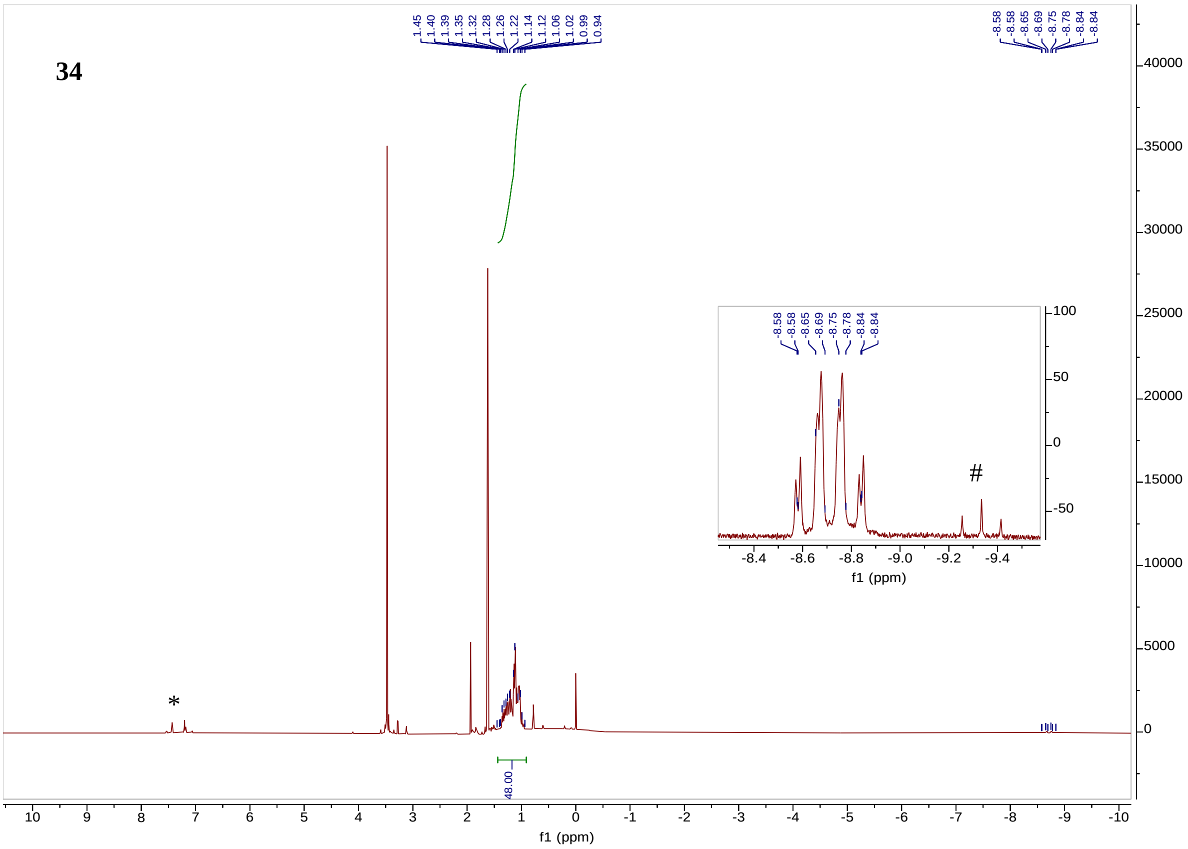

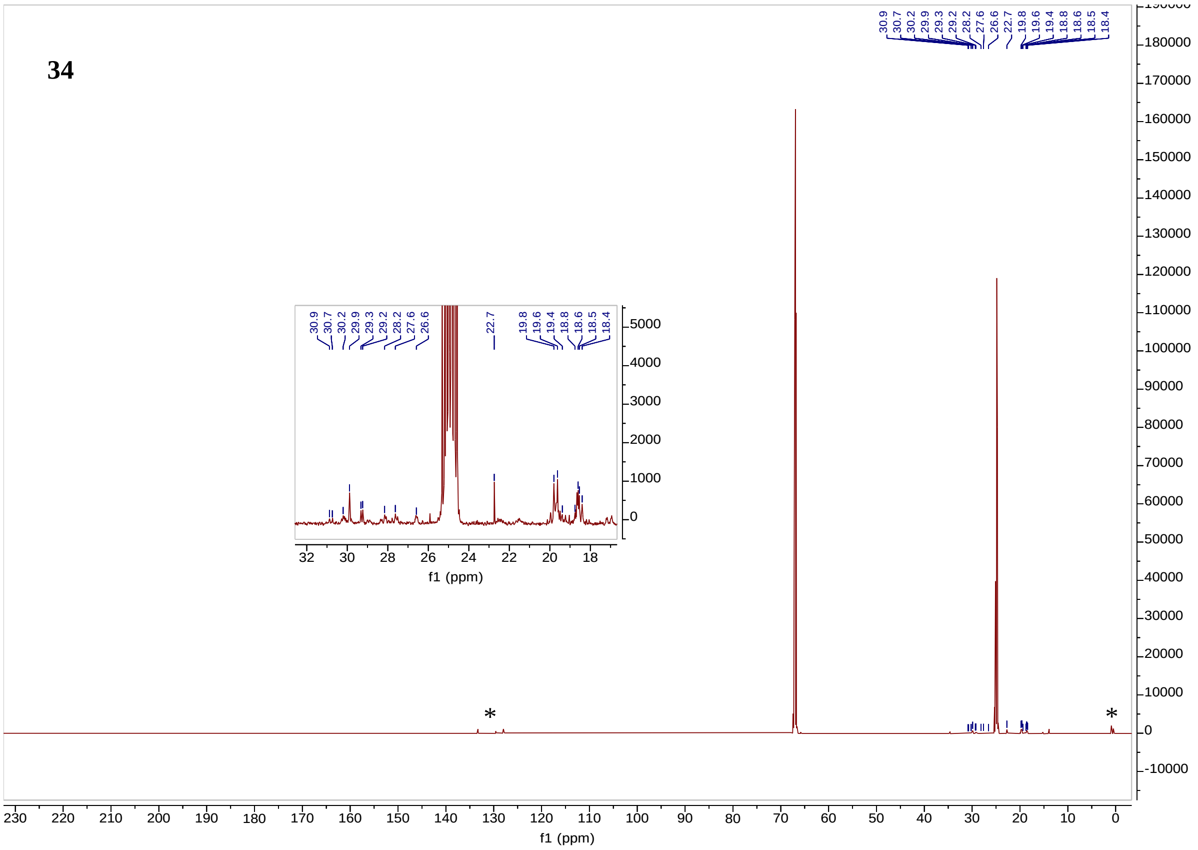
34
30.9
30.7
30.2
29.9
29.3
29.2
28.2
27.6
26.6
22.7
19.8
19.6
19.4
18.8
18.6
18.5
18.4
*
*
f1 (ppm)

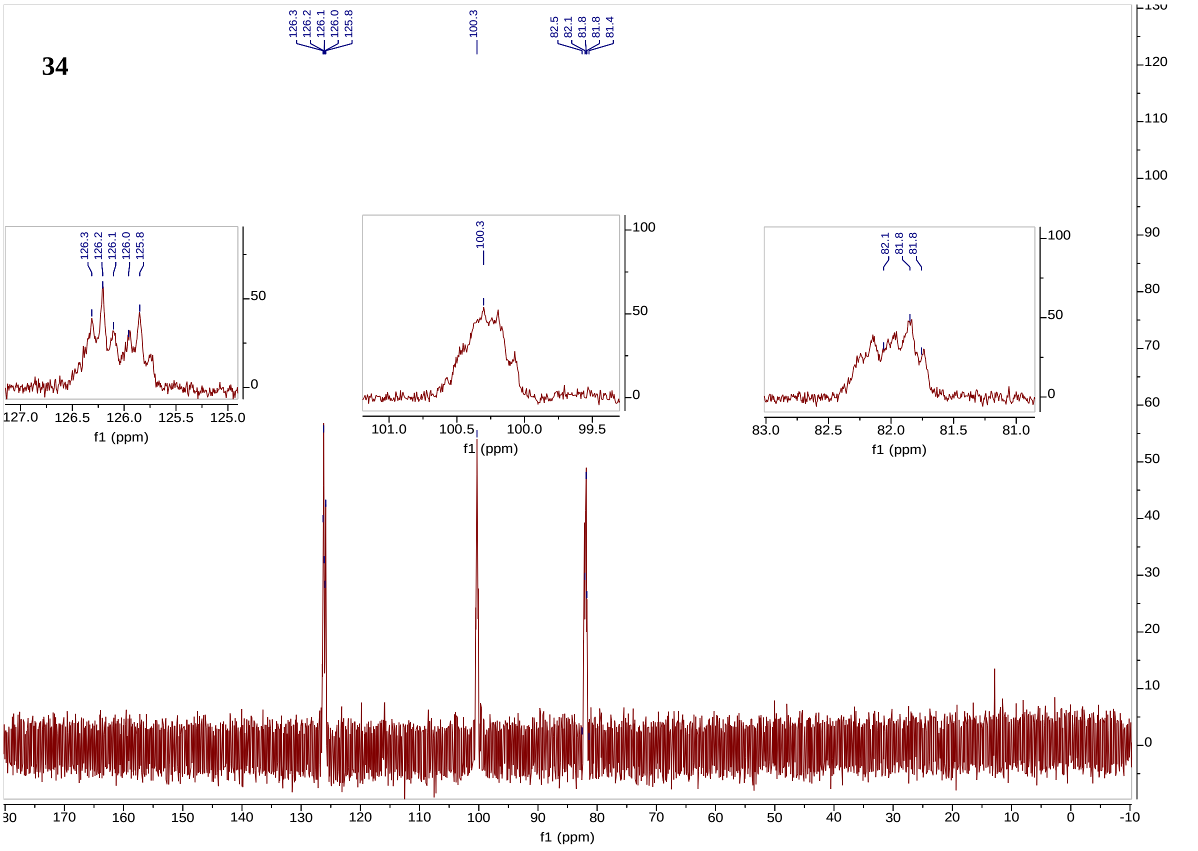
34
126.3
126.2
126.1
126.0
125.8
100.3
82.5
82.1
81.8
81.8
81.4
f1 (ppm)

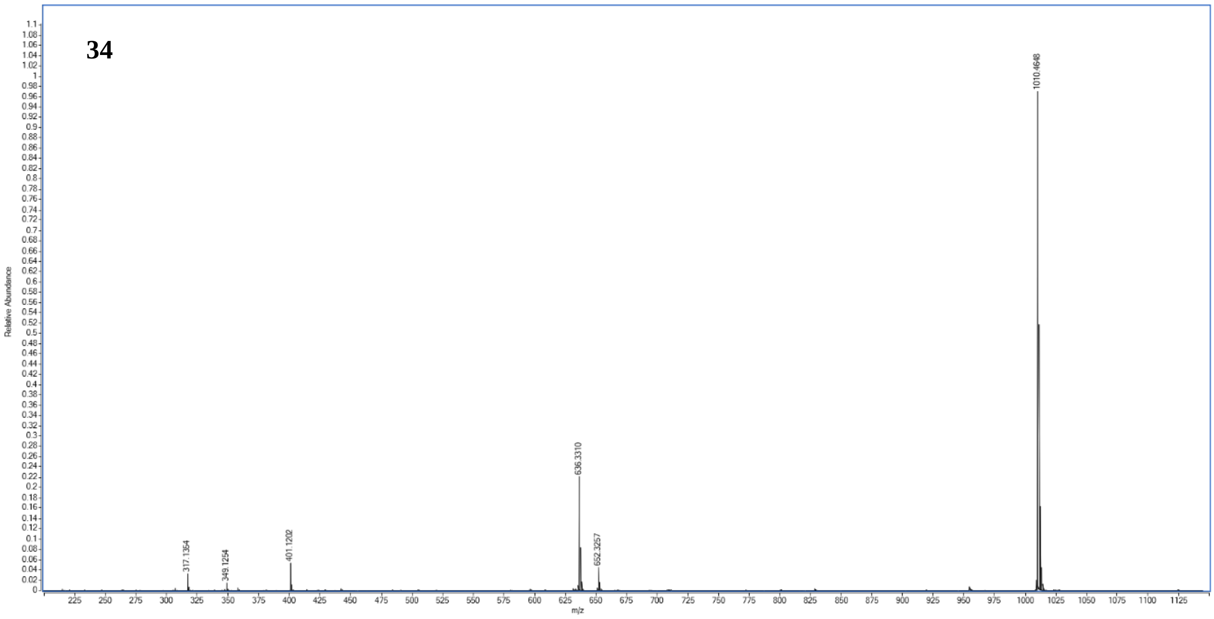
34
1010.4648
636.3310
652.3257
401.1202
317.1364
349.1254
Relative Abundance
m/z

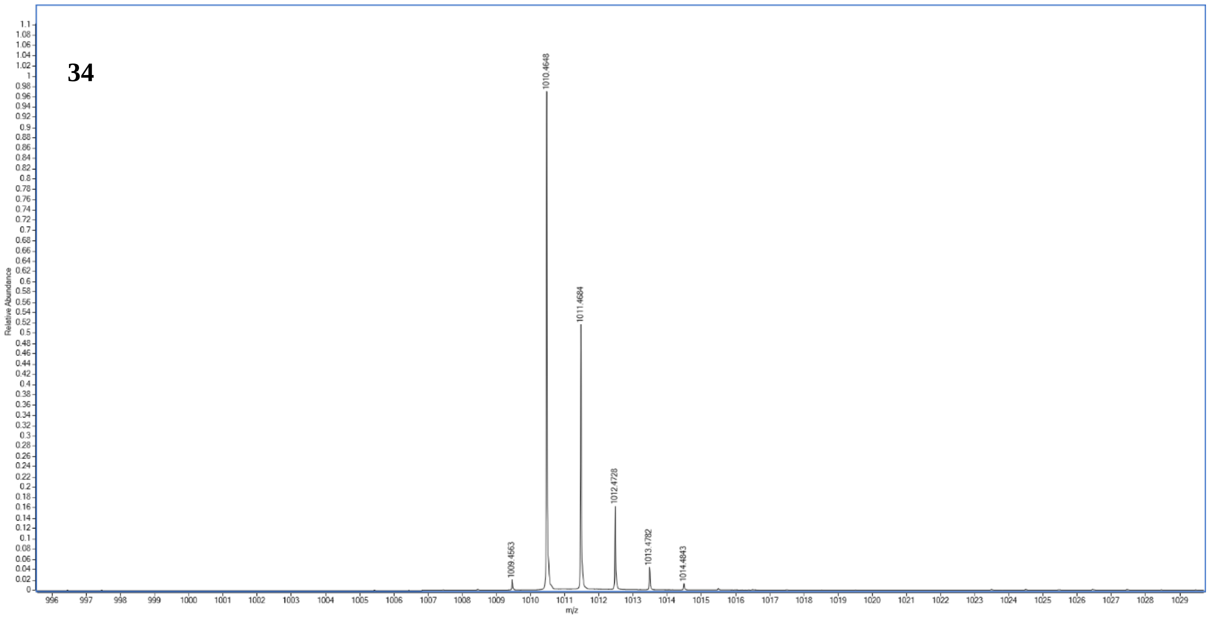
34
1010.4648
1011.4684
1012.4728
1013.4782
1014.4843
1009.4563
Relative Abundance
m/z

4.5 NMR analysis upon reaction progress

Inside an argon-flushed glove box complex **1** (5mg, 2 mol%), styrene (116 µL, 1.01 mmol, 1.8 equiv.) and phenyldimethylsilane (86.1 µL, 0.56 mmol, 1 equiv.) were mixed with 0.5 mL THF-*d8*, giving a colorless solution and transferred into an NMR tube. The NMR tube was sealed and transferred out of the glove box. ^{1}H- and $^{31}P\{^{1}H\}$-NMR were periodically measured. After approx. 10 min, the color of the solution changed to a bright yellow.

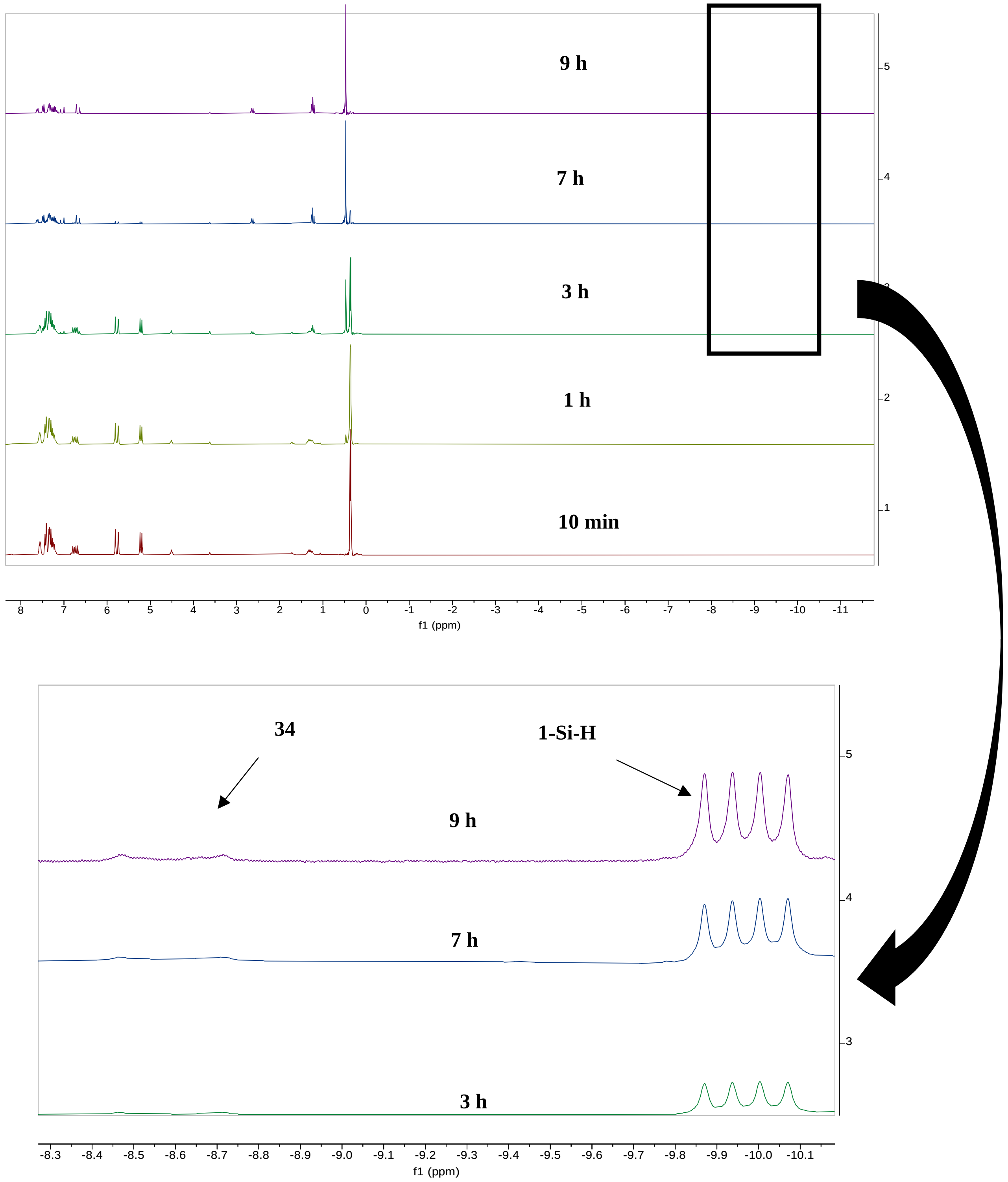

Figure S 10. ^{1}H-NMR during DS of Styrene with $HSiMe_2Ph$ and 2 mol% **1** in THF-*d8*.

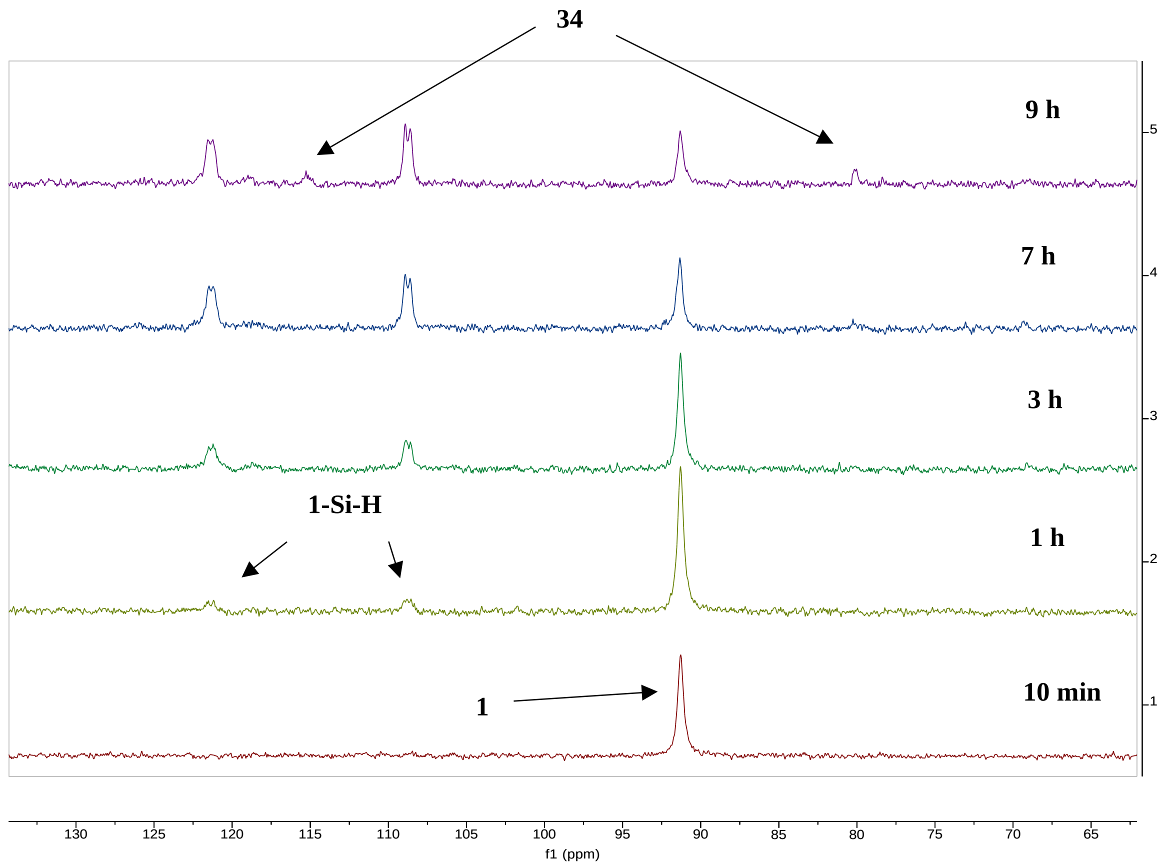

Figure S 11. $^{31}P\{^{1}H\}$-NMR during DS of Styrene with $HSiMe_2Ph$ and 2 mol% **1** in THF-*d8*.

ATR-IR analysis upon full conversion

Inside an argon-flushed glovebox, a screwcap-vial (8 mL) was charged with complex **1** (5mg, 2 mol%), styrene (116 µL, 1.01 mmol, 1.8 equiv.) and phenyldimethylsilane (86.1 µL, 0.56 mmol, 1 equiv.). A stirring-bar was added, the vial was sealed, transferred outside the glovebox and the reaction mixture was stirred for 24h. One drop of the neat reaction mixture was analyzed *via* ATR-IR.

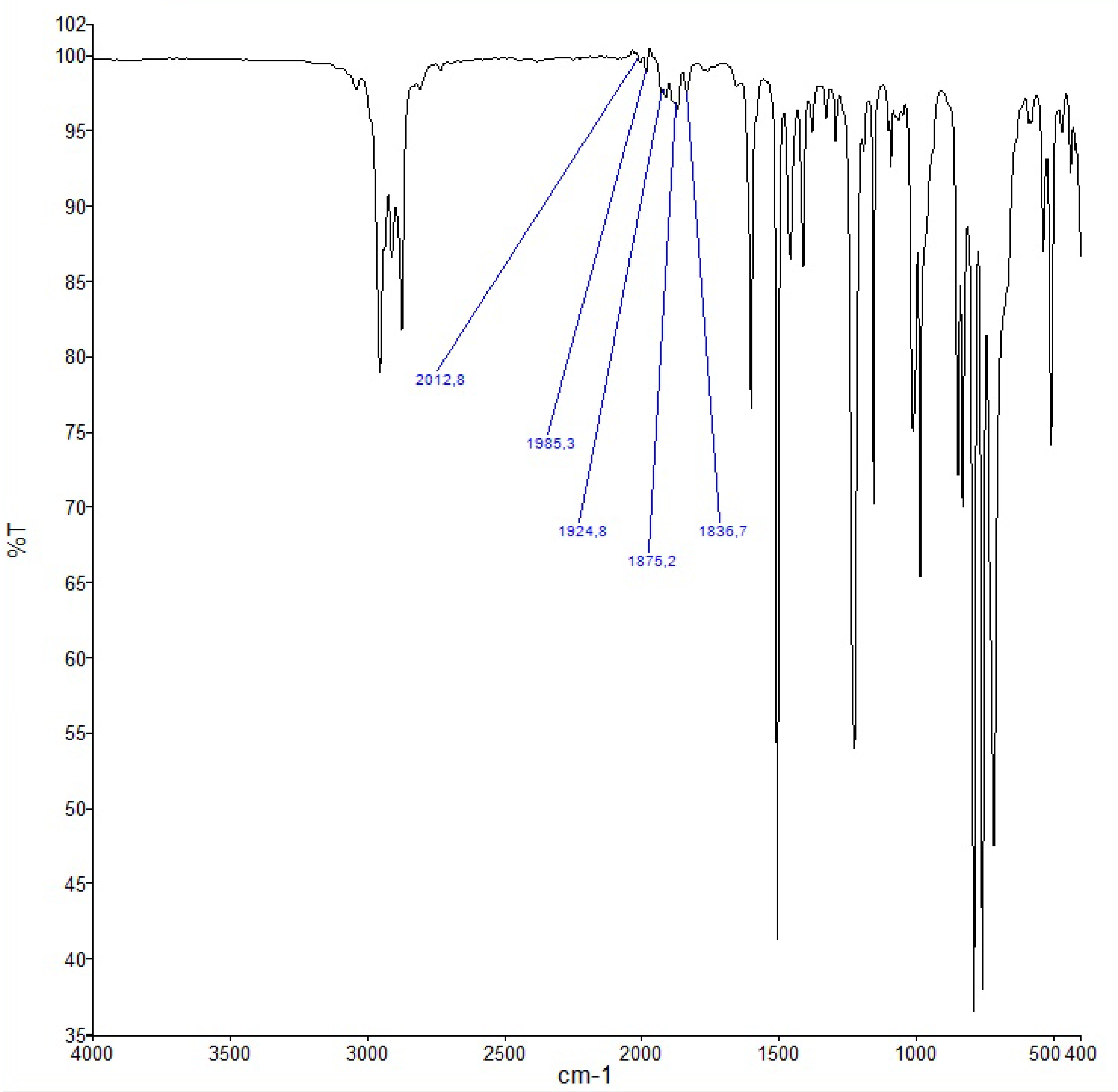

Figure S 12. ATR-IR analysis of catalytic reaction of **1** with styrene and $HSiMe_2Ph$

4.6 Investigation of *in-situ* *E*/*Z*-isomerization

Inside an argon-flushed glovebox, a screwcap-vial (8 mL) was charged with **1** (1.25 mg, 0.5 mol%), styrene (116 µL, 1.01 mmol, 1.8 equiv.), *Z*-dimethylphenyl(styryl)silane[16b] (139.6 mg, 0.56 mmol 1 equiv.) and phenyldimethylsilane (86.1 µL, 0.56 mmol, 1 equiv.) in this order. A stirring-bar was added, the vial was sealed with a cap and the mixture was stirred for 24 hours. A sample was taken and analyzed by ^{1}H-NMR recorded on a Bruker AVANCE-250 (250 MHz) in $CDCl_3$.

Note: If no isomerization occurs, the ratio of E/Z-isomer would be 50:50 rather than 70:30, as it is was observed.

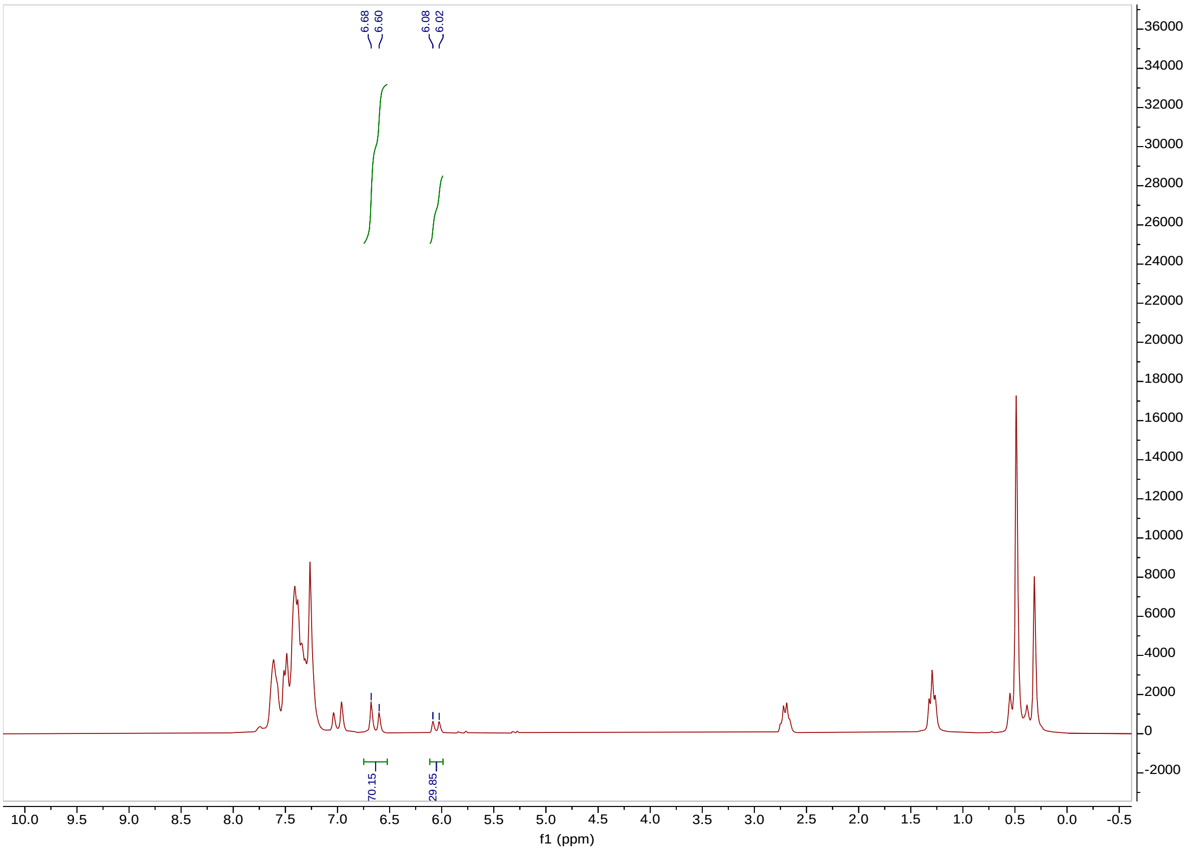

Figure S 13. Investigation of in-situ isomerization for the DS of Styrene with $HSiMe_2Ph$ catalyzed by **1**.

5 X-ray structure determination

X-ray diffraction data of **34** (CCDC 2094811) were collected at $T = 100$ K in a dry stream of nitrogen on a Bruker Kappa APEX II diffractometer system using graphite-monochromatized Mo-*K*α radiation (λ = 0.71073 Å) and fine sliced φ- and ω-scans. Data were reduced to intensity values with SAINT and an absorption correction was applied with the multi-scan approach implemented in SADABS.[17] The structure was solved by the dual-space approach implemented in SHELXT[18]and refined against F^2 with SHELXL.[19] Non-hydrogen atoms were refined with anisotropic displacement parameters. The H atoms connected to C atoms were placed in calculated positions and thereafter refined as riding on the parent atoms. The hydride atom was located from difference Fourier maps and refined freely. Molecular graphics were generated with the program MERCURY.[20]

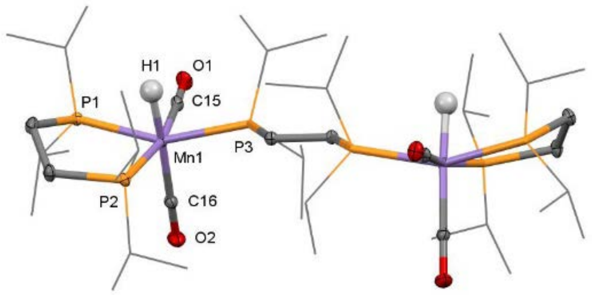

Figure S 14**.** Structural view of **34** showing 50% ellipsoids (most H atoms omitted for clarity). Selected bond distances (Å) and angles (°): Mn1-H1 1.51(2), Mn1-C15 1.770(5), Mn1-C16 1.793(3), Mn1-P1 2.232(7), Mn1-P2 2.328(7), Mn1-P3 2.309(3), C15-Mn1-P2 168.07(4), P1-Mn1-P2 85.16(2), P1-Mn1-H1 76.7(6), P2-Mn1-H1 76.7(6), P3-Mn1-H1 81.7(6)

6 Theoretical Calculations

DFT Calculations: The computational results presented have been achieved in part using the Vienna Scientific Cluster (VSC). All calculations were performed using the GAUSSIAN 09 software package.[21] Geometry optimizations were obtained using the PBE0 functional without symmetry constraints and a basis set (b1) consisting of the Stuttgart/Dresden ECP (SDD) basis set[22] to describe the electrons of Mn, and a standard 6-31G(d,p) basis set[23] for all other atoms. The PBE0 functional uses a hybrid generalized gradient approximation (GGA), including 25 % mixture of Hartree-Fock[24] exchange with DFT[25] exchange-correlation, given by Perdew, Burke and Ernzerhof functional (PBE).[26] Frequency calculations were performed to confirm the nature of the stationary points as minima, yielding no imaginary frequencies. The electronic energies (E_{b1}) were converted to free energy at 298.15 K and 1 atm (G_{b1}) by using zero point energy and thermal energy corrections based on structural and vibration frequency data calculated at the same level. To evaluate the effect of the hydrogen partial pressure on the free energy profile we reduced the partial pressure from 1.0 to 10^{-4}, 10^{-6}, and 10^{-8} atm, respectively.^{1}H chemical sifts were calculated at the same level of theory for the optimized structure of [Mn(dippe)$(CO)_2$(η^1-C(O)$CH_2CH_2CH_3$)(η^1-H-$SiMe_2$Ph)] (**1-Si-H**) using the gauge-independent atomic orbital (GIAO) method in Gaussian 09 with the above basis sets. Chemical shifts are given with respect to $SiMe_4$ at the same computational level.

Single point energy calculations were performed on the geometries obtained at the PBE0/b1 level using the M06 functional, and a 6-311++G(d,p) basis set[27] for the rest of the elements. The M06 functional is a hybrid meta-GGA functional developed by Truhlar and Zhao,[28] and it was shown to perform very well for the kinetics of transition

metal molecules, providing a good description of weak and long range interactions.[29] The free energy values presented (G_{b2}) were derived from the electronic energy values obtained at the M06/6-311++G(d,p)//PBE0/b1 level, including solvent effects (E_{b2}), according to the following expression: $G_{b2} = E_{b2} + G_{b1} – E_{b1}$.

A natural population analysis (NPA)[30] and the resulting Wiberg indices[31] were used to study the electronic structure and bonding of the optimized species. The NPA analysis was performed with the NBO 5.0 program.[32]

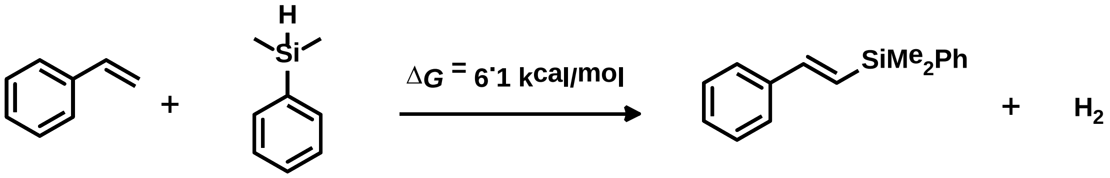

Equation S 1. Thermodynamic data of the DS of styrene with $HSiMe_2Ph$

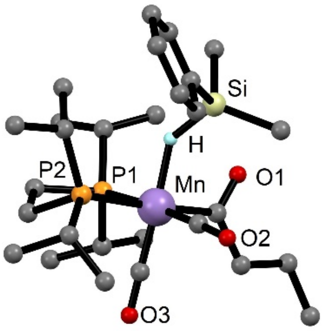

Figure S 15. Optimized geometry (PBE0/b1) for **1-Si-H** (most H atoms omitted for clarity).

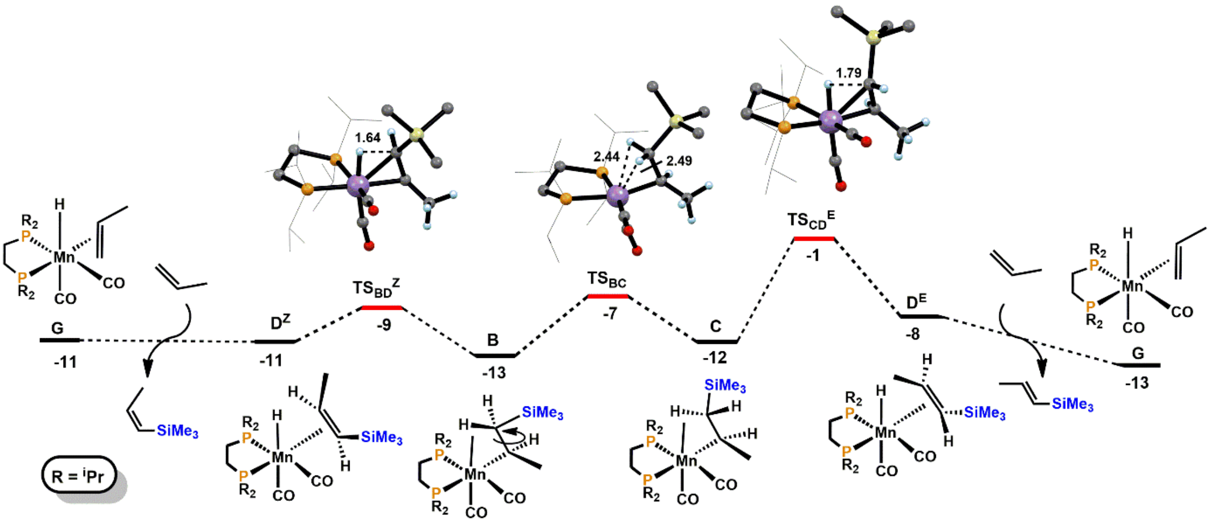

Figure S 16. Free Energy Profile Calculated at M06/(6-311++G**)//PBE0/(SDD,6-31G**) Level for the *Z* to *E* Isomerization of Vinylsilenes and Product Release upon Addition of Propene. Free Energies (kcal/mol) are Referred to **A**.

7 References

(1) Weber, S.; Stöger, B.; Veiros, L.F.; Kirchner, K. Rethinking Basic Concepts - Hydrogenation of Alkenes Catalyzed by Bench-Stable Alkyl Mn(I) Complexes. *ACS Catal.* **2019**, *9*, 9715-9720.

(2) Garduño, J. A.; Garcia, J. J. Non-Pincer Mn(I) Organometallics for the Selective Catalytic Hydrogenation of Nitriles to Primary Amines ACS Catal. **2018**, *8*, 392-401.

(3) Gu, J.; Cai, C. Stereoselective synthesis of vinylsilanes *via* copper-catalyzed silylation of alkenes with silanes. *Chem. Comm.* **2016**, *52*, 10779-10782.

(4) Duan, Y.; Ji, G.; Zhang, S.; Chen, X.; Yang, Y. Additive-modulated switchable reaction pathway in the addition of alkynes with organosilanes catalyzed by supported Pd nanoparticles: hydrosilylation versus semihydrogenation. *Catal. Sci. Technol.* **2018**, *8*, 1039-1050.

(5) Bokka, A.; Jeon, J. Regio- and Stereoselective Dehydrogenative Silylation and Hydrosilylation of Vinylarenes Catalyzed by Ruthenium Alkylidenes. *Org. Lett.* **2016**, *18*, 5324-5327.

(6) Tyagi, A.; Yadav, S.; Daw, P.; Ravi, C.; Bera, J.K. A Rh(I) complex with an annulated N-heterocyclic carbene ligand for E-selective alkyne hydrosilylation. *Polyhedron* **2019**, *172*, 167-174.

(7) Berthon-Gelloz, G.; Schumers, J.-M.; De Bo, G.; Marko, I. E. Visible light accelerated hydrosilylation of alkynes using platinum–[acyclic diaminocarbene] photocatalysts. *J. Org. Chem.* **2008**, *73*, 4190–4197.

(8) Tyagi, A.; Yadav, S.; Daw, P.; Ravi, C.; Bera, J. K. A Rh(I) complex with an annulated N-heterocyclic carbene ligand for E-selective alkyne hydrosilylation. *Polyhedron* **2019**, *172*, 167-174.

(9) Marciniec, B.; Majchrzak, M.; Prukala, W.; Kubicki, M.; Chadyniak, D. Highly Stereoselective Synthesis, Structure, and Application of (*E*)-9-[2-(Silyl)ethenyl]-9*H*-carbazoles. *J. Org. Chem.* **2005**, *70*, 8550-8555.

(10) Gan, Y.; Xu, W.; Liu, Y. Ligand-Controlled Regiodivergent Silylation of Allylic Alcohols by Ni/Cu Catalysis for the Synthesis of Functionalized Allylsilanes. *Org. Lett.* **2019**, *19*, 9652-9657.

(11) Yang, B.; Wang, Z.-X. Synthesis of Allylsilanes via Nickel-Catalyzed Cross-Coupling of Silicon Nucleophiles with Allyl Alcohols. *Org. Lett.*, **2019**, *19*, 7965-7969.

(12) Ollivier, J.; Salaün, J. Synthesis of Allylsilanes by Palladium(0) Catalyzed Reduction of Trialkylsilylallyl Esters. *Synlett* **1994**, *11*,949-951.

(13) Oestreich, M.; Auer, G. Practical Synthesis of Allylic Silanes from Allylic Esters and Carbamates by Stereoselective Copper-Catalyzed Allylic Substitution Reactions. *Adv. Syn. Catal.* **2005**, *347*, 637-640.

(14) Atienza, C. C. H.; Diao, T.; Weller, K.J.; Nye, S.A., Lewis, K.M, Delis, J.G.P., Boyer, J.L. Roy, A.K; Chirik, P.J. Bis(imino)pyridine Cobalt-Catalyzed Dehydrogenative Silylation of Alkenes: Scope, Mechanism, and Origins of Selective Allylsilane Formation. *J. Am. Chem. Soc.* **2014**, *136*, 12108-12118.

(15) Cassani, M. C.; Brucka, M. A.; Femoni, C.; Mancilli, M.; Mazzanti, A.; Mazzoni, R.; Solinas, G. N-Heterocyclic carbene rhodium(I) complexes containing an axis of chirality: dynamics and catalysis. *New J. Chem.* **2014**, *38*, 1768 – 1779.

(16) (a) Wissing, M., Studer A. Tuning the Selectivity of AuPd Nanoalloys towards Selective Dehydrogenative Alkyne Silylation. *Chem. Eur. J*, **2019**, *25*, 5870-5874. (b) S. V. Maifeld, M. N. Tran, D. Lee. Hydrosilylation of alkynes catalyzed by ruthenium carbene complexes. *Tetrahydron Lett.* **2005**, *46*, 105-108.

(17) Bruker computer programs: APEX3, SAINT and SADABS (Bruker AXS Inc., Madison, WI, 2020).

(18) Sheldrick, G. M. *Acta Crystallogr.* **2015**, *A71*, 3–8.

(19) Sheldrick, G. M. *Acta Crystallogr.* **2015**, *C71*, 3–8.

(20) Macrae, C. F.; Edgington, P. R.; McCabe, P.; Pidcock, E.; Shields, G. P.; Taylor, R.; Towler, M.; van de Streek, J. *J. Appl. Cryst.* **2006**, *39*, 453–457.

(21) Gaussian 09, Revision **A.01**, Frisch, M. J.; Trucks, G. W.; Schlegel, H. B.; Scuseria, G. E.; Robb, M. A.; Cheeseman, J. R.; Scalmani, G.; Barone, V.; Mennucci, B.; Petersson, G. A.; Nakatsuji, H.; Caricato, M.; Li, X.; Hratchian, H. P.; Izmaylov, A. F.; Bloino, J.; Zheng, G.; Sonnenberg, J. L.; Hada, M.; Ehara, M.; Toyota, K.; Fukuda, R.; Hasegawa, J.; Ishida, M.; Nakajima, T.; Honda, Y.; Kitao, O.; Nakai, H.; Vreven, T.; Montgomery, Jr., J. A.; Peralta, J. E.; Ogliaro, F.; Bearpark, M.; Heyd, J. J.; Brothers, E.; Kudin, K. N.; Staroverov, V. N.; Kobayashi, R.; Normand, J.; Raghavachari, K.; Rendell, A.; Burant, J. C.; Iyengar, S. S.; Tomasi, J.; Cossi, M.; Rega, N.; Millam, J. M.; Klene, M.; Knox, J. E.; Cross, J. B.; Bakken, V.; Adamo, C.; Jaramillo, J.; Gomperts, R.; Stratmann, R. E.; Yazyev, O.; Austin, A. J.; Cammi, R.; Pomelli, C.; Ochterski, J. W.; Martin, R. L.; Morokuma, K.; Zakrzewski, V. G.; Voth, G. A.; Salvador, P.; Dannenberg, J. J.; Dapprich, S.; Daniels, A. D.; Farkas, Ö.; Foresman, J. B.; Ortiz, J. V.; Cioslowski, J.; Fox, D. J. Gaussian, Inc., Wallingford CT, 2009.

(22) (a) Haeusermann, U.; Dolg, M.; Stoll, H.; Preuss, H.; Schwerdtfeger, P.; Pitzer, R. M. Accuracy of energy-adjusted quasirelativistic ab initio pseudopotentials *Mol. Phys.* **1993**, *78*, 1211-1224. (b) Kuechle, W.; Dolg, M.; Stoll, H.; Preuss, H. Energy-adjusted pseudopotentials for the actinides. Parameter sets and test calculations for thorium and thorium monoxide *J. Chem. Phys.* **1994**, *100*, 7535-7542. (c) Leininger, T.; Nicklass, A.; Stoll, H.; Dolg, M.; Schwerdtfeger, P. The accuracy of the pseudopotential approximation. II. A comparison of various core sizes for indium pseudopotentials in calculations for spectroscopic constants of InH, InF, and InCl *J. Chem. Phys.* **1996**, *105*, 1052-1059.

(23) (a) Ditchfield, R.; Hehre, W. J.; Pople, J. A. Self-Consistent Molecular-Orbital Methods. IX. An Extended Gaussian-Type Basis for Molecular-Orbital Studies of Organic Molecules *J. Chem. Phys.* **1971**, *54*, 724-728. (b) Hehre, W. J.; Ditchfield, R.; Pople, J. A. Self-Consistent Molecular Orbital Methods. 12. Further extensions of Gaussian-type basis sets for use in molecular-orbital studies of organic-molecules *J. Chem. Phys.* **1972**, *56*, 2257-2261. (c) Hariharan, P. C.; Pople, J. A. Accuracy of AH equilibrium geometries by single determinant molecular-orbital theory *Mol. Phys.* **1974**, *27*, 209-214. (d) Gordon, M. S. The isomers of silacyclopropane *Chem. Phys. Lett.* **1980**, *76*, 163-168. (e) Hariharan, P. C.; Pople, J. A. Influence of polarization functions on molecular-orbital hydrogenation energies *Theor. Chim. Acta* **1973**, *28*, 213-222.

(24) Hehre, W. J., Radom, L., Schleyer, P. v.R. & Pople, J. A. *Ab Initio Molecular Orbital Theory*, John Wiley & Sons, NY, 1986.

(25) Parr, R. G. & Yang, W. *Density Functional Theory of Atoms and Molecules*; Oxford University Press: New York, 1989.

(26) (a) Perdew, J. P.; Burke, K.; Ernzerhof, M. Generalized Gradient Approximation Made Simple *Phys. Rev. Lett.* **1996**, *77*, 3865-3868; (b) Perdew, J. P.; Burke, K.; Ernzerhof, M. Generalized Gradient Approximation Made Simple *Phys. Rev. Lett.* **1997**, *78*, 1396-1396. (c) Perdew, J. P. Density-functional approximation for the correlation energy of the inhomogeneous electron gas *Phys. Rev. B* **1986**, *33*, 8822-8824.

(27)(a) McClean, A. D.; Chandler, G. S. Contracted Gaussian basis sets for molecular calculations. I. Second row atoms, *Z*=11-18 *J. Chem. Phys.* **1980**, *72*, 5639-5648. (b) Krishnan, R.; Binkley, J. S.; Seeger, R.; Pople, J. A. Self-consistent molecular orbital methods. XX. A basis set for correlated wave functions *J. Chem. Phys.* **1980**, *72*, 650-654. (c) Wachters, A. J. H. Gaussian Basis Set for Molecular Wavefunctions Containing Third-Row Atoms *J. Chem. Phys.* **1970**, *52*, 1033-1036. (d) Hay, P. J. Gaussian basis sets for molecular calculations - representation of 3D orbitals in transition-metal atoms *J. Chem. Phys.* **1977**, *66*, 4377-4384. (e) Raghavachari, K.; Trucks, G. W. Highly correlated systems: Excitation energies of first row transition metals Sc-Cu *J. Chem. Phys.* **1989**, *91*, 1062-1065. (f) Binning Jr., R. C.; Curtiss, L. A. Compact contracted basis-sets for 3rd-row atoms - Ga-Kr *J. Comp. Chem.* **1990**, *11*, 1206-1216. (g) McGrath, M. P.; Radom, L. Extension of Gaussian-1 (G1) theory to bromine-containing molecules *J. Chem. Phys.* **1991**, *94*, 511-516. (h) Curtiss, L. A.; McGrath, M. P.; Blaudeau, J.-P.; Davis, N. E.; Binning Jr., R. C.; Radom, L. Extension of Gaussian-2 theory to molecules containing third-row atoms Ga-Kr *J. Chem. Phys.*, **1995**, *103*, 6104-6113. (i) Clark, T.; Chandrasekhar, J.; Spitznagel, G. W.; Schleyer, P. v. R. Efficient diffuse function-augmented basis-sets for anion calculations. 3. The 3-21+G basis set for 1st-row elements, Li-F *J. Comp. Chem.* **1983**, *4*, 294-301. (j) Frisch, M. J.; Pople, J. A.; Binkley, J. S. Self-Consistent Molecular Orbital Methods. 25. Supplementary Functions for Gaussian Basis Sets *J. Chem. Phys.* **1984**, *80*, 3265-3269.

(28) Zhao, Y.; Truhlar, D. G. The M06 suite of density functionals for main group thermochemistry, thermochemical kinetics, noncovalent interactions, excited states, and transition elements: two new functionals and systematic testing of four M06-class functionals and 12 other functionals *Theor. Chem. Acc.*, **2008**, *120*, 215-241.

(29) (a) Zhao, Y.; Truhlar, D. G. Density Functionals with Broad Applicability in Chemistry *Acc. Chem. Res.* **2008**, *41*, 157-167. (b) Zhao, Y.; Truhlar, D. G. Applications and validations of the Minnesota density functionals *Chem. Phys. Lett.* **2011**, *502*, 1-13.

(30) (a) Carpenter, J. E.; Weinhold, F. Analysis of the geometry of the hydroxymethyl radical by the “different hybrids for different spins” natural bond orbital procedure *J. Mol. Struct. (Theochem)*, **1988,** *169*, 41-62. (b) Carpenter, J. E. Extension of Lewis structure concepts to open-shell and excited-state molecular species *PhD. Thesis*. University of Wisconsin, Madison, WI, **1987**. (c) Foster, J. P.; Weinhold, F. Natural hybrid orbitals *J. Am. Chem. Soc.*, **1980,** *102*, 7211-7218. (d) Reed, A. E.; Weinhold, F. Natural bond orbital analysis of near-Hartree-Fock water dimer *J. Chem. Phys.*, **1983,** *78*, 4066-4073. (e) Reed, A. E.; Weinhold, F. Natural localized molecular orbitals *J. Chem. Phys.*, **1985,** *83*, 1736-1740. (f) Reed, A. E.; Weinstock, R. B.; Weinhold, F. Natural population analysis *J. Chem. Phys.*, **1985,** *83*, 735-746. (g) Reed, A. E.; Curtiss, L. A.; Weinhold, F. Intermolecular interactions from a natural bond orbital, donor-acceptor viewpoint *Chem. Rev.*, **1988,** *88*, 899-

926. (h) Weinhold, F.; Carpenter, J. E. *The Structure of Small Molecules and Ions*. Plenum, New York, **1988**; p 227.

(31) (a) Wiberg, K. B. Application of the pople-santry-segal CNDO method to the cyclopropylcarbinyl and cyclobutyl cation and to bicyclobutane *Tetrahedron* **1968**, *24*, 1083-1096. (b) Wiberg indices are electronic parameters related with the electron density in between two atoms, which scale as bond strength indicators. They can be obtained from a Natural Population Analysis.

(32) *NBO 5.0*. Glendening, E. D.; Badenhoop, J. K.; Reed, A. E.; Carpenter, J. E.; Bohmann, J. A.; Morales, C. M.; Weinhold, F.; Theoretical Chemistry Institute, University of Wisconsin, Madison, 2001.

8 NMR Spectra of all Organic Products

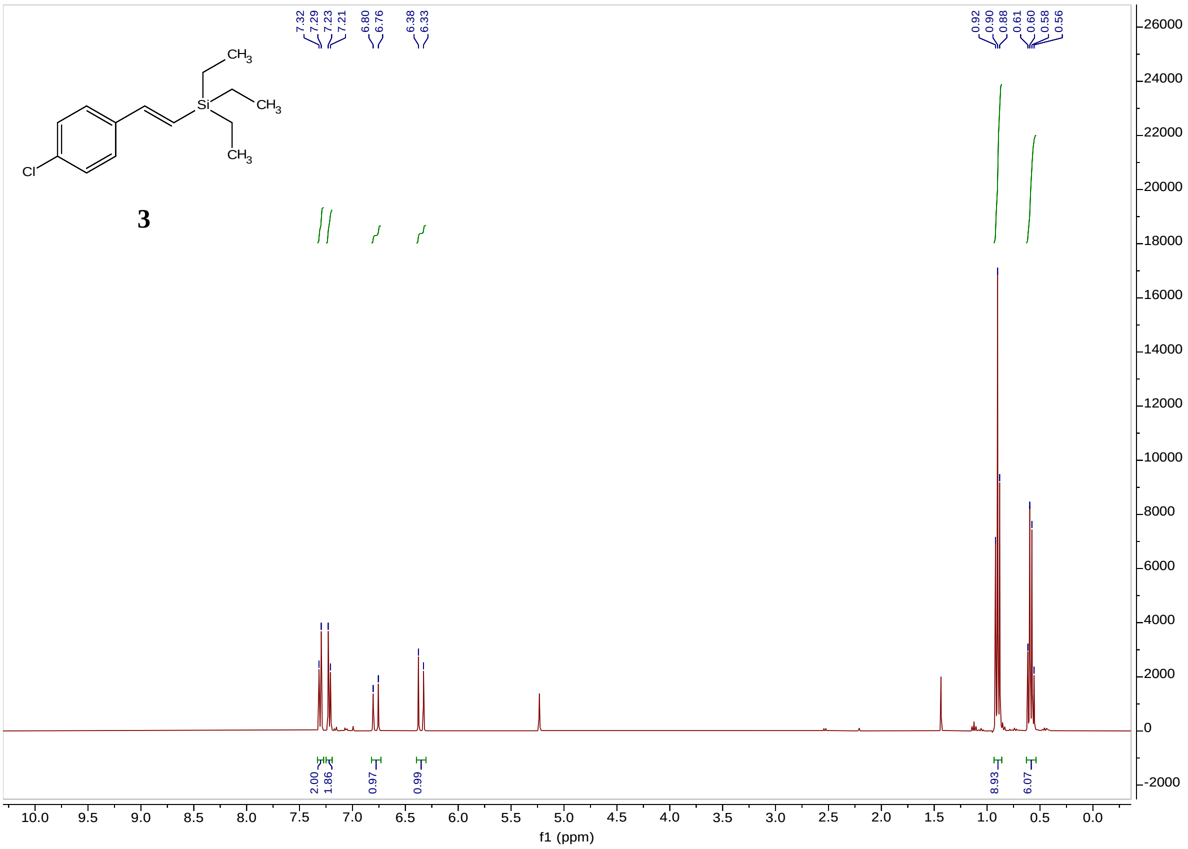

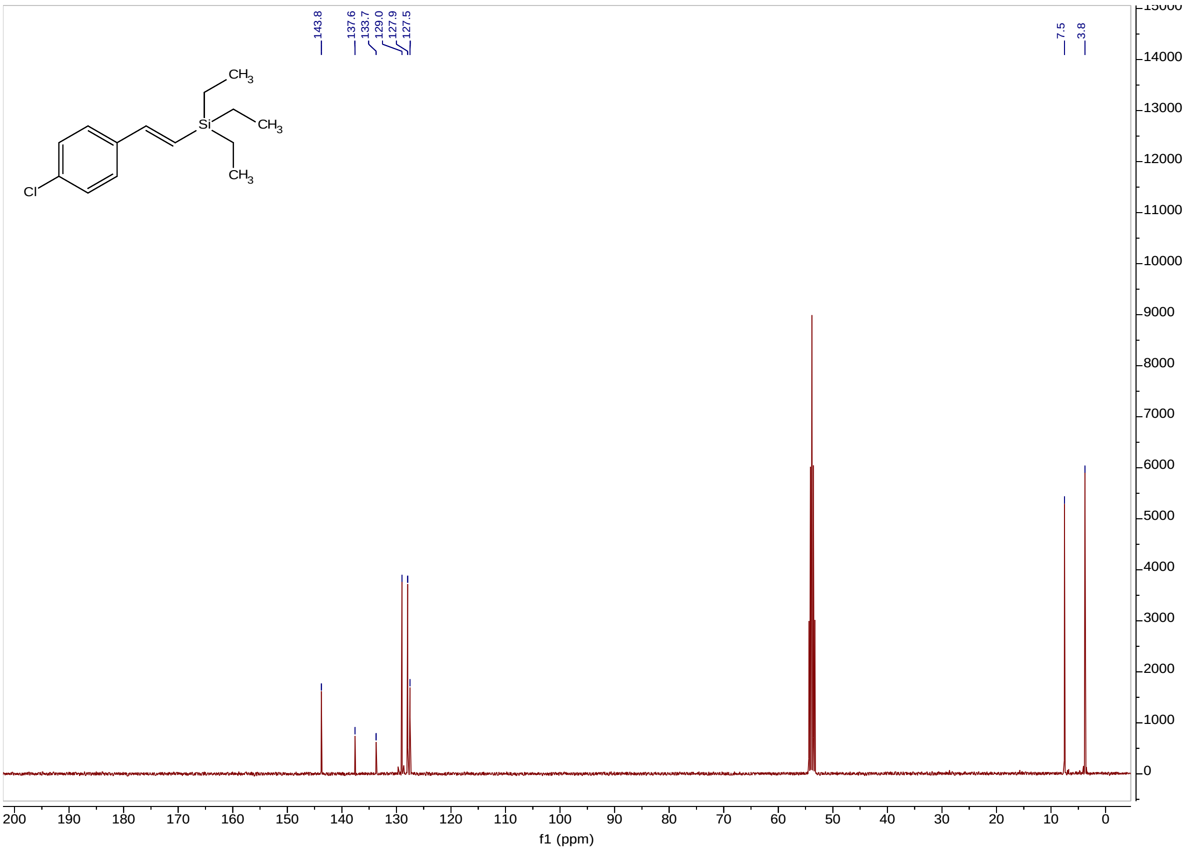

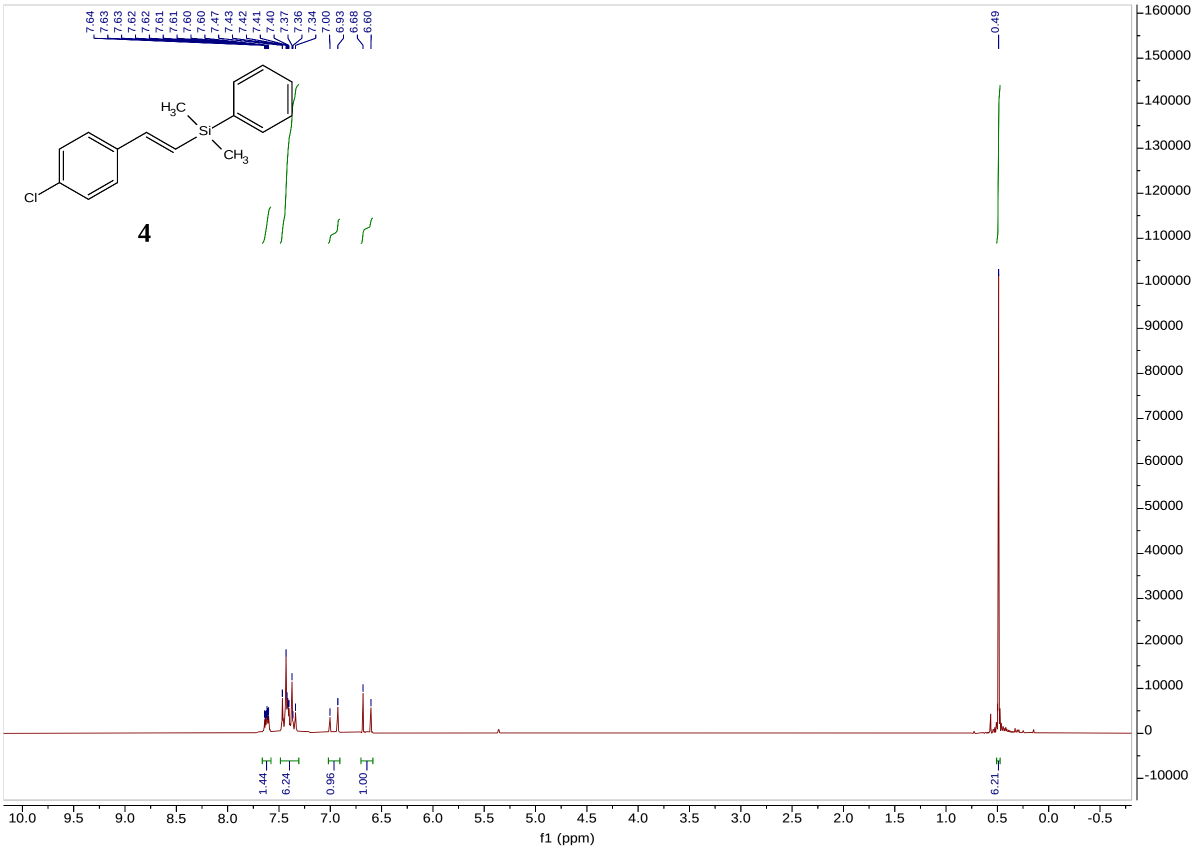
4
f1 (ppm)

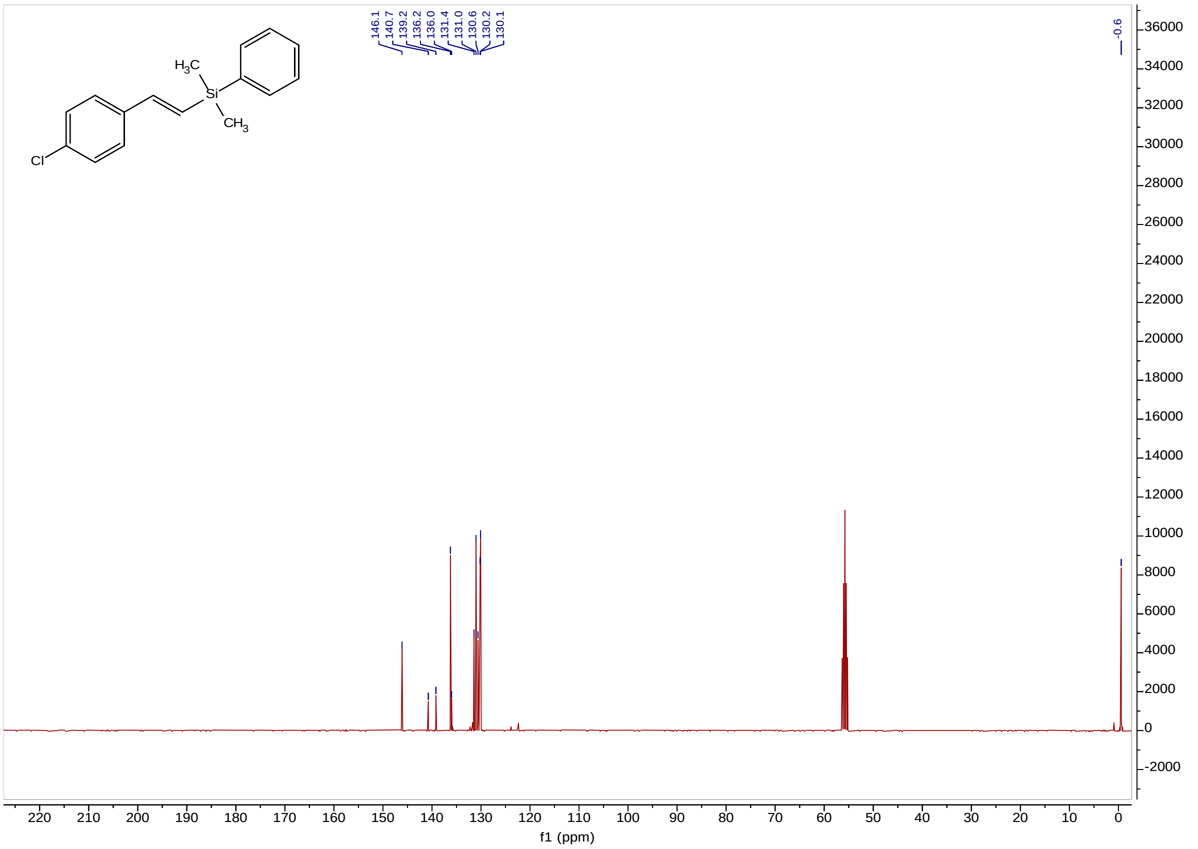
f1 (ppm)

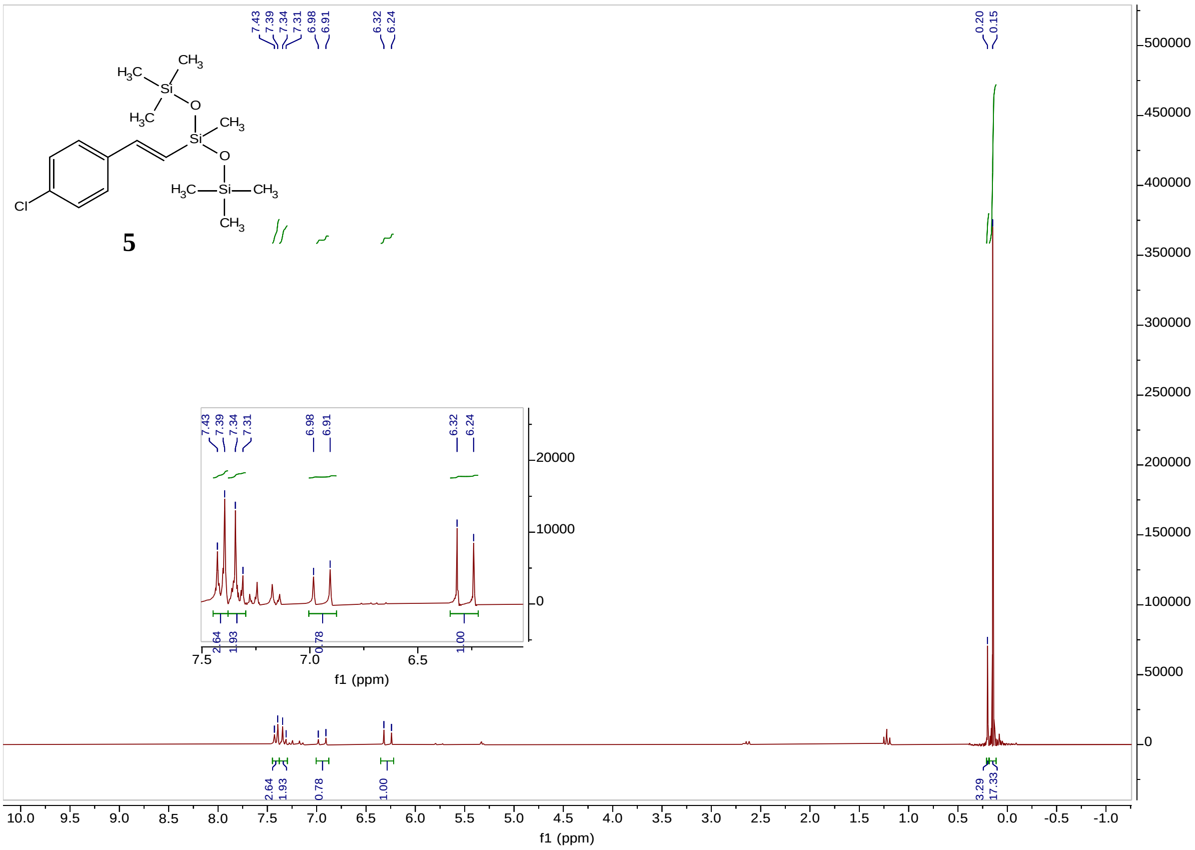
7.43
7.39
7.34
7.31
6.98
6.91
6.32
6.24
0.20
0.15
5
2.64
1.93
0.78
1.00
3.29
17.33
f1 (ppm)

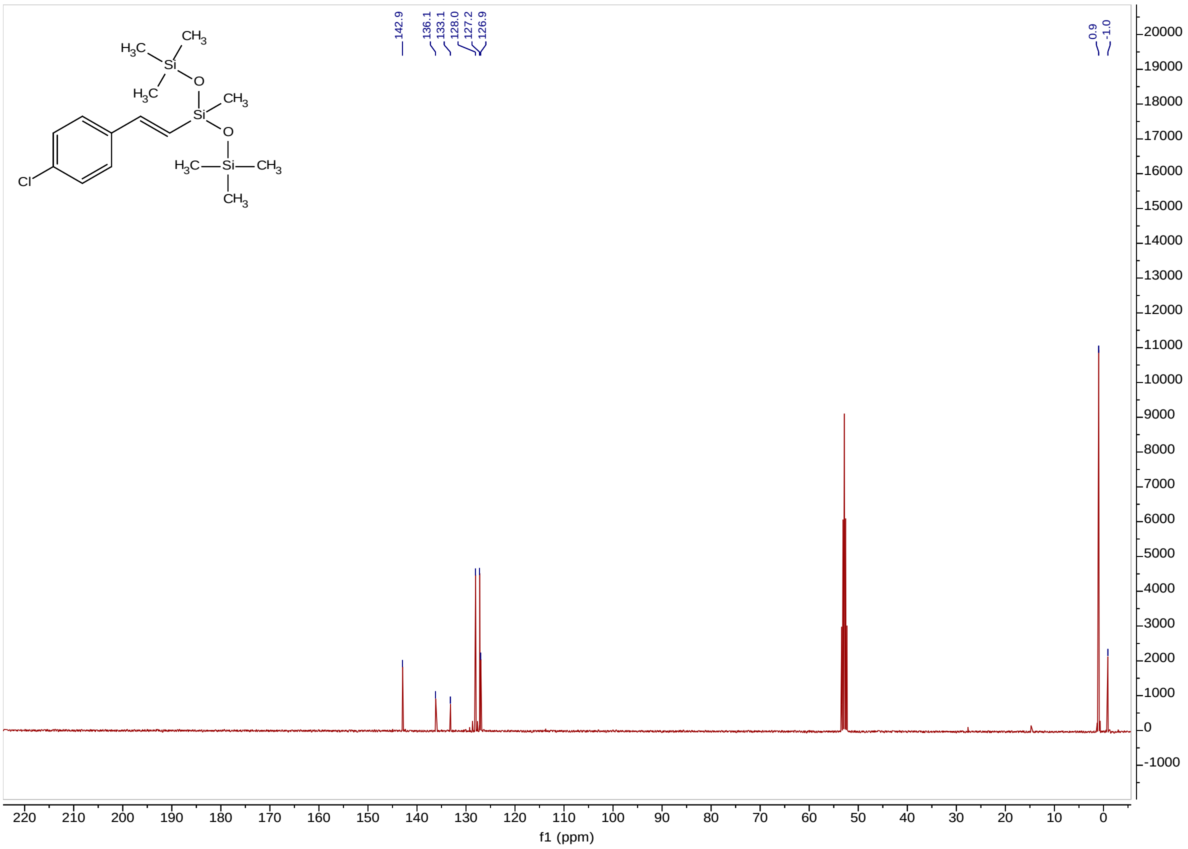
142.9
136.1
133.1
128.0
127.2
126.9
0.9
-1.0
f1 (ppm)

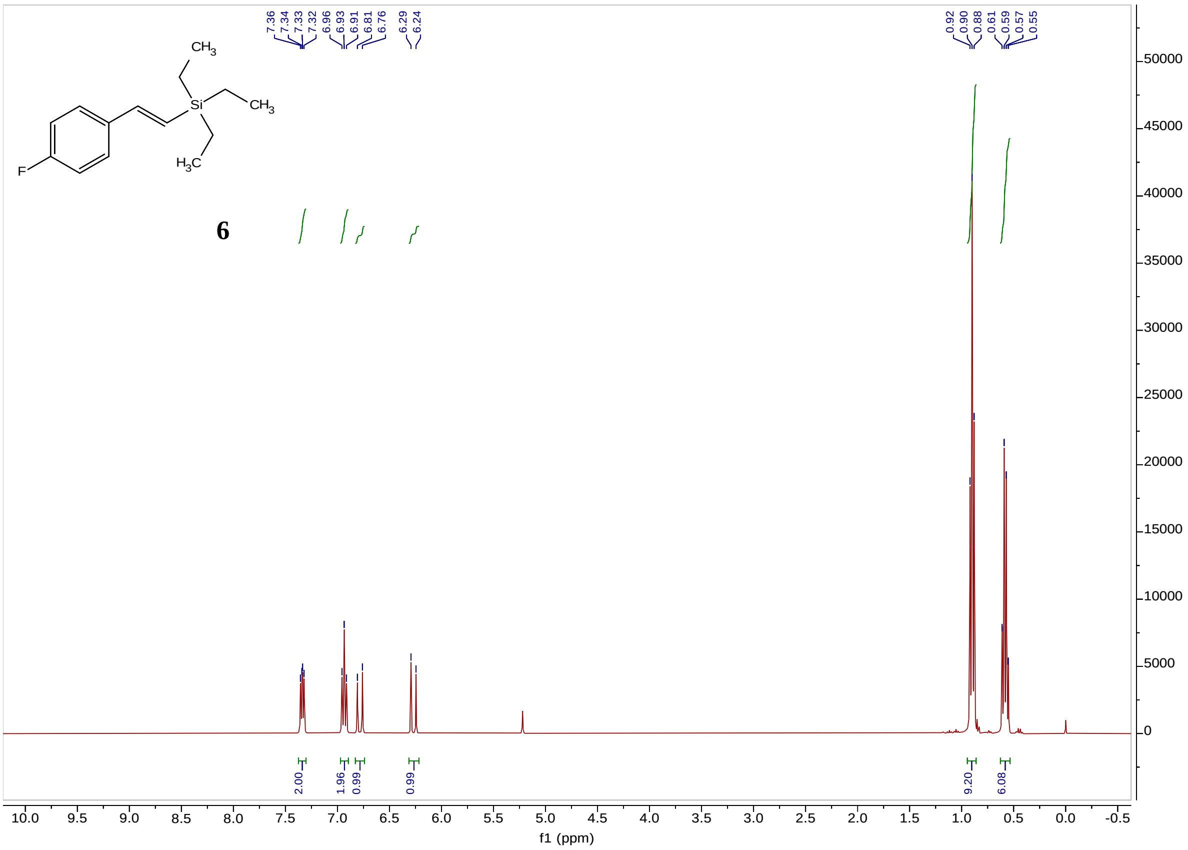
6
7.36
7.34
7.33
7.32
6.96
6.93
6.91
6.81
6.76
6.29
6.24
0.92
0.90
0.88
0.61
0.59
0.57
0.55
2.00
1.96
0.99
0.99
9.20
6.08
f1 (ppm)

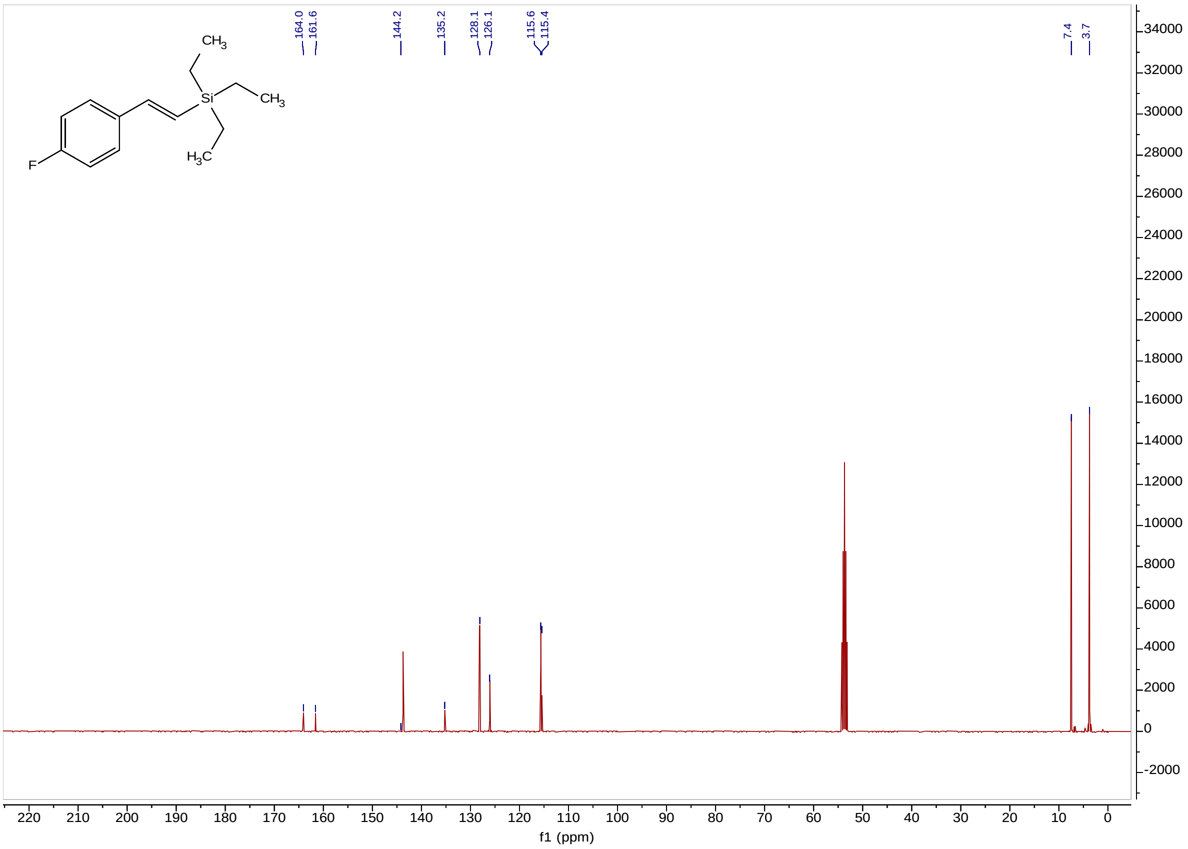
164.0
161.6
144.2
135.2
128.1
126.1
115.6
115.4
7.4
3.7
f1 (ppm)

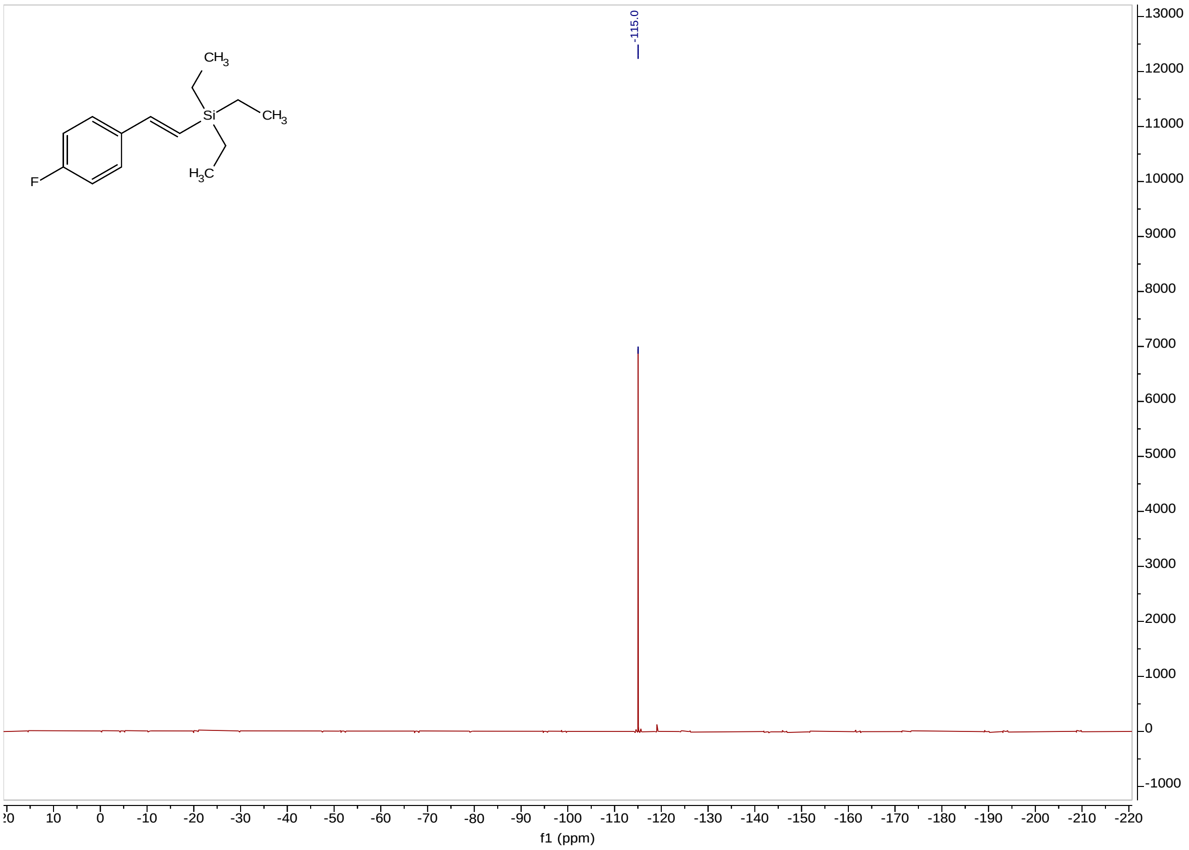
CH3
Si
CH3
H3C
F
-115.0
13000
12000
11000
10000
9000
8000
7000
6000
5000
4000
3000
2000
1000
0
-1000
10
0
-10
-20
-30
-40
-50
-60
-70
-80
-90
-100
-110
-120
-130
-140
-150
-160
-170
-180
-190
-200
-210
-220
f1 (ppm)

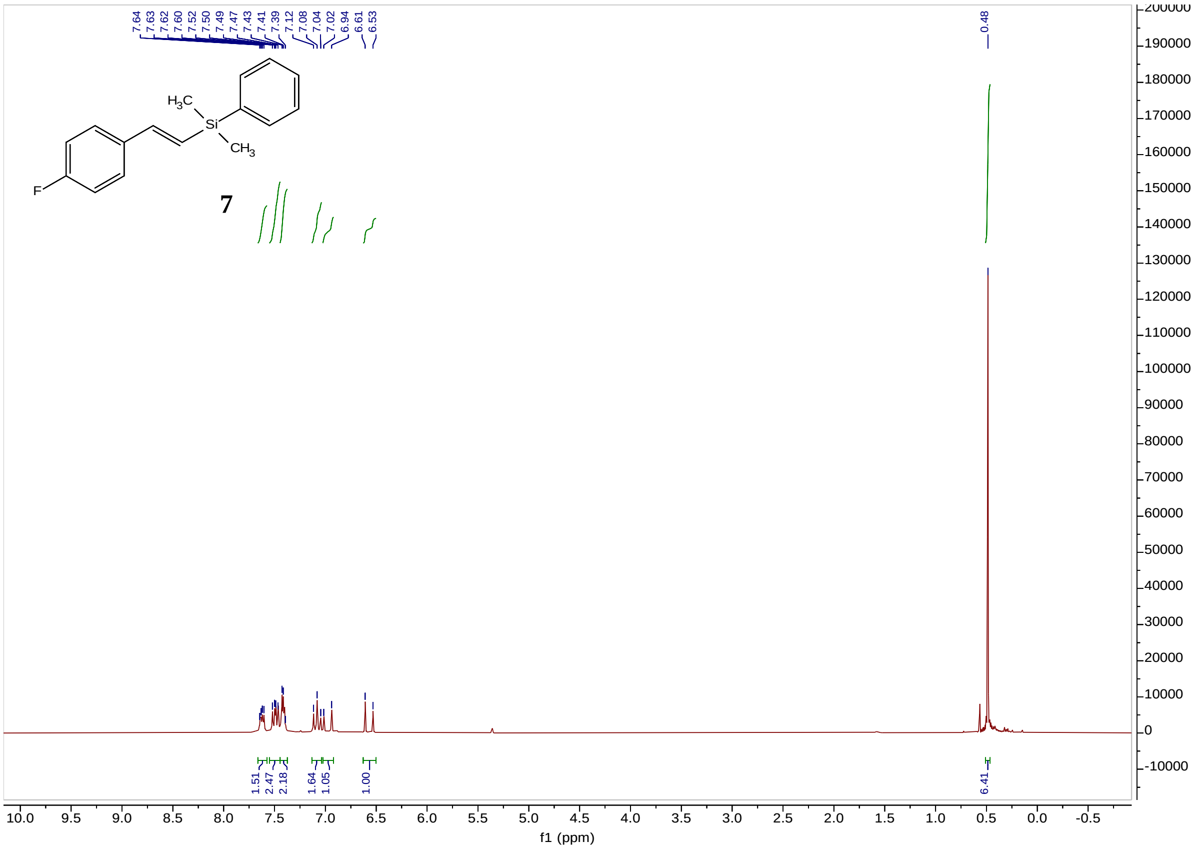
7.64
7.63
7.62
7.60
7.52
7.50
7.49
7.47
7.43
7.41
7.39
7.12
7.08
7.04
7.02
6.94
6.61
6.53
0.48
H3C
Si
CH3
F
7
1.51
2.47
2.18
1.64
1.05
1.00
6.41
f1 (ppm)

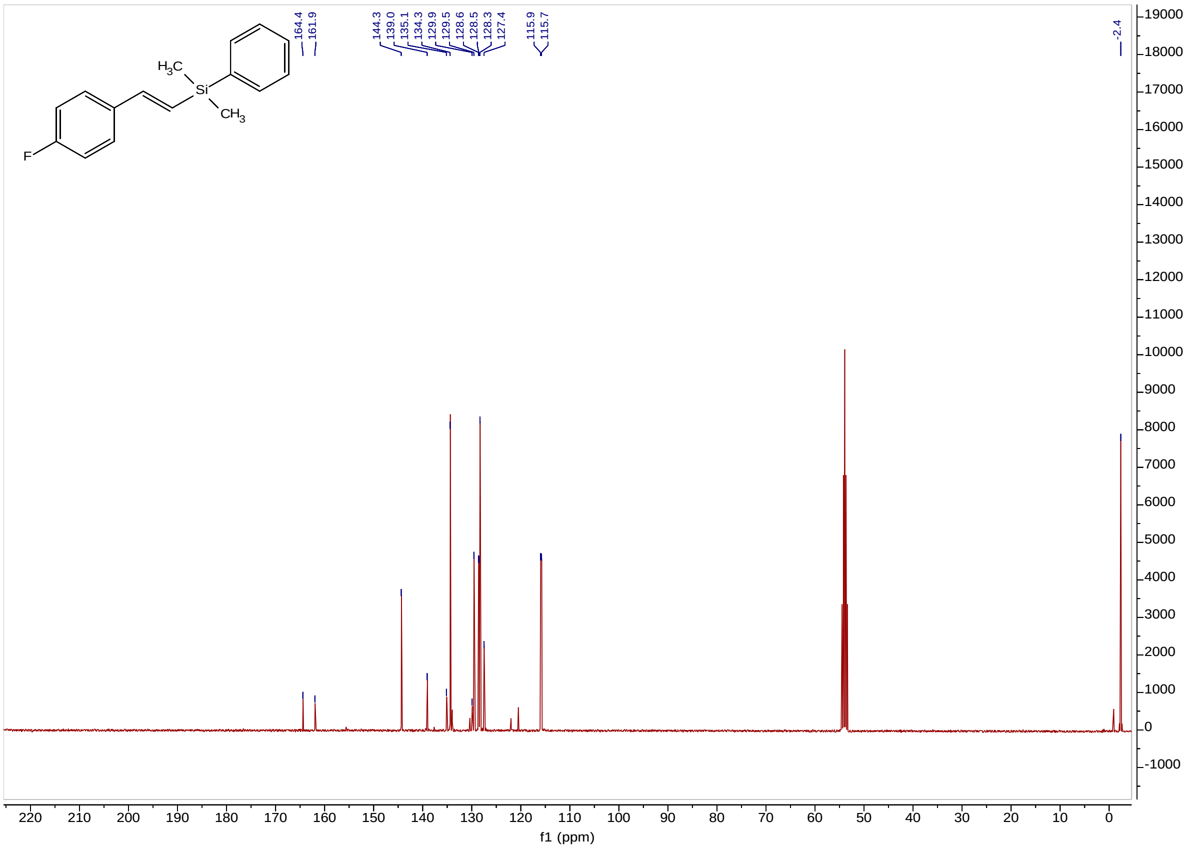
164.4
161.9
144.3
139.0
135.1
134.3
129.9
129.5
128.6
128.5
128.3
127.4
115.9
115.7
-2.4
H3C
Si
CH3
F
f1 (ppm)

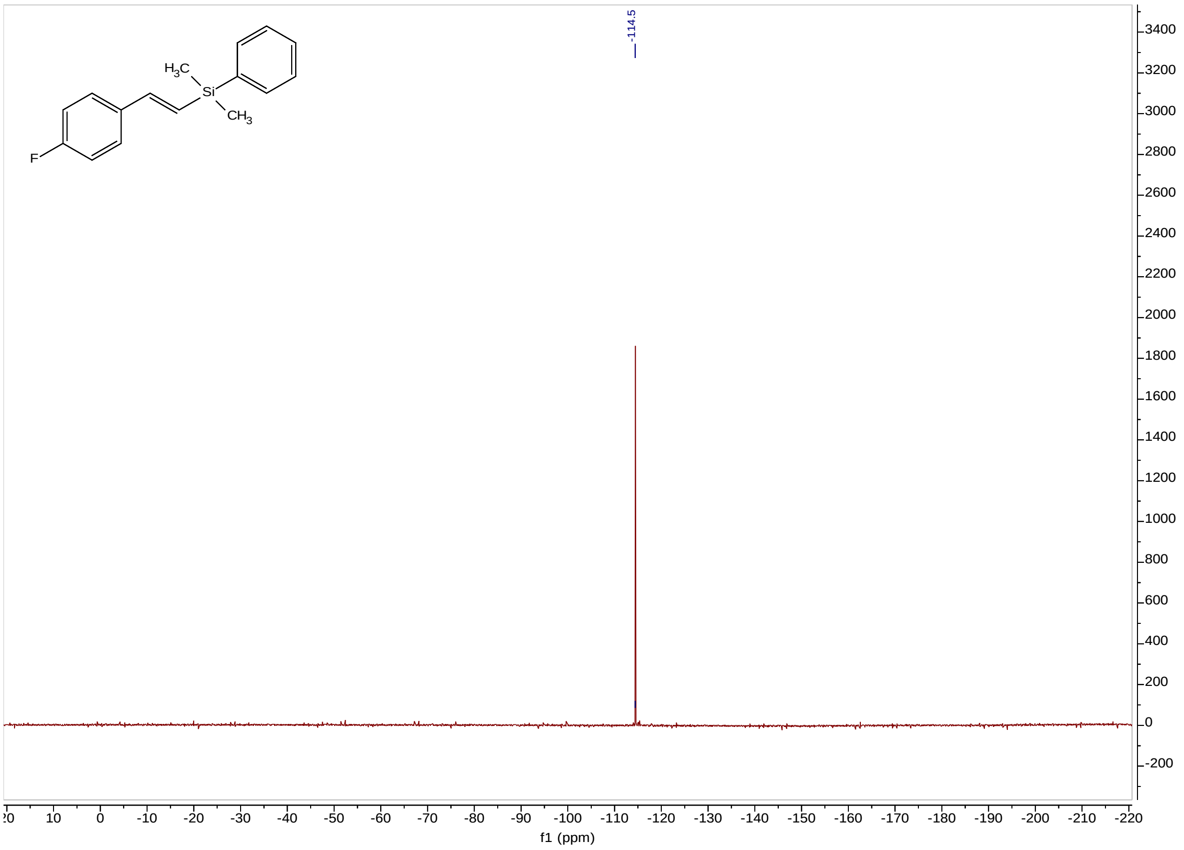

-114.5
F
H3C
Si
CH3
3400
3200
3000
2800
2600
2400
2200
2000
1800
1600
1400
1200
1000
800
600
400
200
0
-200
20
10
0
-10
-20
-30
-40
-50
-60
-70
-80
-90
-100
-110
-120
-130
-140
-150
-160
-170
-180
-190
-200
-210
-220
f1 (ppm)

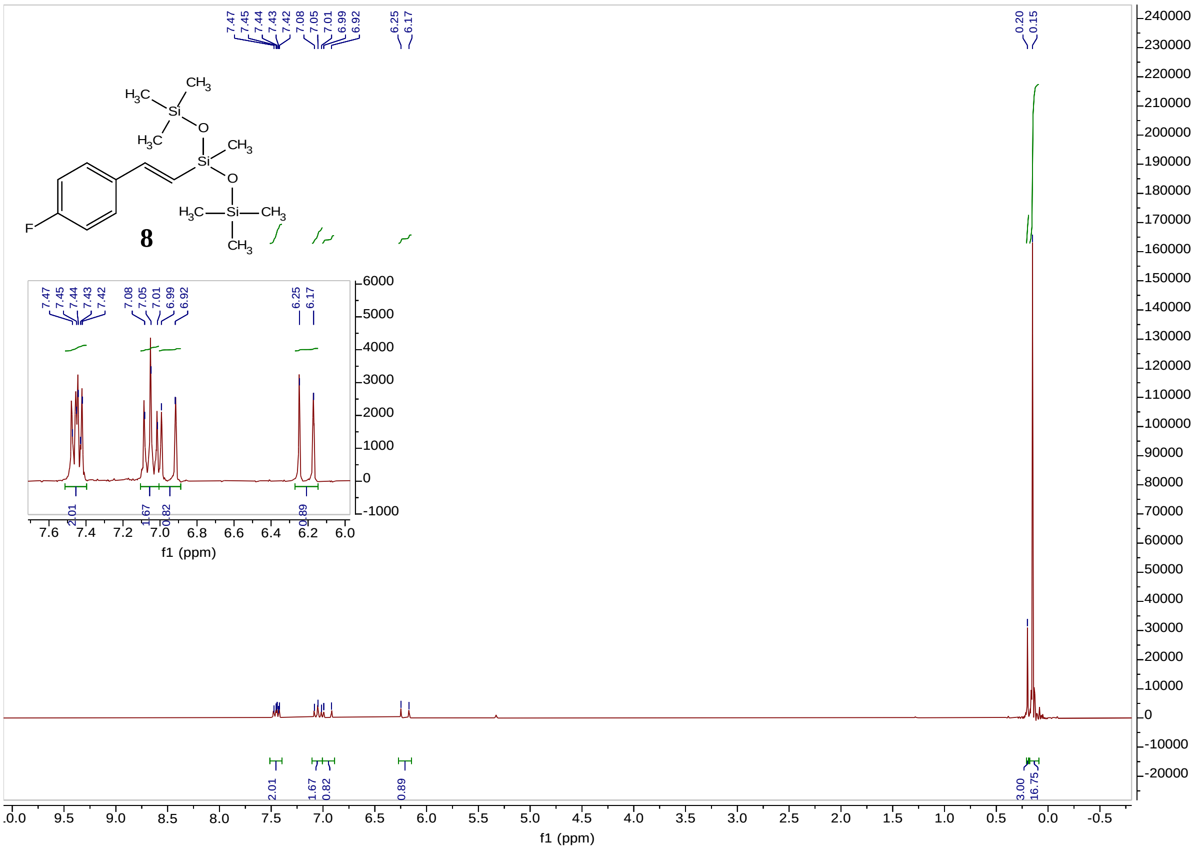

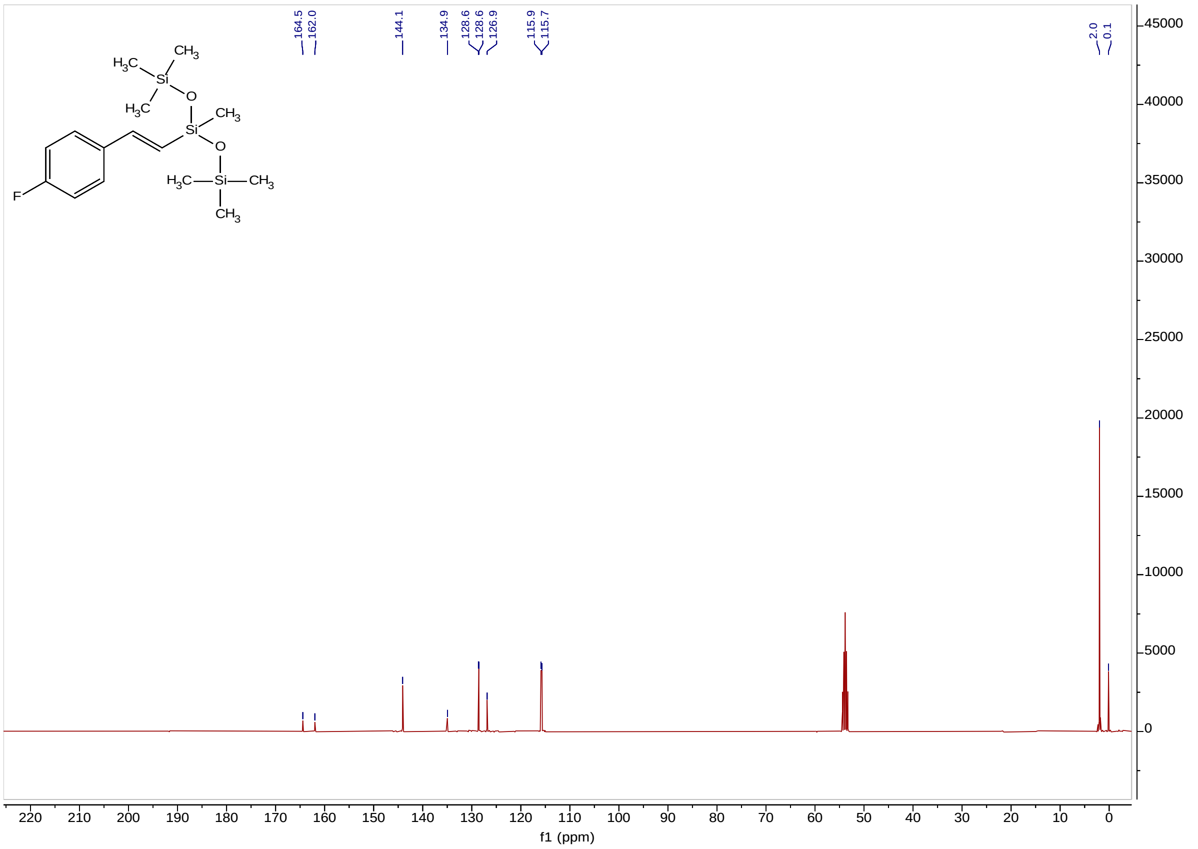

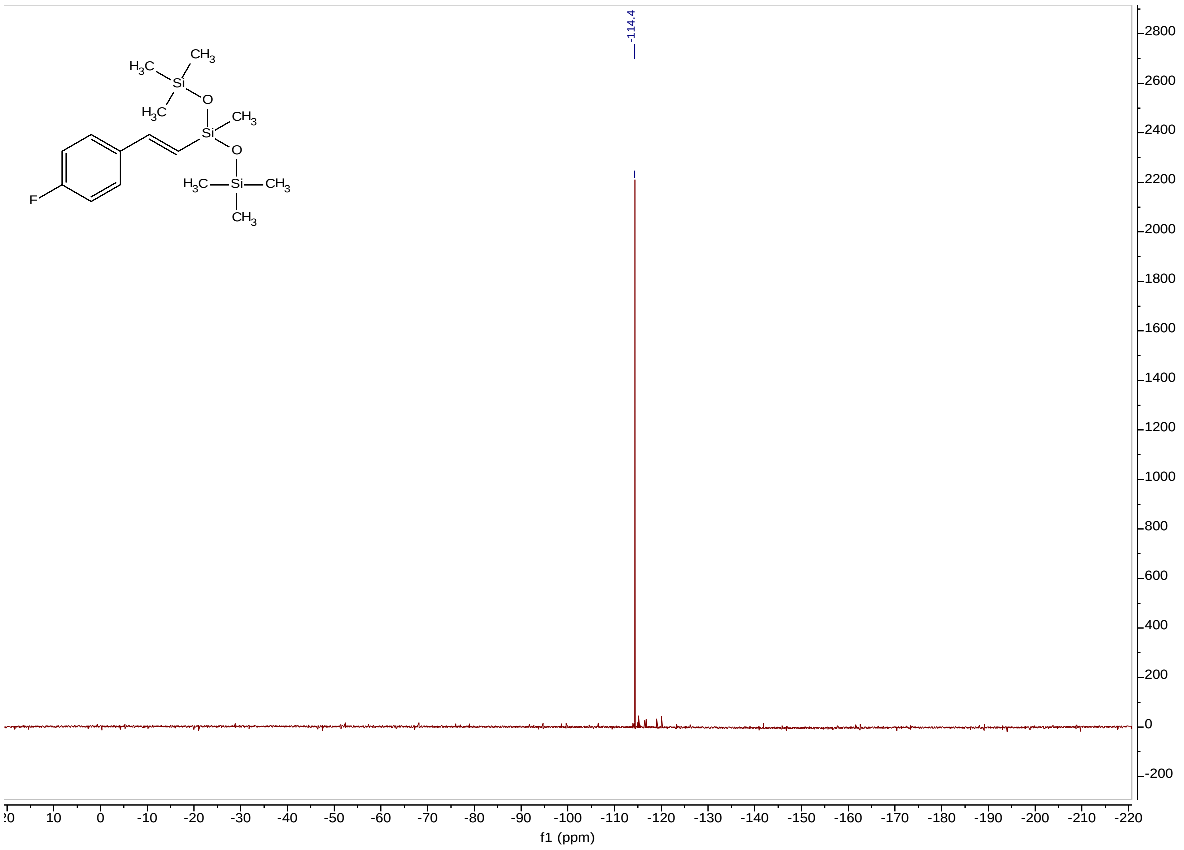

-114.4
H3C
CH3
Si
O
CH3
Si
O
H3C—Si—CH3
CH3
F
f1 (ppm)

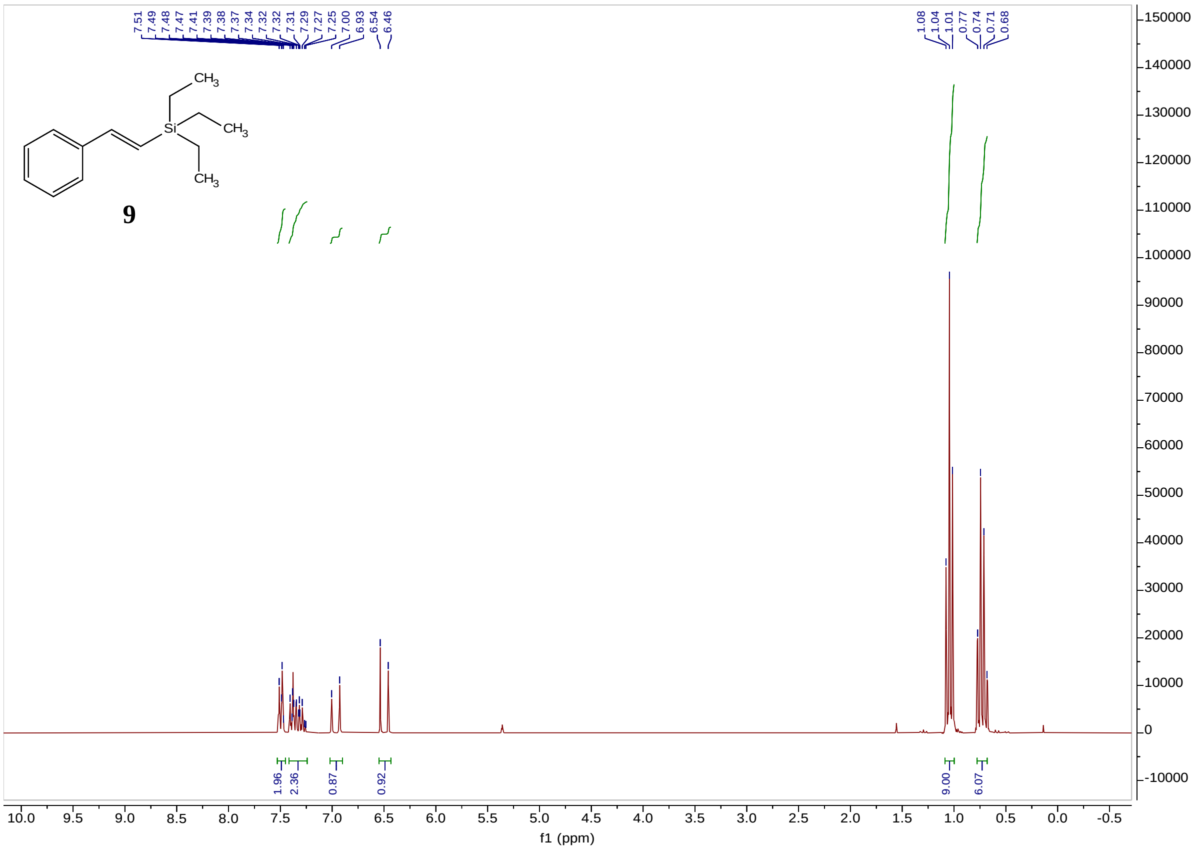
7.51
7.49
7.48
7.47
7.41
7.39
7.38
7.37
7.34
7.32
7.32
7.31
7.29
7.27
7.25
7.00
6.93
6.54
6.46
1.08
1.04
1.01
0.77
0.74
0.71
0.68
1.96
2.36
0.87
0.92
9.00
6.07
f1 (ppm)

9

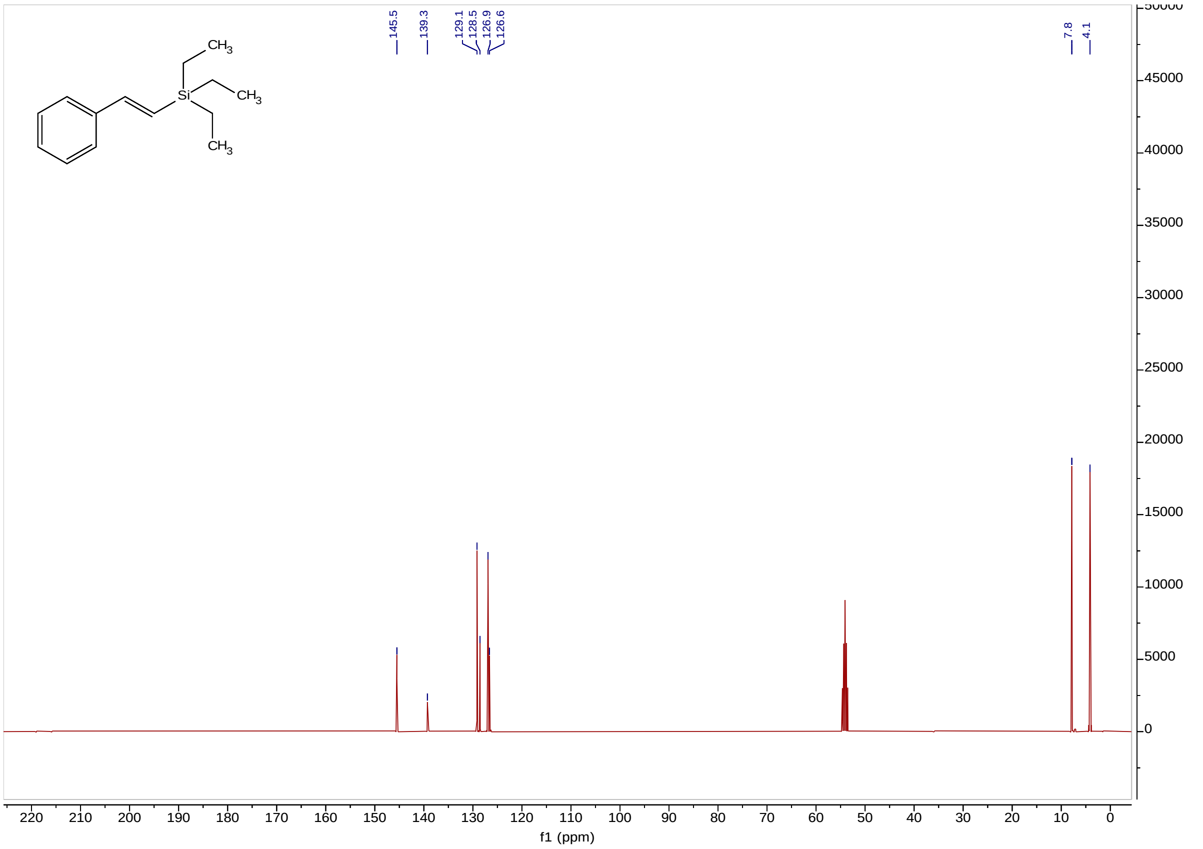
145.5
139.3
129.1
128.5
126.9
126.6
7.8
4.1
f1 (ppm)

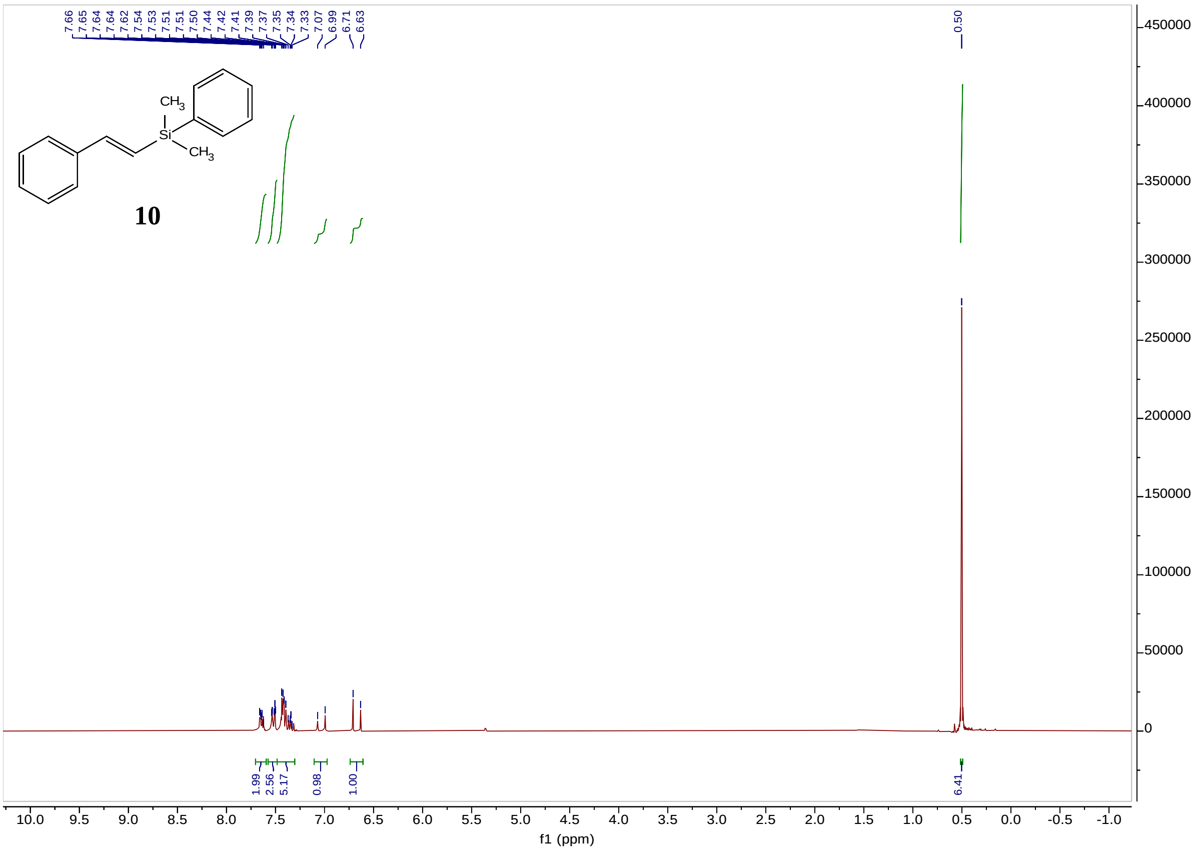
7.66
7.65
7.64
7.64
7.62
7.54
7.53
7.51
7.51
7.50
7.44
7.42
7.41
7.39
7.37
7.35
7.34
7.33
7.07
6.99
6.71
6.63
0.50
CH_3
Si
CH_3
10
1.99
2.56
5.17
0.98
1.00
6.41
f1 (ppm)

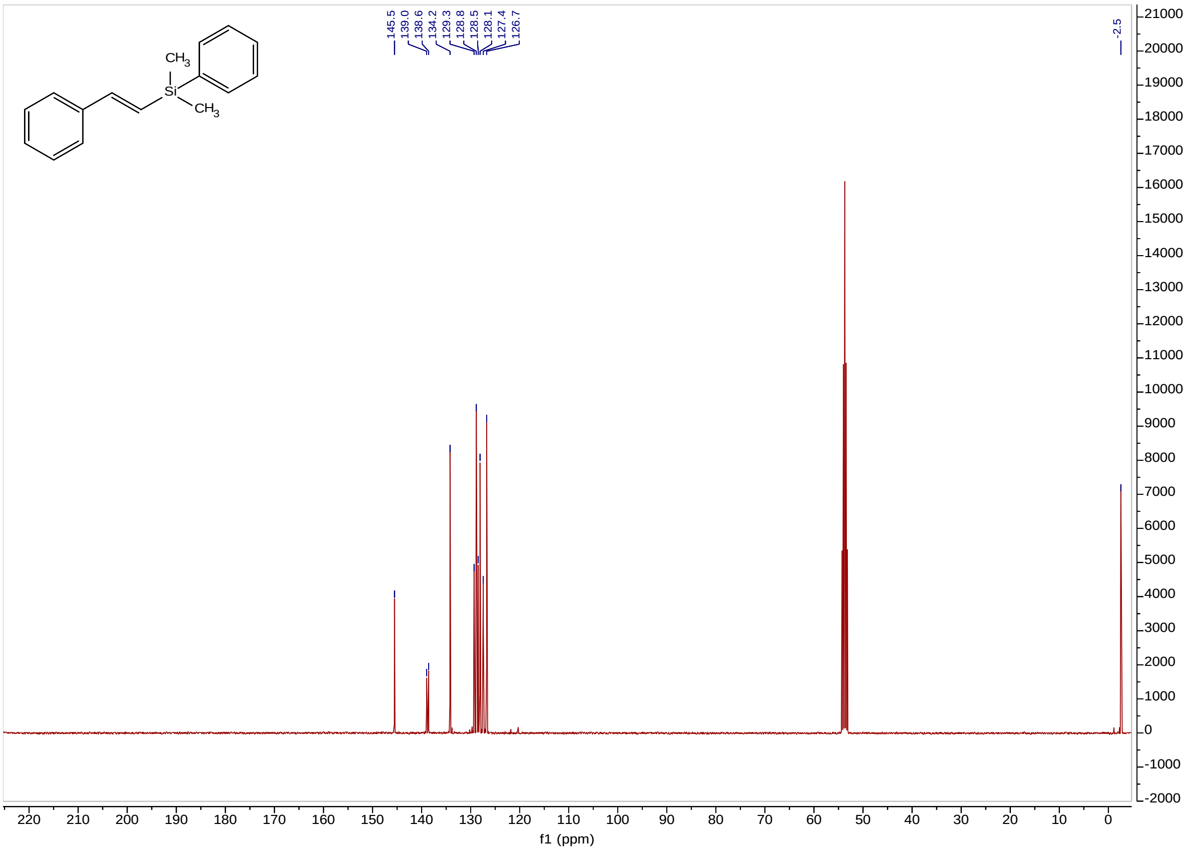
145.5
139.0
138.6
134.2
129.3
128.8
128.5
128.1
127.4
126.7
-2.5
CH_3
Si
CH_3
f1 (ppm)

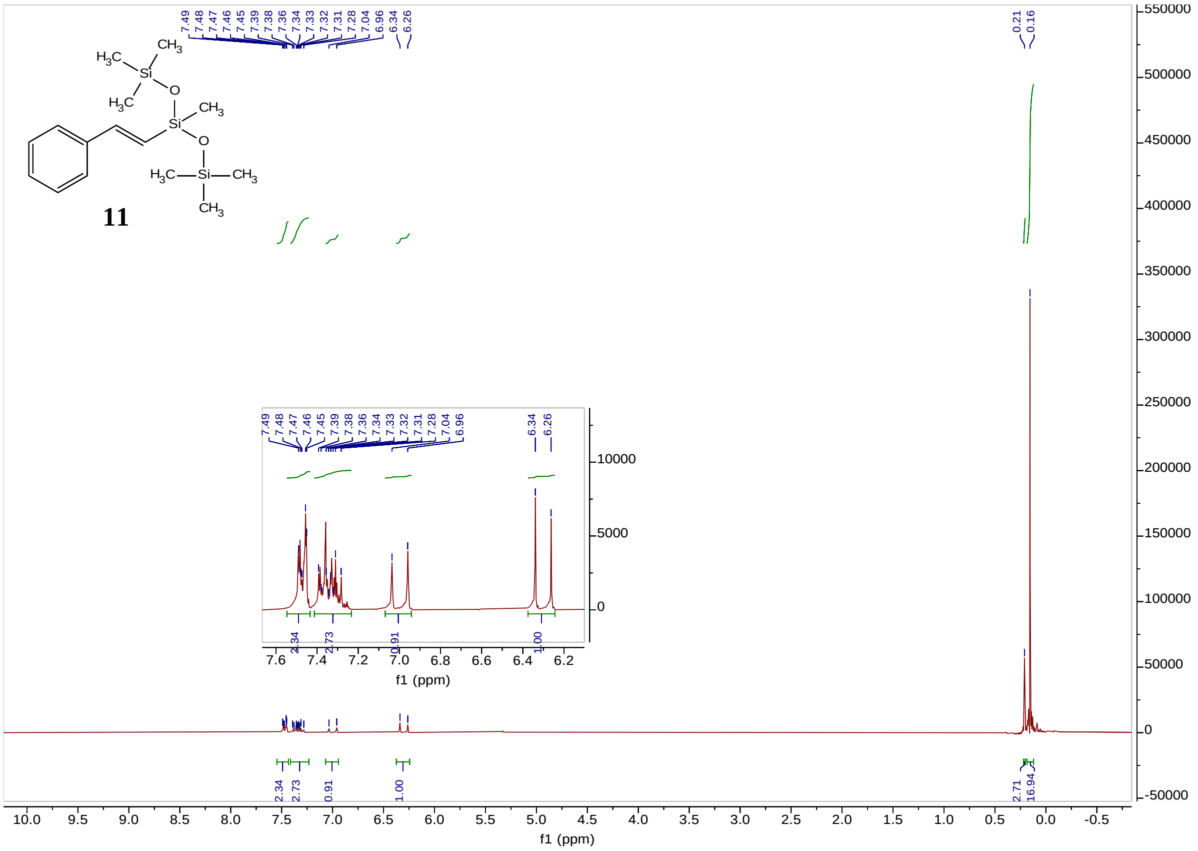
11
7.49 7.48 7.47 7.46 7.45 7.39 7.38 7.36 7.34 7.33 7.32 7.31 7.28 7.04 6.96 6.34 6.26
0.21 0.16
2.34 2.73 0.91 1.00 2.71 16.94
f1 (ppm)

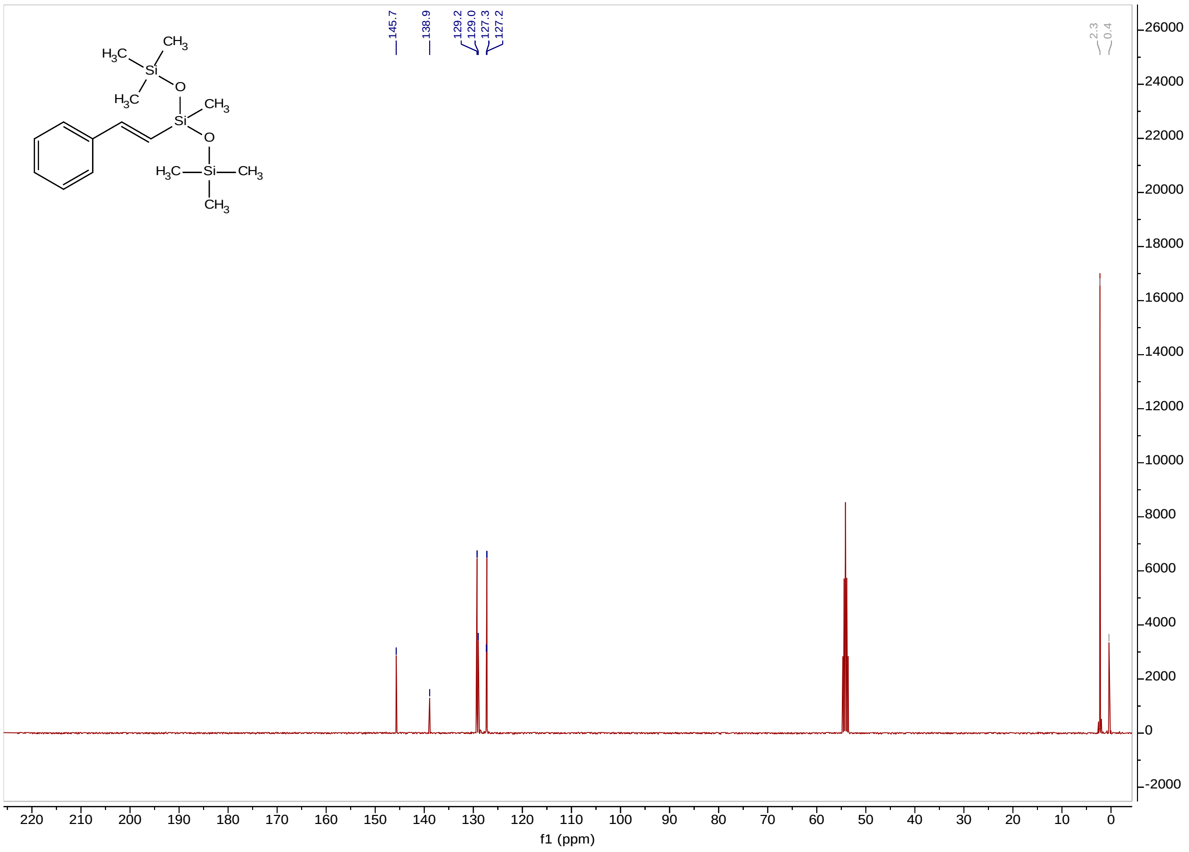
145.7 138.9 129.2 129.0 127.3 127.2
2.3 0.4
f1 (ppm)

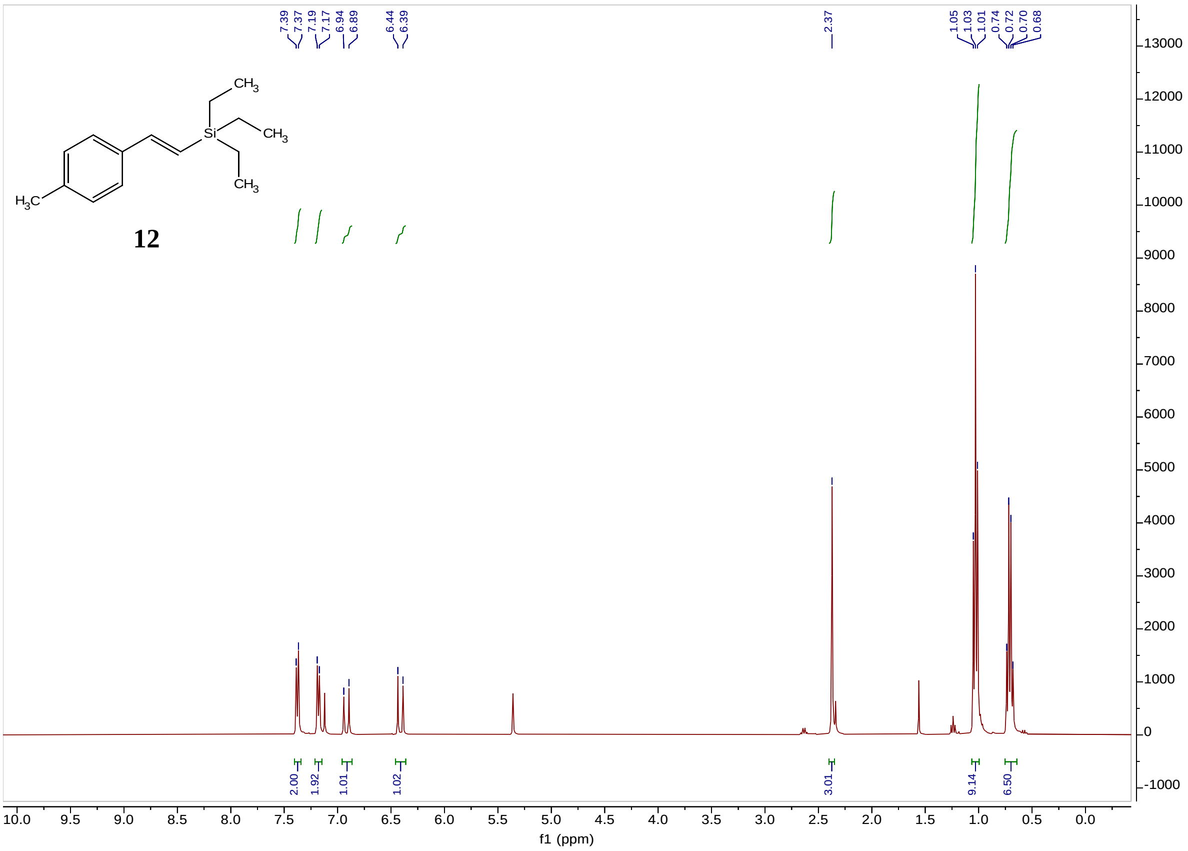
7.39
7.37
7.19
7.17
6.94
6.89
6.44
6.39
2.37
1.05
1.03
1.01
0.74
0.72
0.70
0.68
2.00
1.92
1.01
1.02
3.01
9.14
6.50
f1 (ppm)

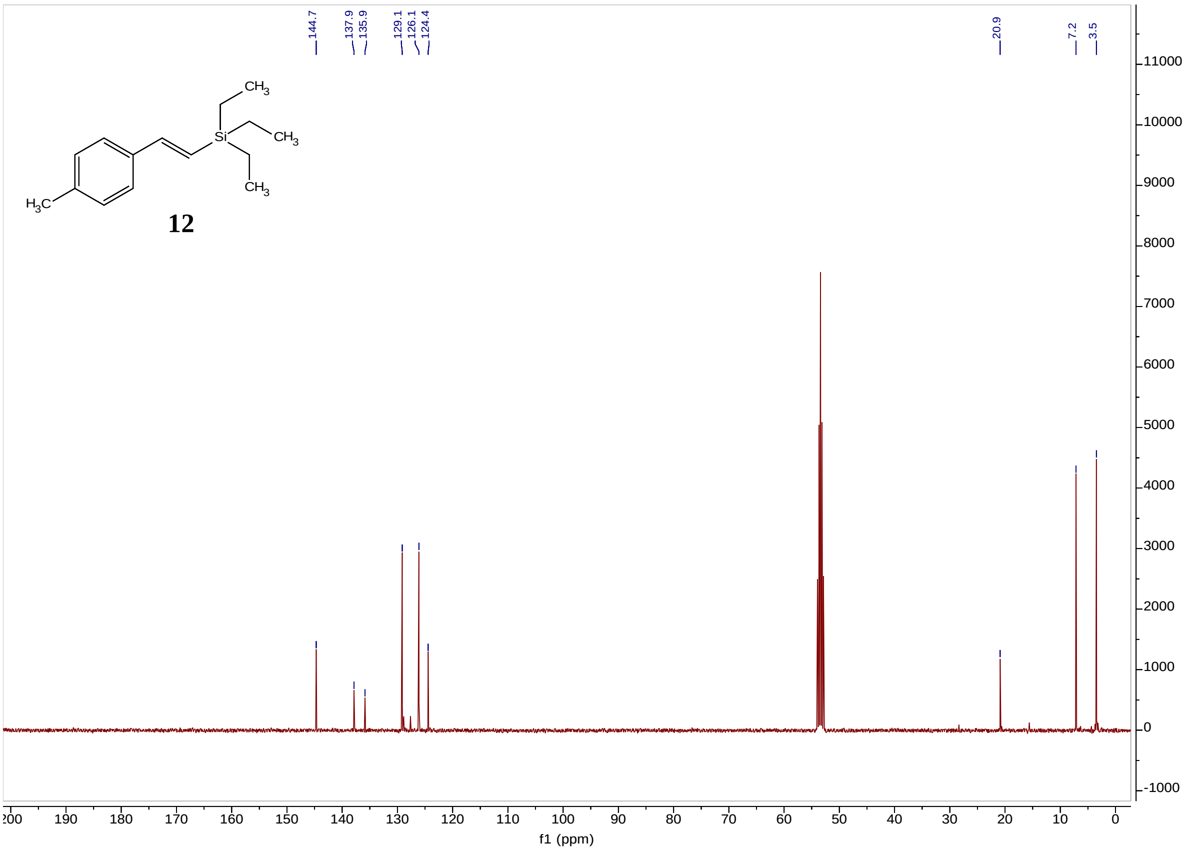
144.7
137.9
135.9
129.1
126.1
124.4
20.9
7.2
3.5
f1 (ppm)

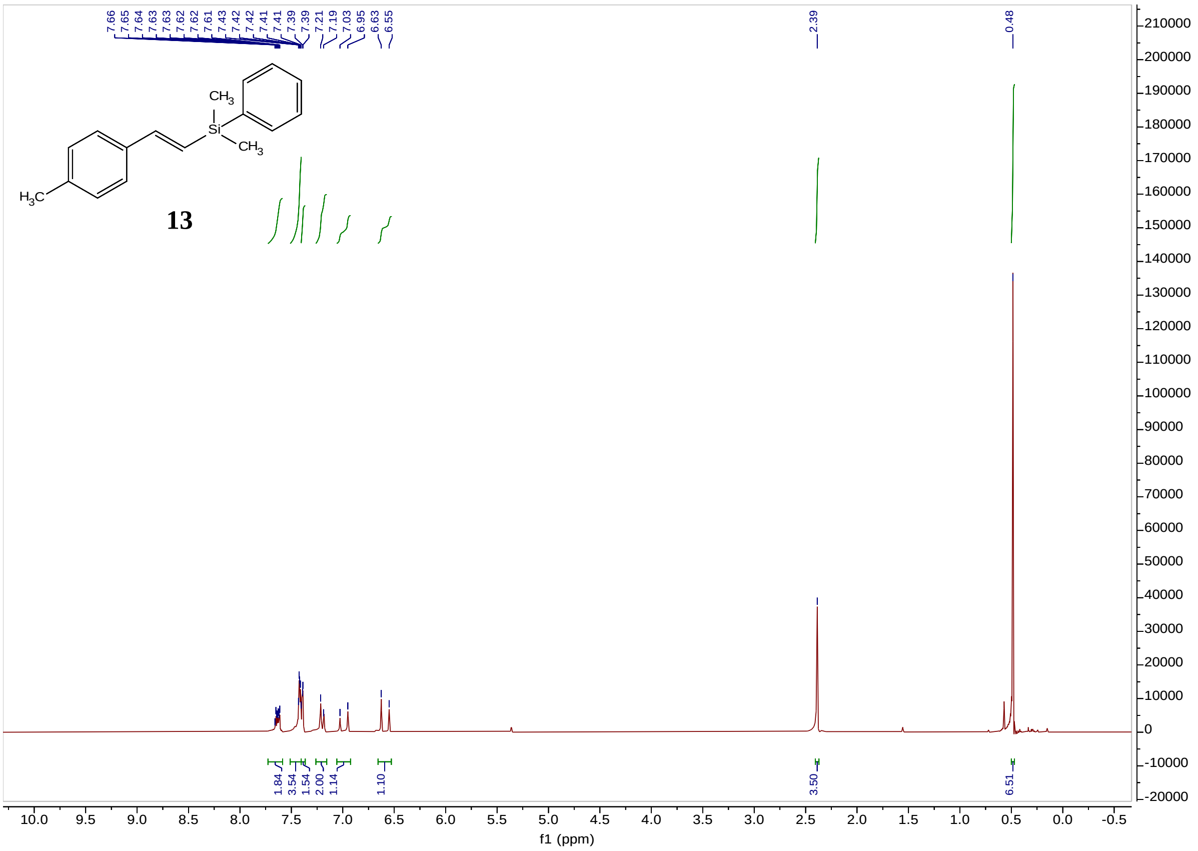
7.66
7.65
7.64
7.63
7.63
7.62
7.62
7.61
7.43
7.42
7.42
7.41
7.39
7.39
7.21
7.19
7.03
6.95
6.63
6.55
2.39
0.48
CH3
Si
CH3
H3C
13
1.84
3.54
1.54
2.00
1.14
1.10
3.50
6.51
f1 (ppm)

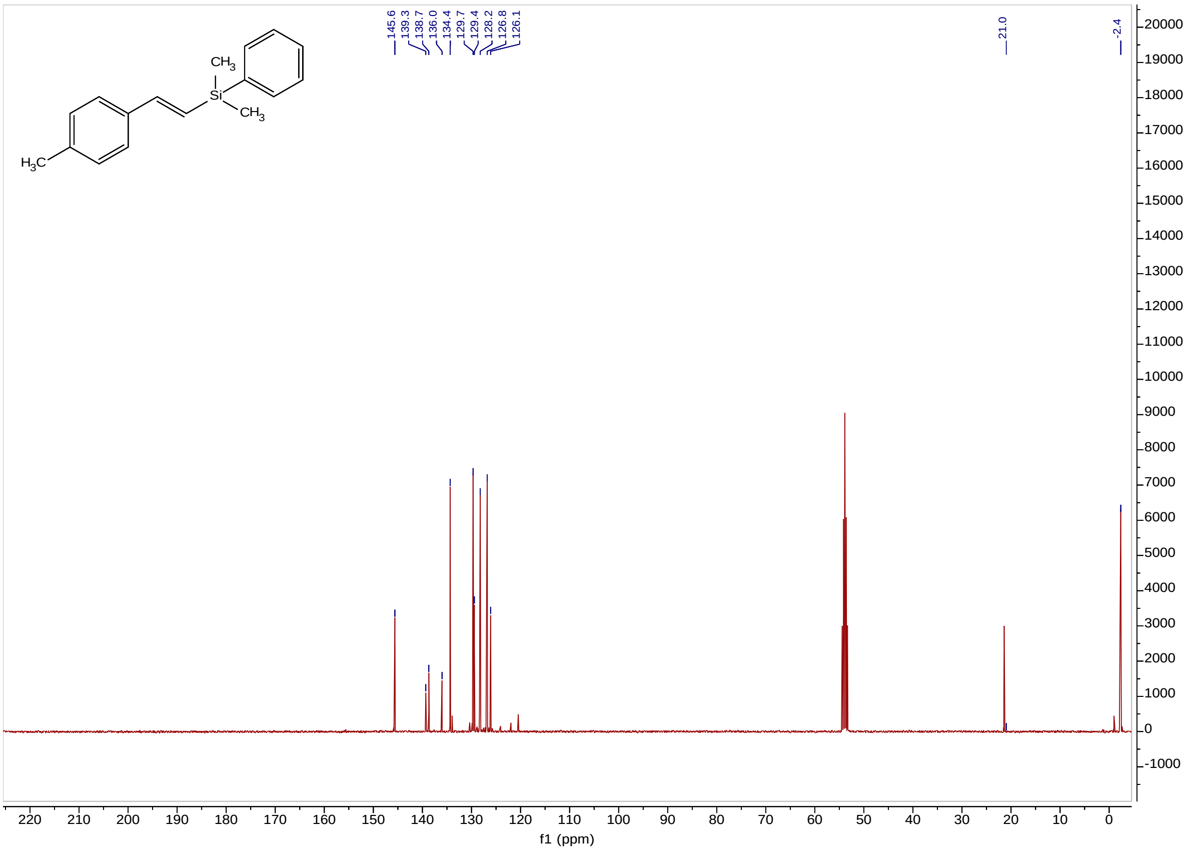
145.6
139.3
138.7
136.0
134.4
129.7
129.4
128.2
126.8
126.1
21.0
-2.4
CH3
Si
CH3
H3C
f1 (ppm)

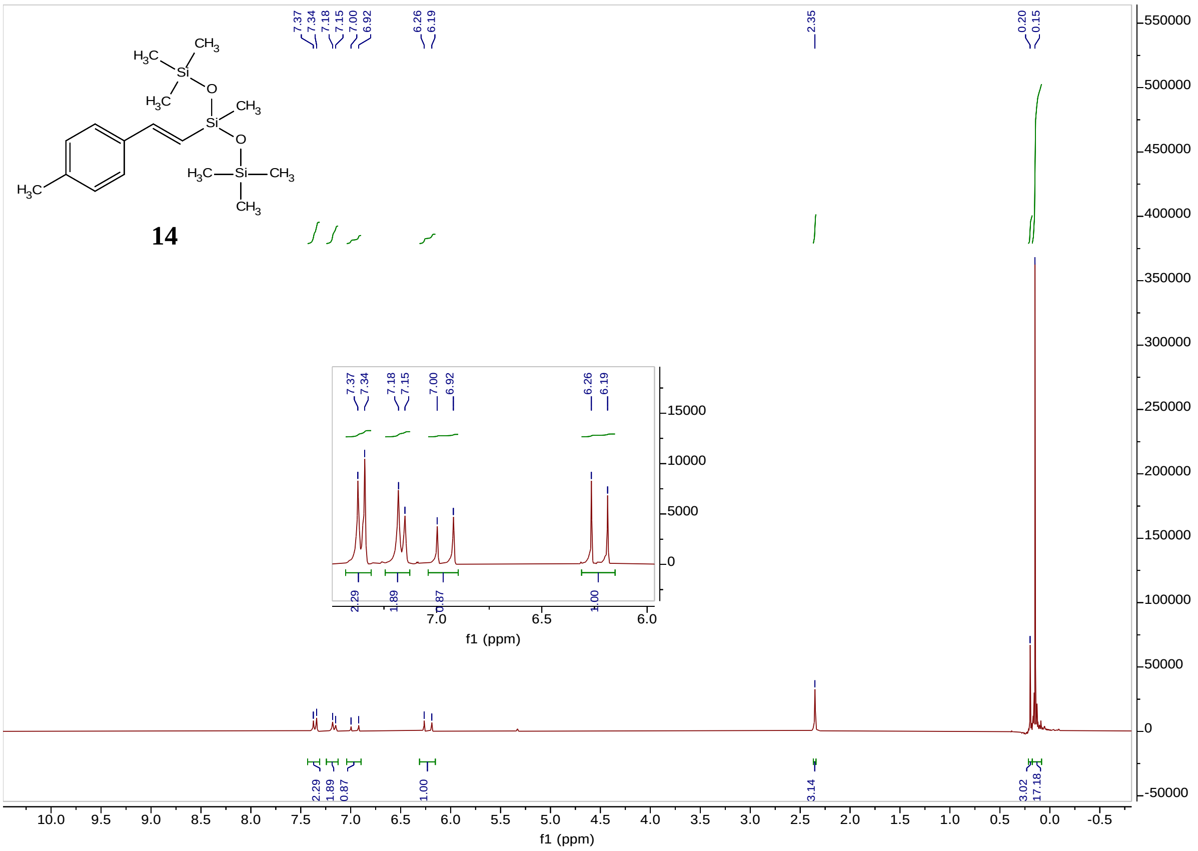
14
7.37
7.34
7.18
7.15
7.00
6.92
6.26
6.19
2.35
0.20
0.15
2.29
1.89
0.87
1.00
3.14
3.02
17.18
f1 (ppm)

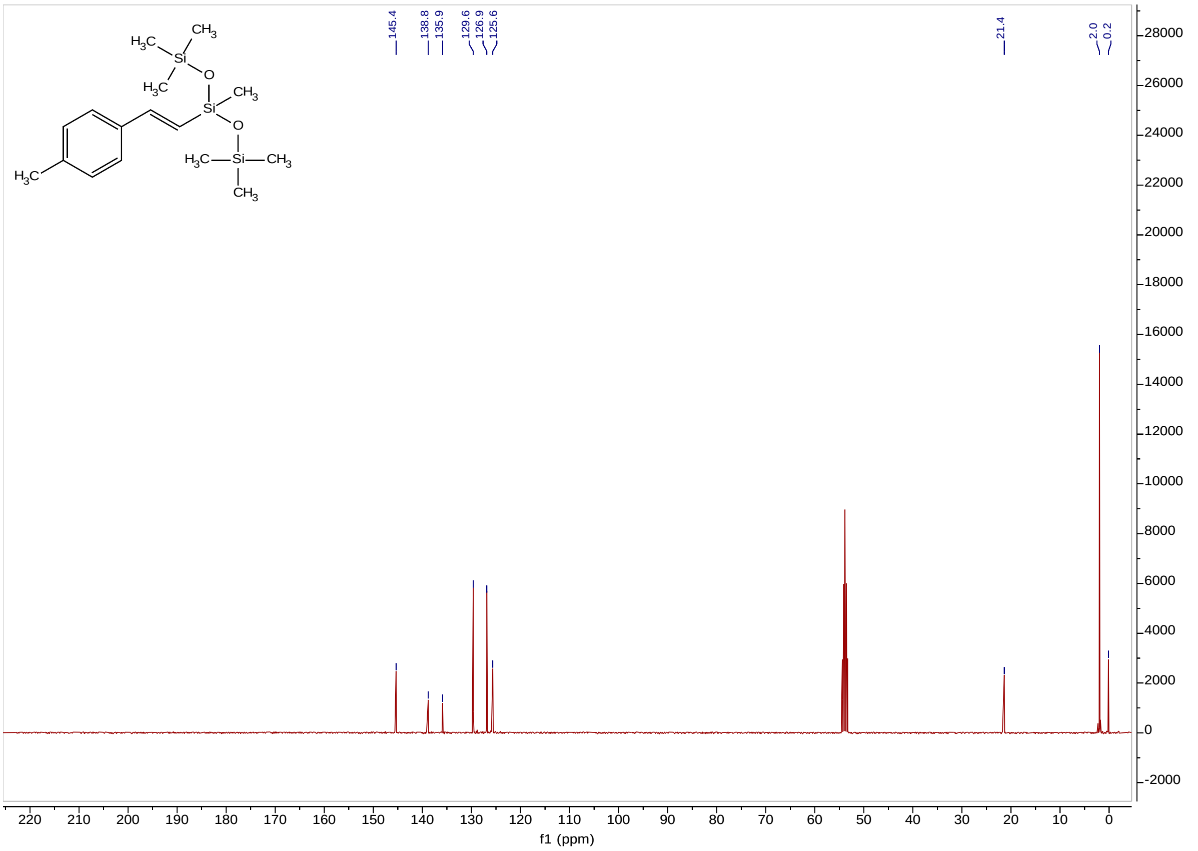
145.4
138.8
135.9
129.6
126.9
125.6
21.4
2.0
0.2
f1 (ppm)

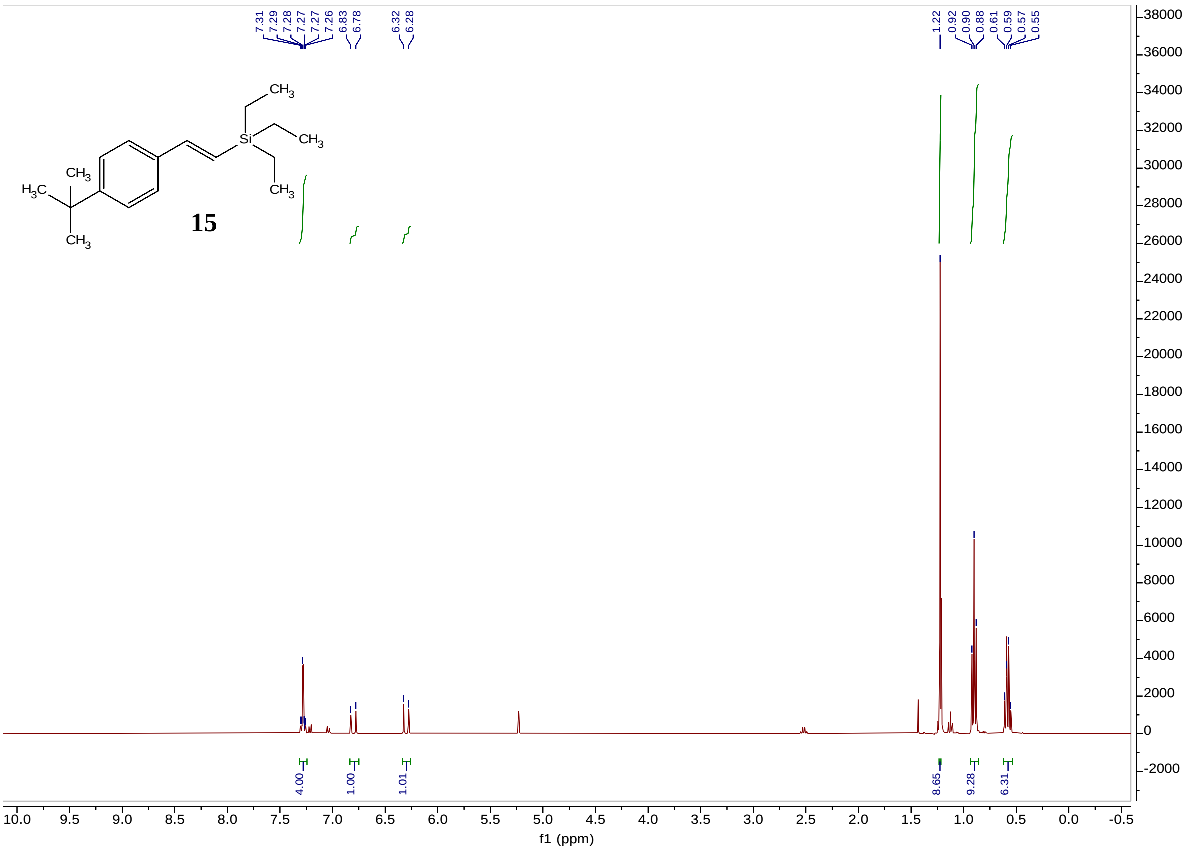

7.31
7.29
7.28
7.27
7.27
7.26
6.83
6.78
6.32
6.28
1.22
0.92
0.90
0.88
0.61
0.59
0.57
0.55
CH3
CH3
CH3
H3C
CH3
CH3
Si
15
4.00
1.00
1.01
8.65
9.28
6.31
f1 (ppm)

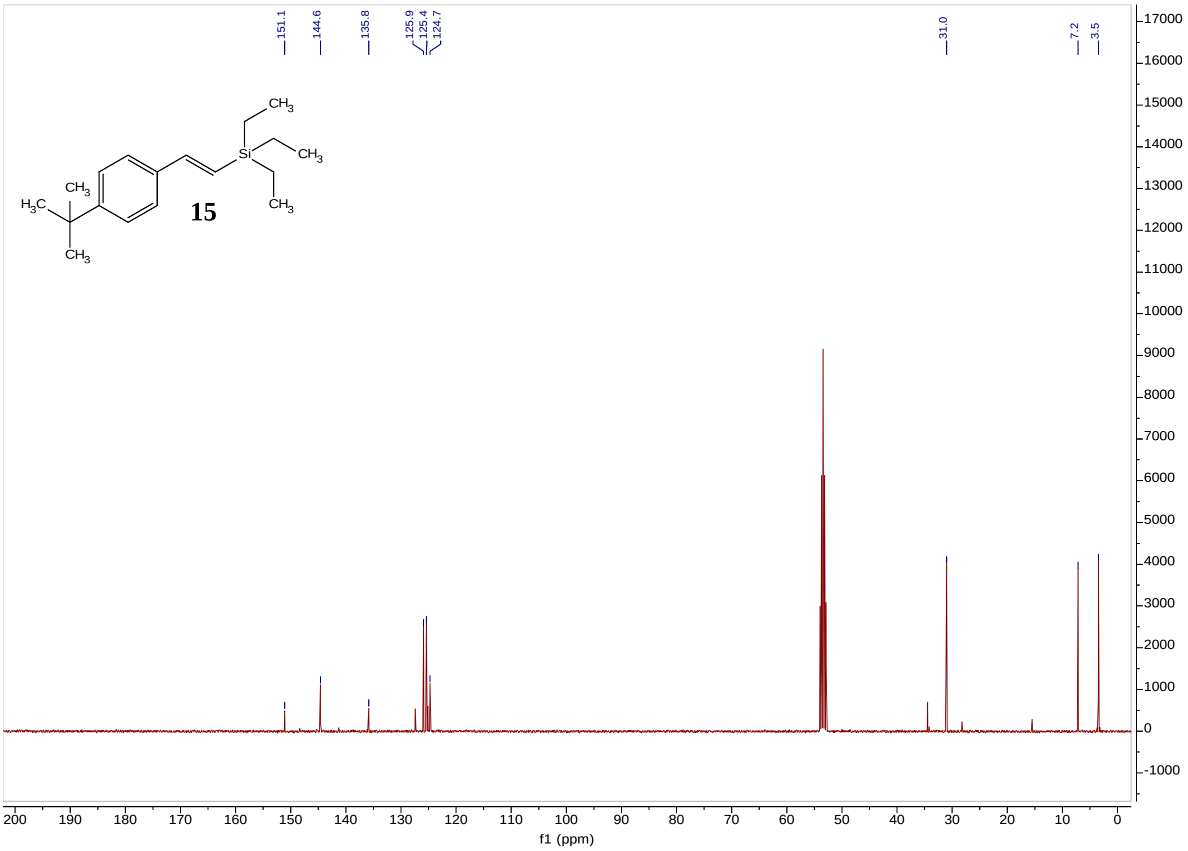

151.1
144.6
135.8
125.9
125.4
124.7
31.0
7.2
3.5
CH3
CH3
CH3
H3C
CH3
CH3
Si
15
f1 (ppm)

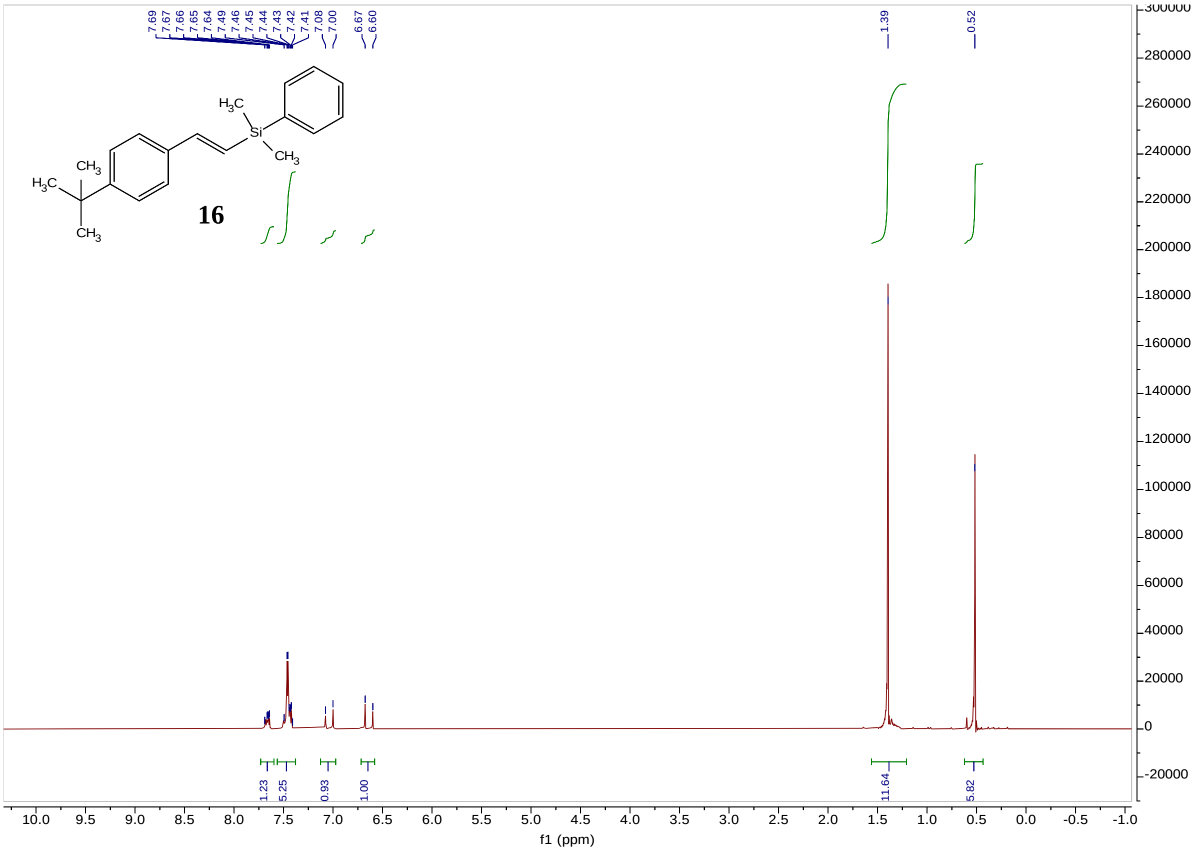
16
f1 (ppm)

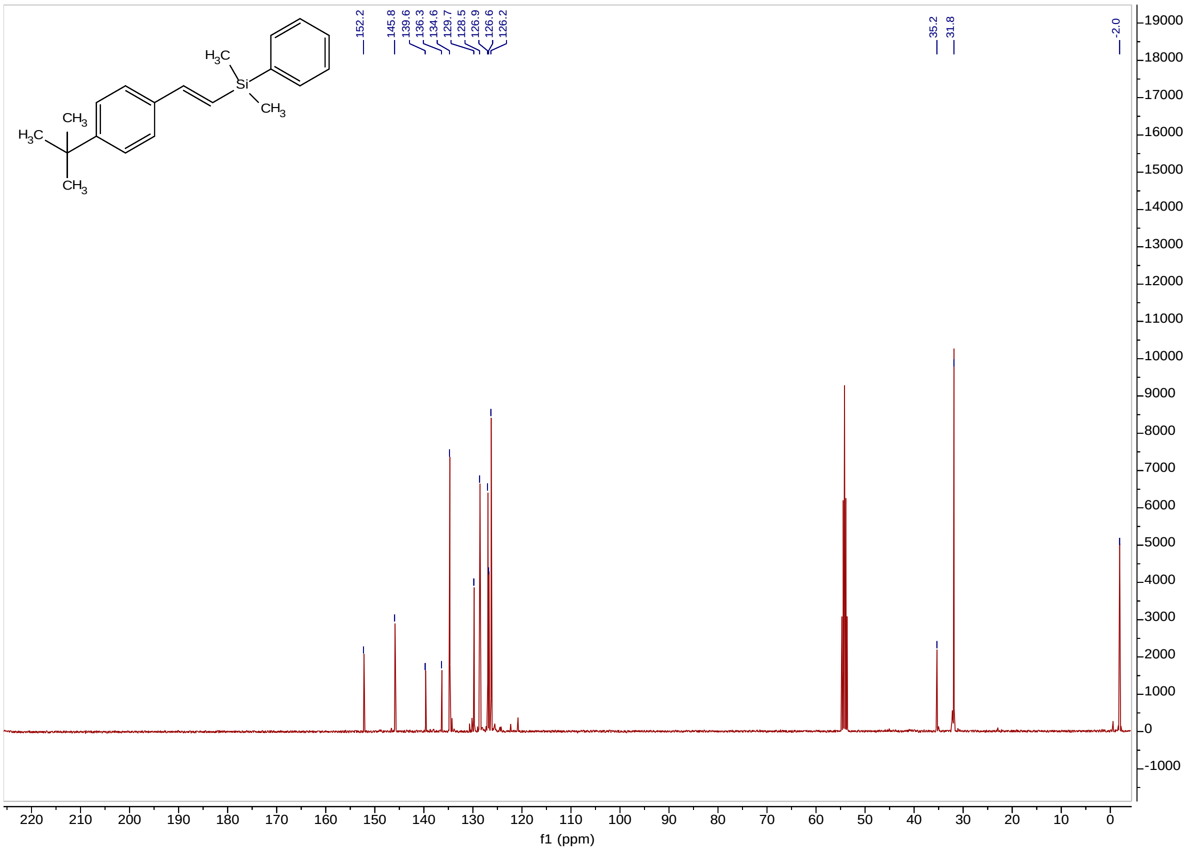
f1 (ppm)

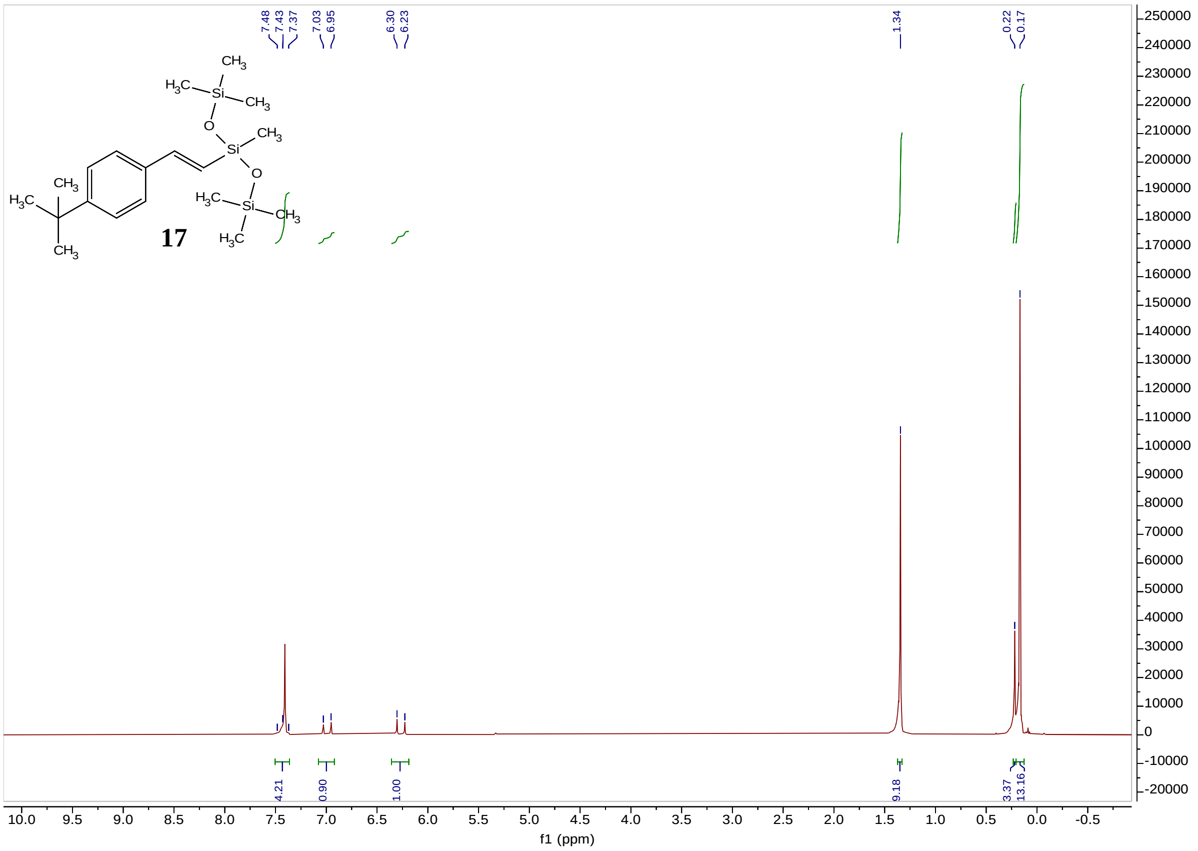

7.48
7.43
7.37
7.03
6.95
6.30
6.23
1.34
0.22
0.17
17
4.21
0.90
1.00
9.18
3.37
13.16
f1 (ppm)

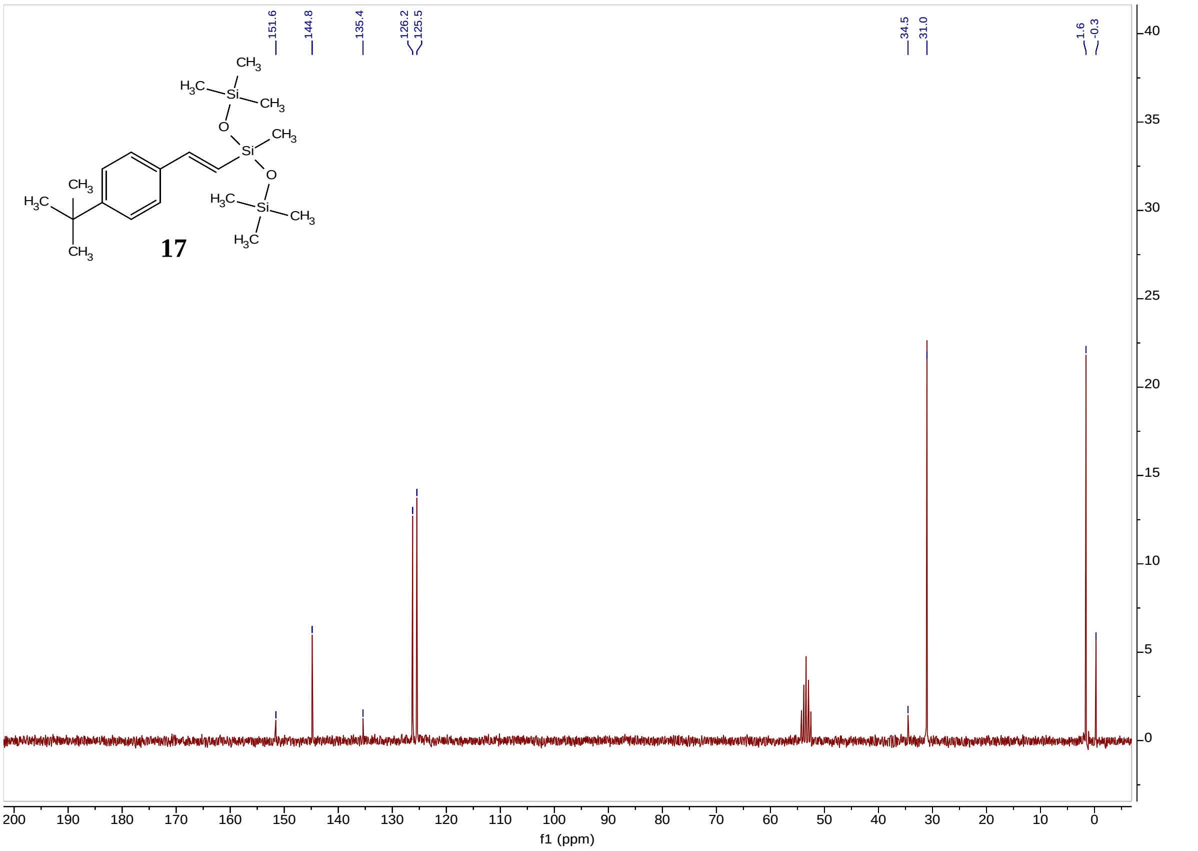

151.6
144.8
135.4
126.2
125.5
34.5
31.0
1.6
-0.3
17
f1 (ppm)

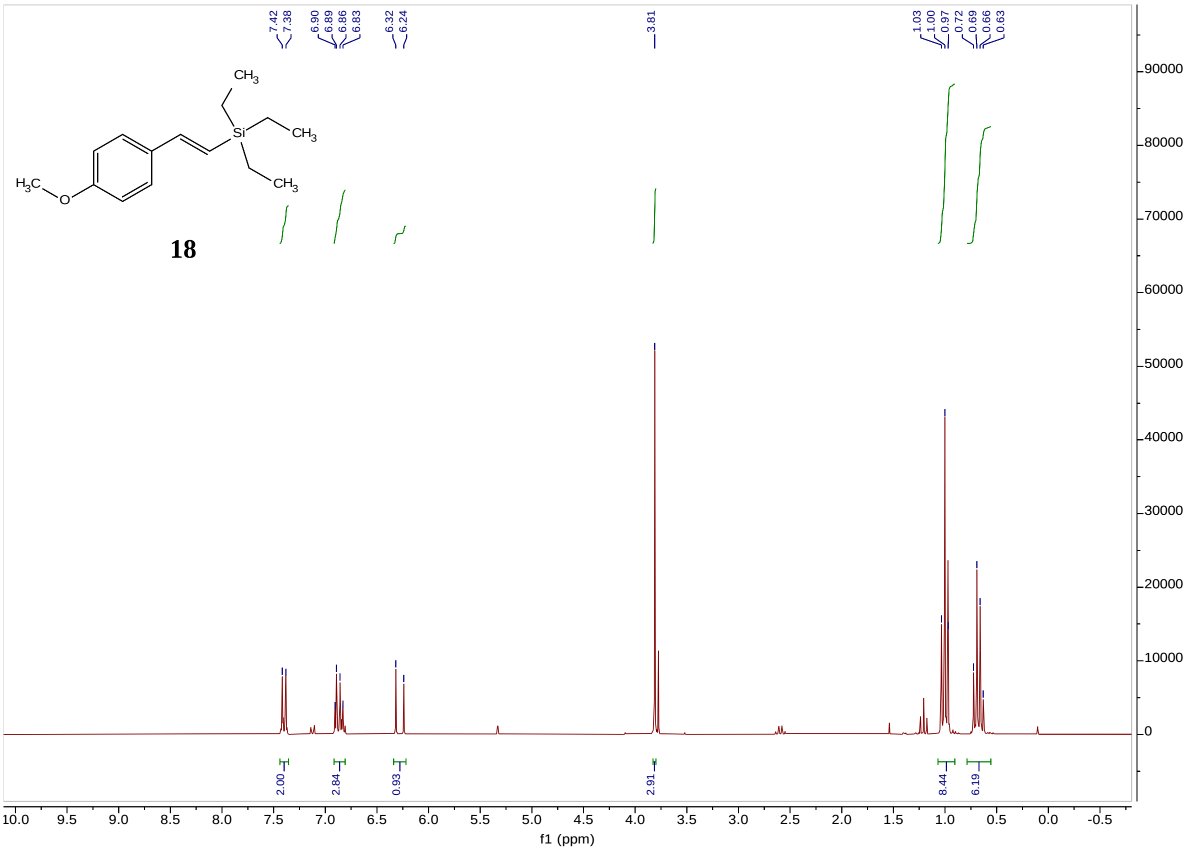
18
f1 (ppm)

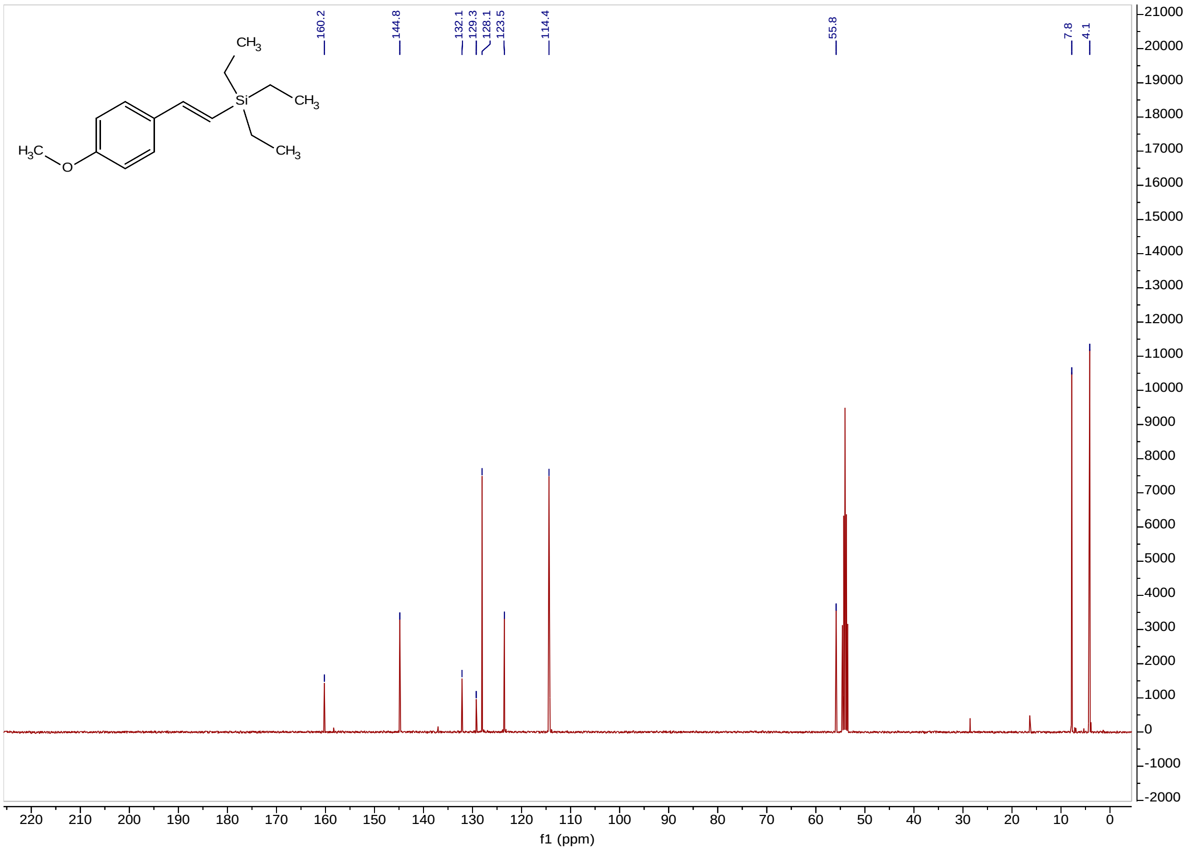
f1 (ppm)

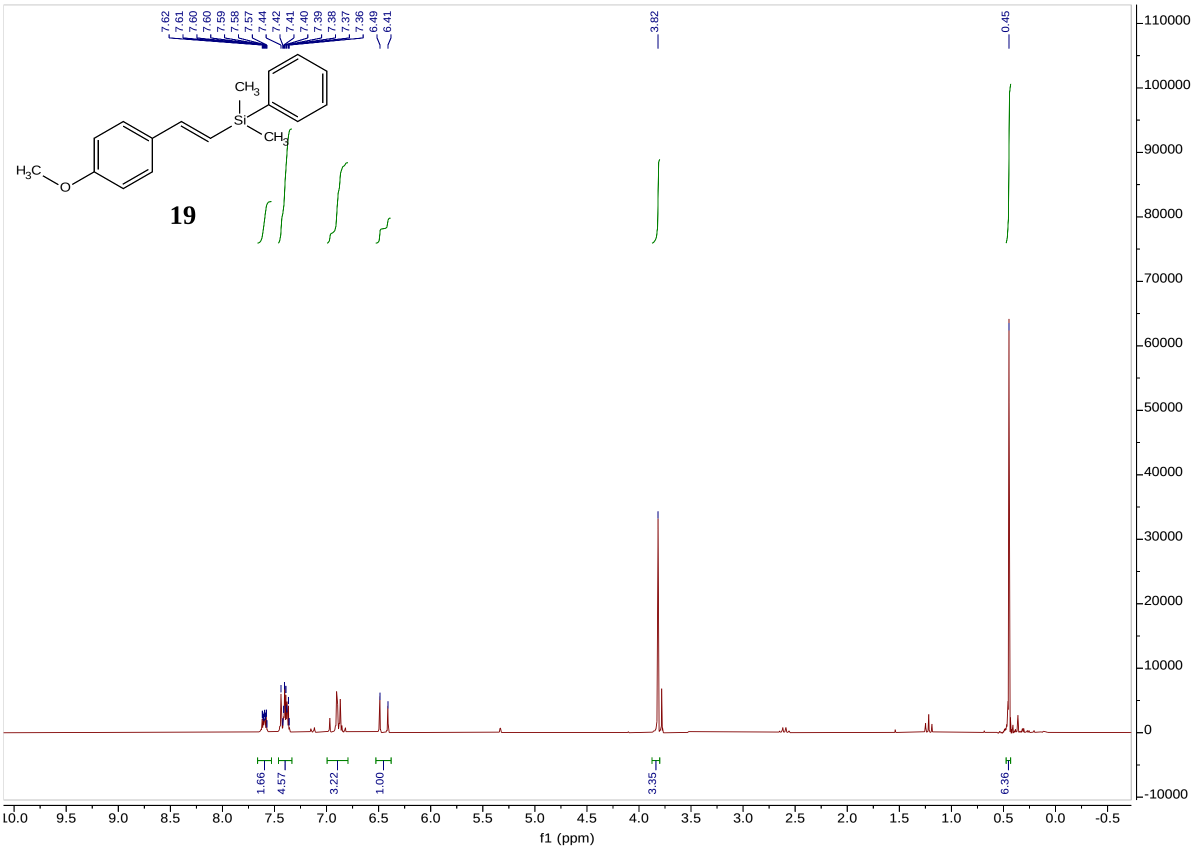
7.62
7.61
7.60
7.60
7.59
7.58
7.57
7.44
7.42
7.41
7.40
7.39
7.38
7.37
7.36
6.49
6.41
3.82
0.45
CH3
Si
CH3
H3C
O
19
1.66
4.57
3.22
1.00
3.35
6.36
f1 (ppm)

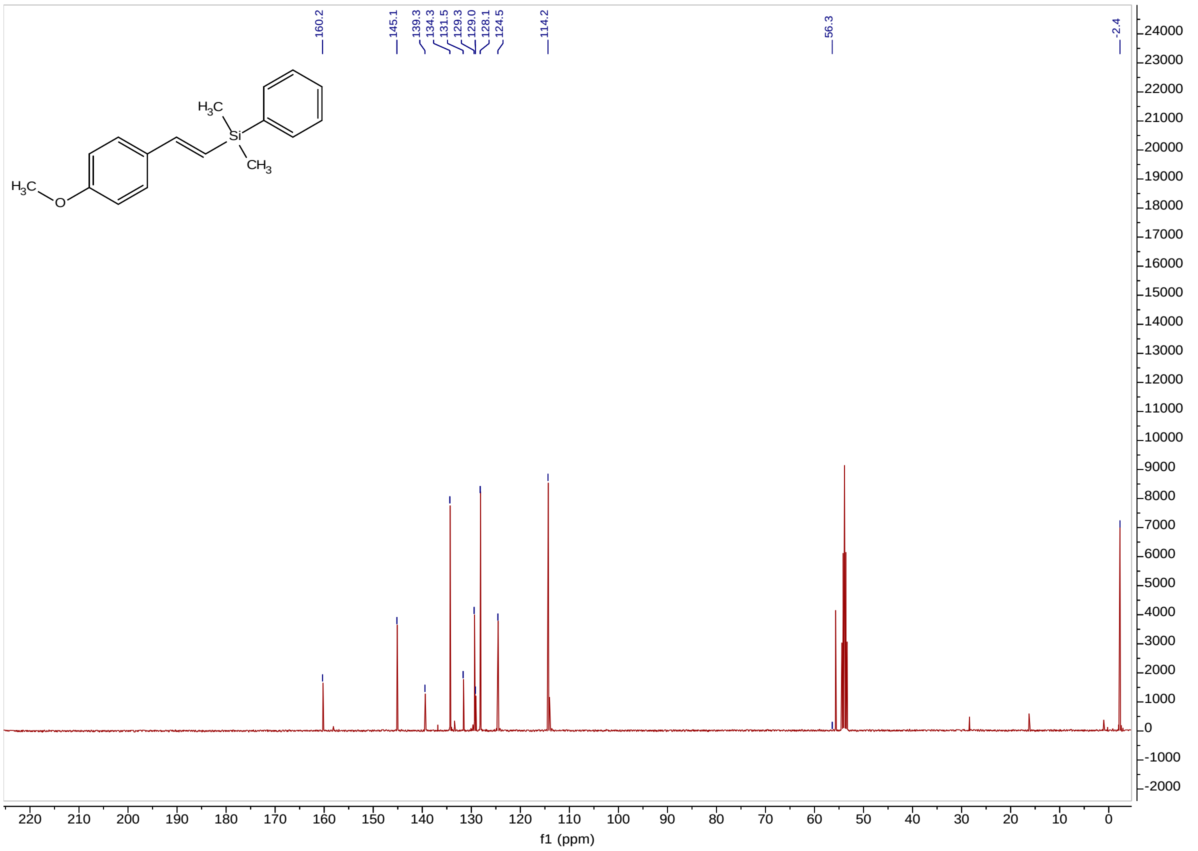
160.2
145.1
139.3
134.3
131.5
129.3
129.0
128.1
124.5
114.2
56.3
-2.4
H3C
Si
CH3
H3C
O
f1 (ppm)

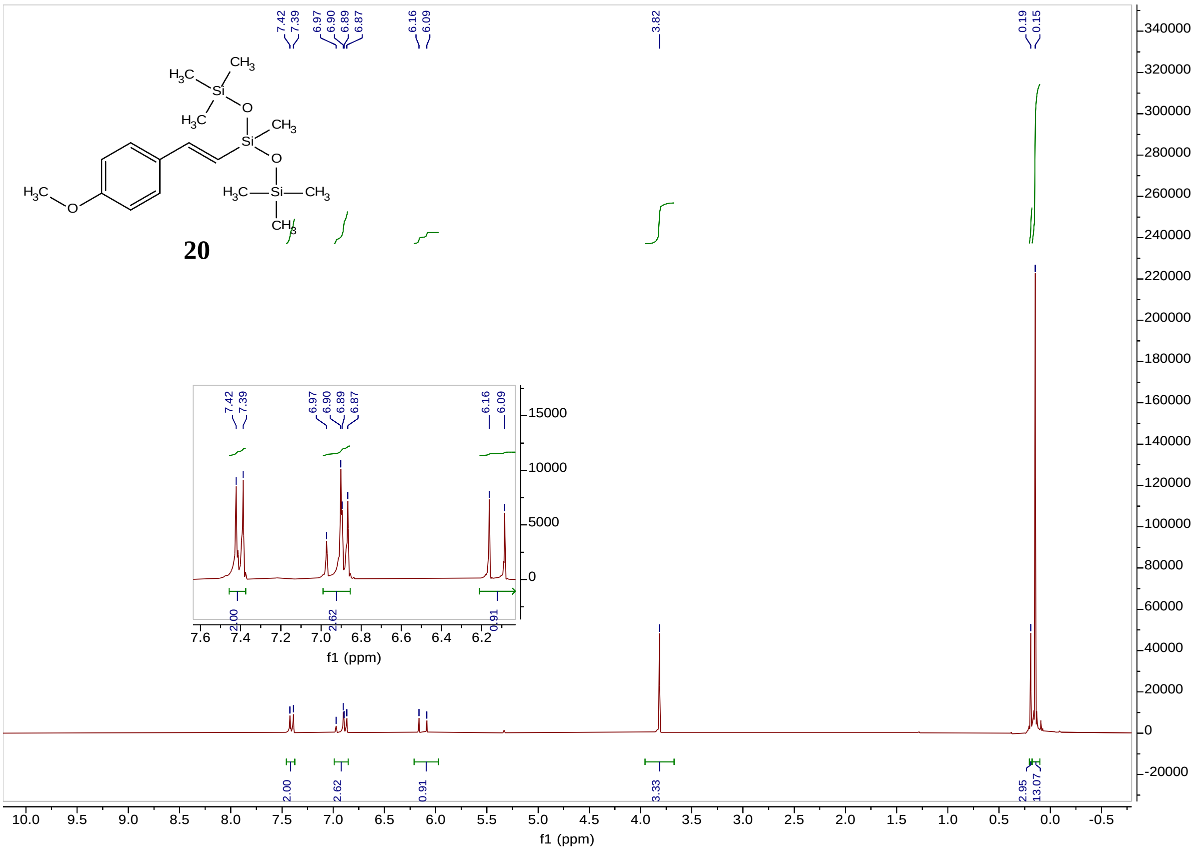
7.42
7.39
6.97
6.90
6.89
6.87
6.16
6.09
3.82
0.19
0.15
20
2.00
2.62
0.91
3.33
2.95
13.07
f1 (ppm)

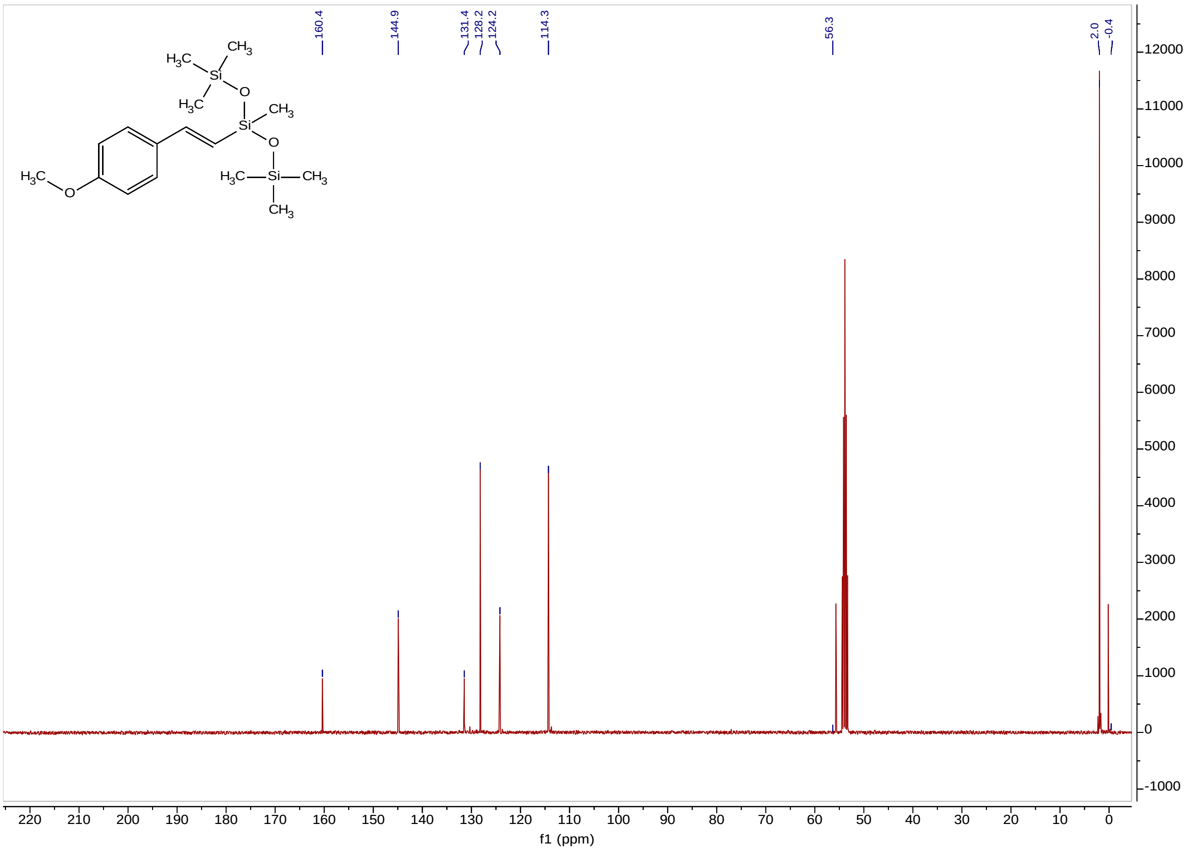
160.4
144.9
131.4
128.2
124.2
114.3
56.3
2.0
-0.4
f1 (ppm)

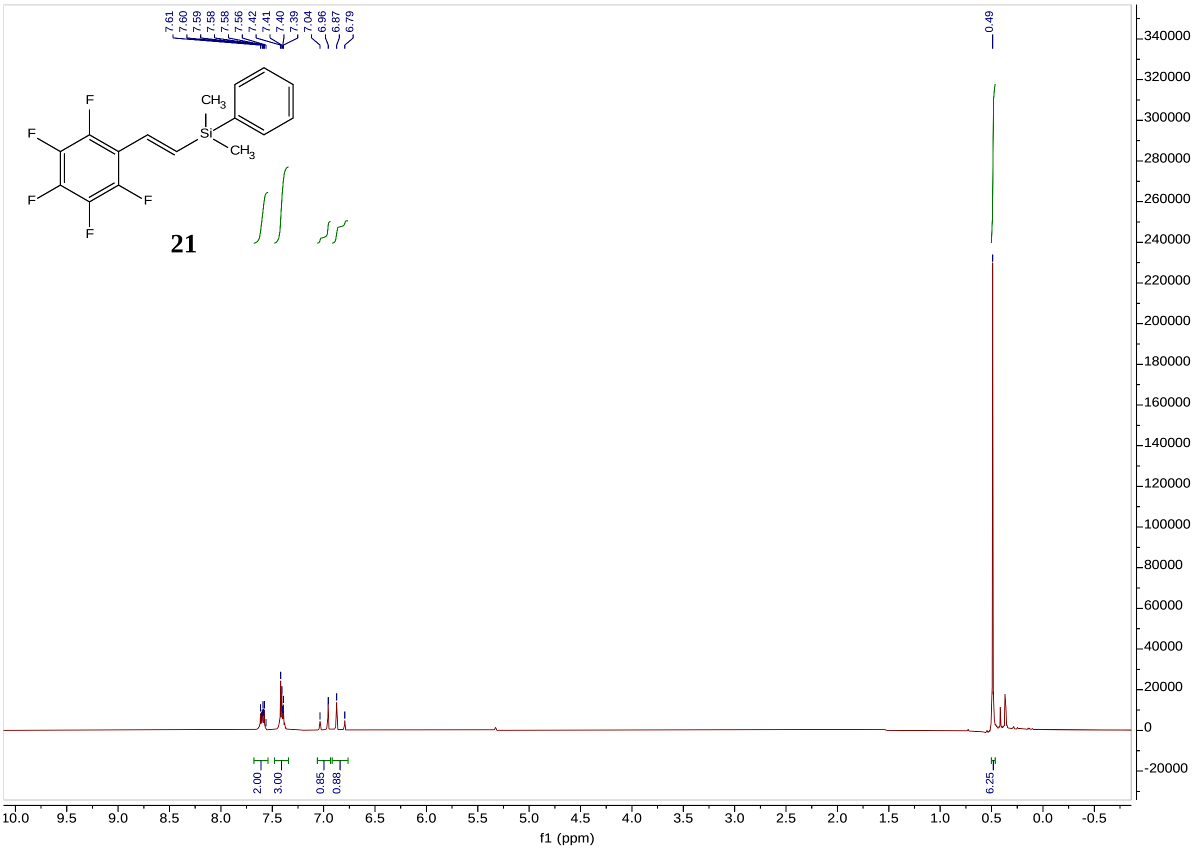
21
f1 (ppm)

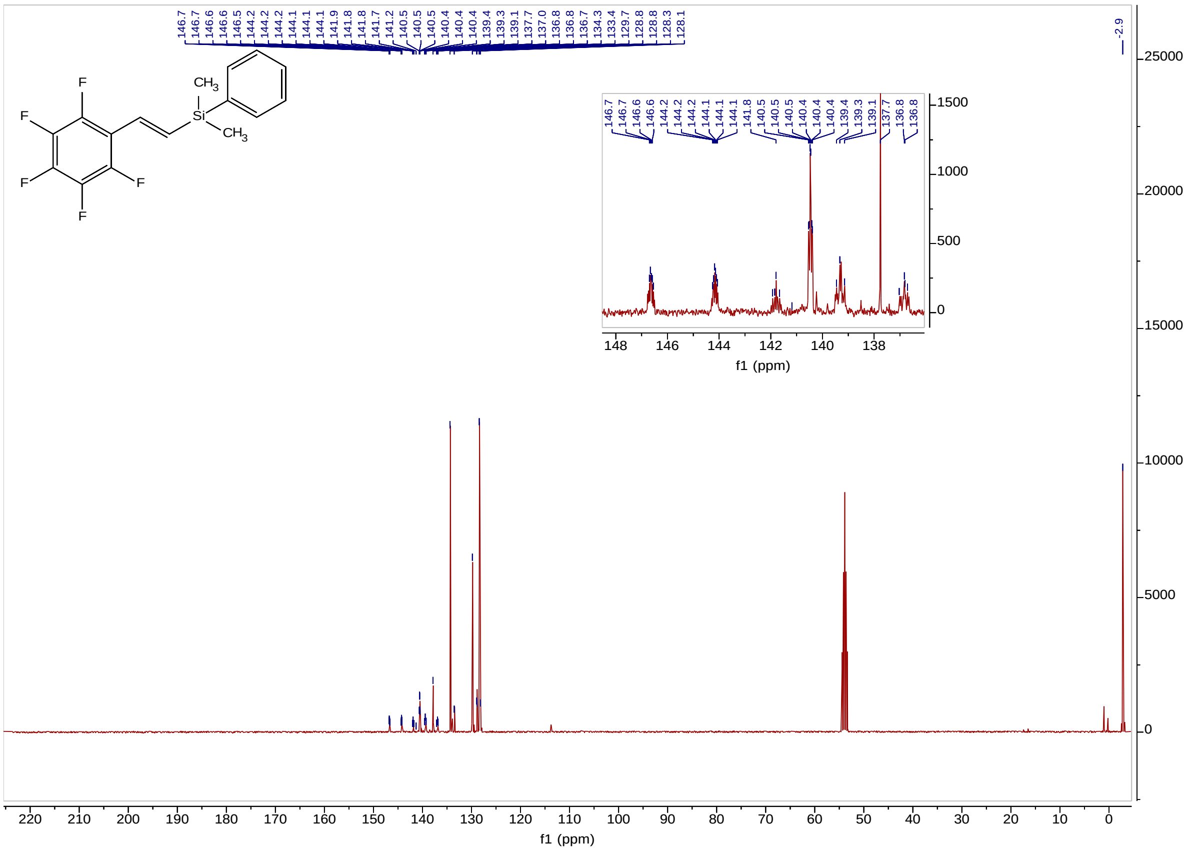
f1 (ppm)

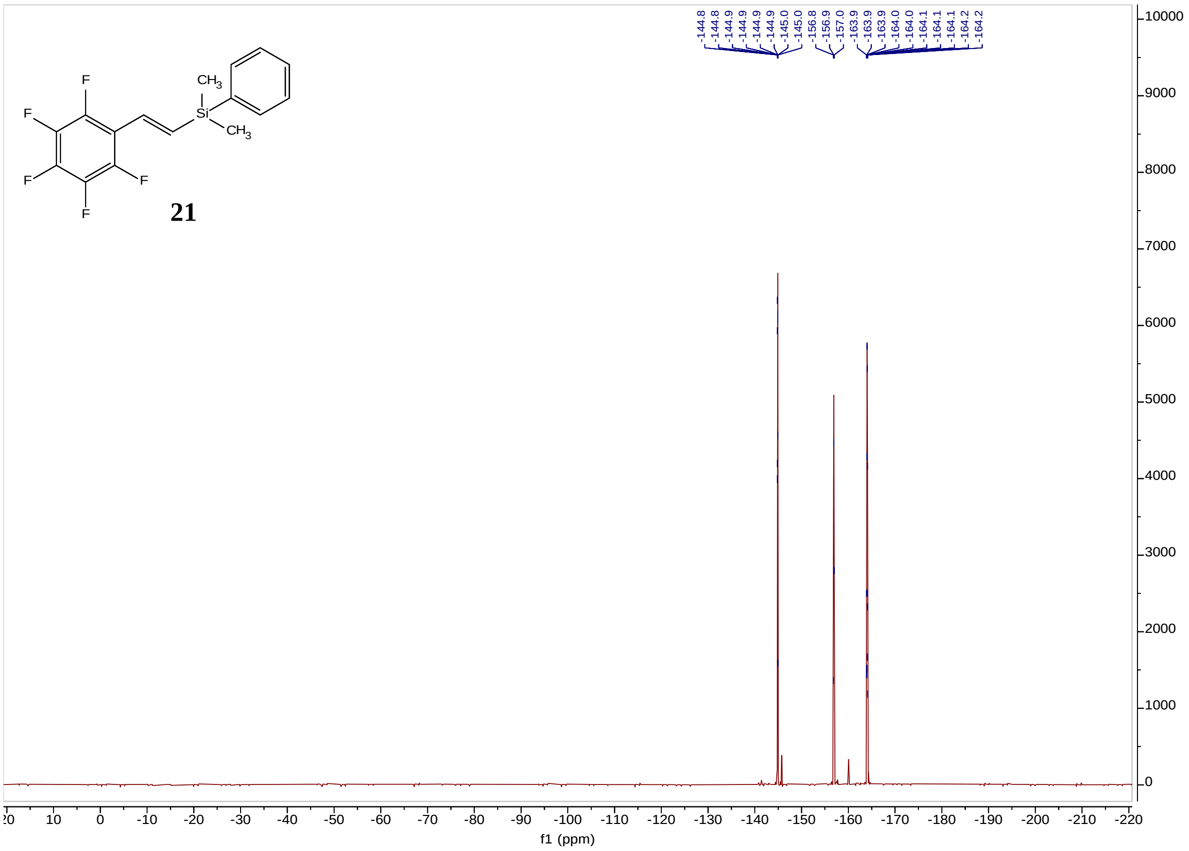

-144.8
-144.8
-144.9
-144.9
-144.9
-144.9
-145.0
-145.0
-156.8
-156.9
-157.0
-163.9
-163.9
-163.9
-164.0
-164.0
-164.1
-164.1
-164.1
-164.2
-164.2
F
F
F
F
F
CH3
Si
CH3
21
f1 (ppm)

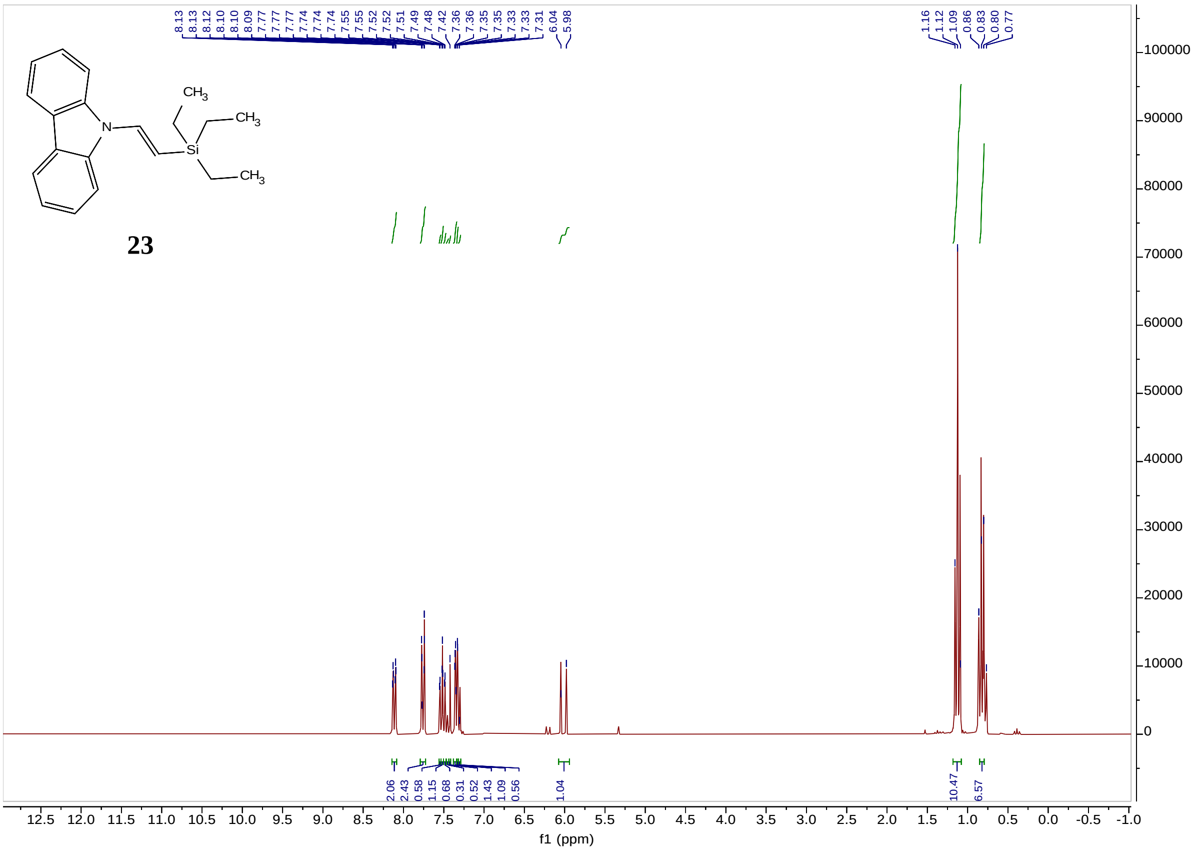
8.13
8.13
8.12
8.10
8.09
7.77
7.77
7.77
7.74
7.74
7.55
7.55
7.52
7.51
7.49
7.48
7.42
7.36
7.36
7.35
7.35
7.33
7.33
7.31
6.04
5.98
1.16
1.12
1.09
0.86
0.83
0.80
0.77
2.06
2.43
0.58
1.15
0.68
0.31
0.52
1.43
1.09
0.56
1.04
10.47
6.57
CH3
N
Si
23
f1 (ppm)

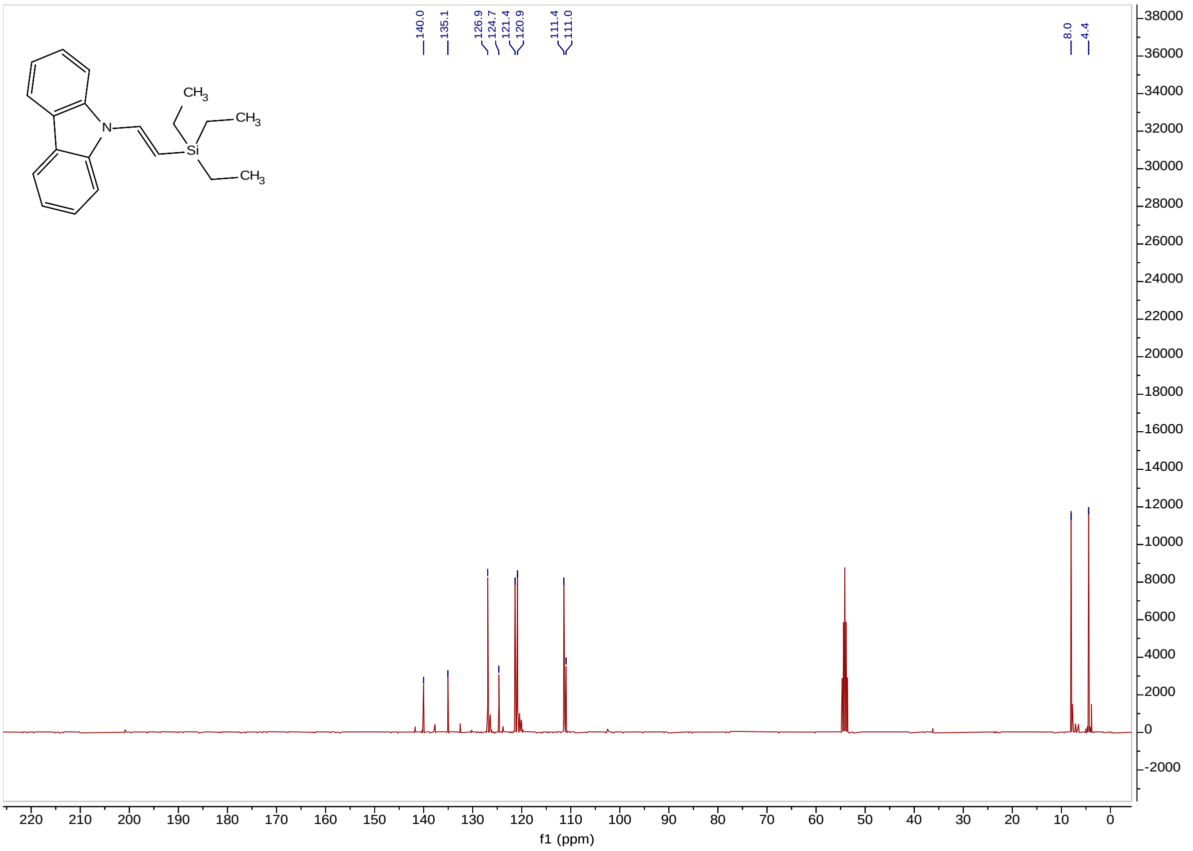
140.0
135.1
126.9
124.7
121.4
120.9
111.4
111.0
8.0
4.4
CH3
N
Si
f1 (ppm)

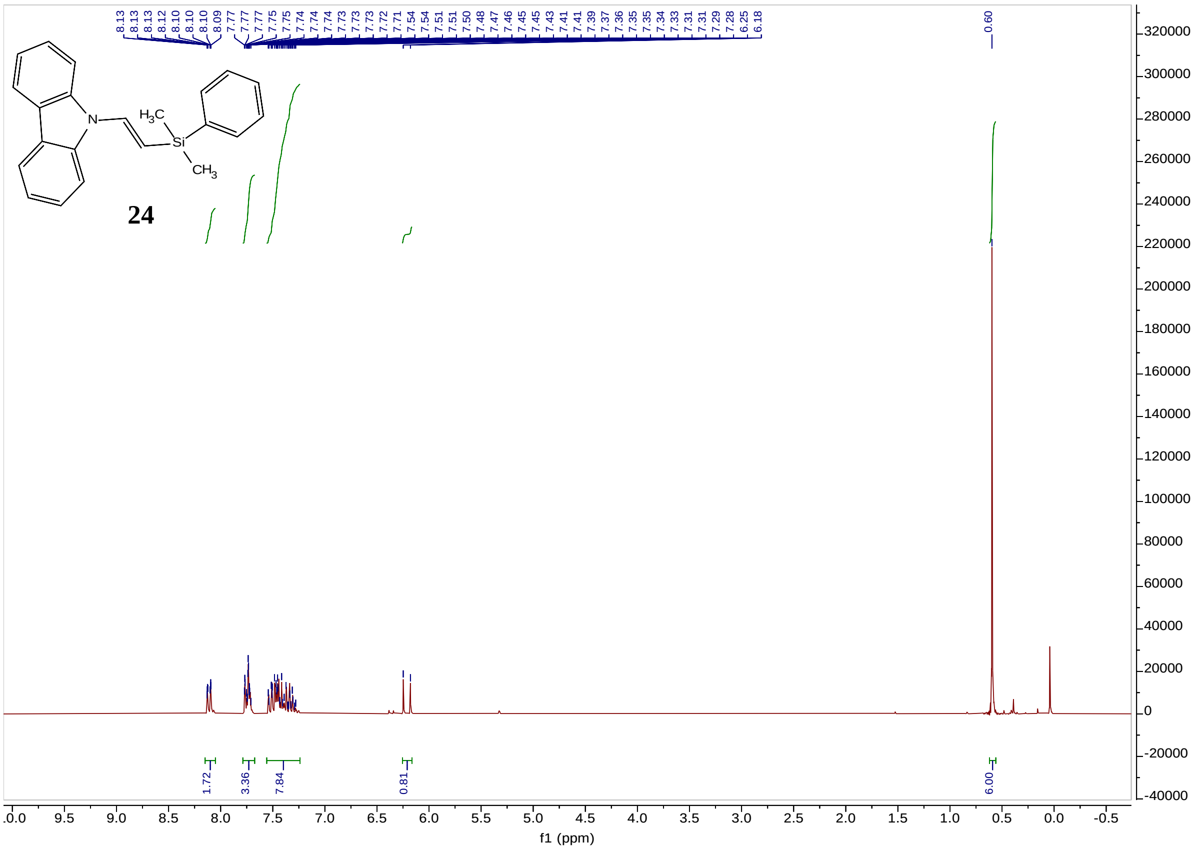
8.13
8.13
8.13
8.12
8.10
8.10
8.10
8.09
7.77
7.77
7.77
7.75
7.75
7.74
7.74
7.74
7.73
7.73
7.73
7.72
7.72
7.71
7.54
7.54
7.51
7.51
7.50
7.48
7.47
7.46
7.45
7.45
7.43
7.41
7.41
7.39
7.37
7.36
7.35
7.35
7.34
7.33
7.31
7.31
7.29
7.28
6.25
6.18
0.60
N
H3C
Si
CH3
24
1.72
3.36
7.84
0.81
6.00
f1 (ppm)

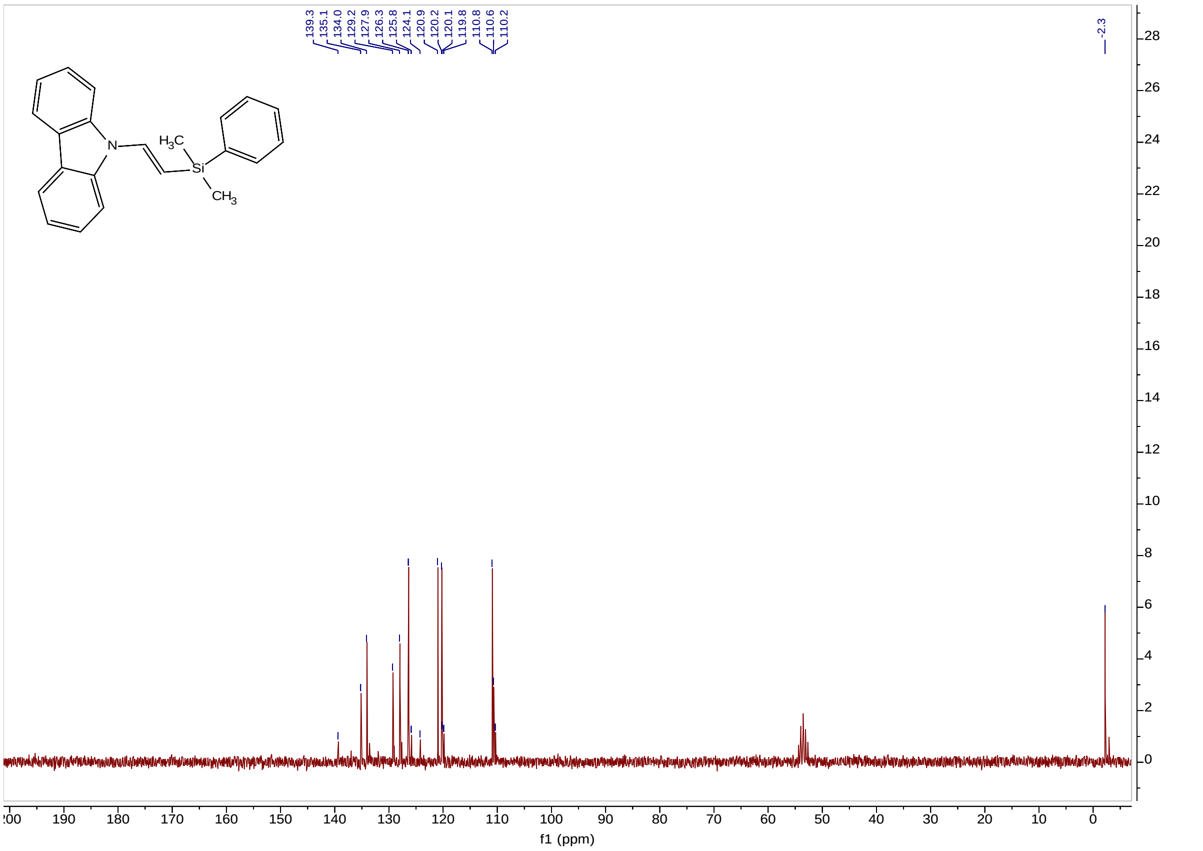
139.3
135.1
134.0
129.2
127.9
126.3
125.8
124.1
120.9
120.2
120.1
119.8
110.8
110.6
110.2
-2.3
N
H3C
Si
CH3
f1 (ppm)

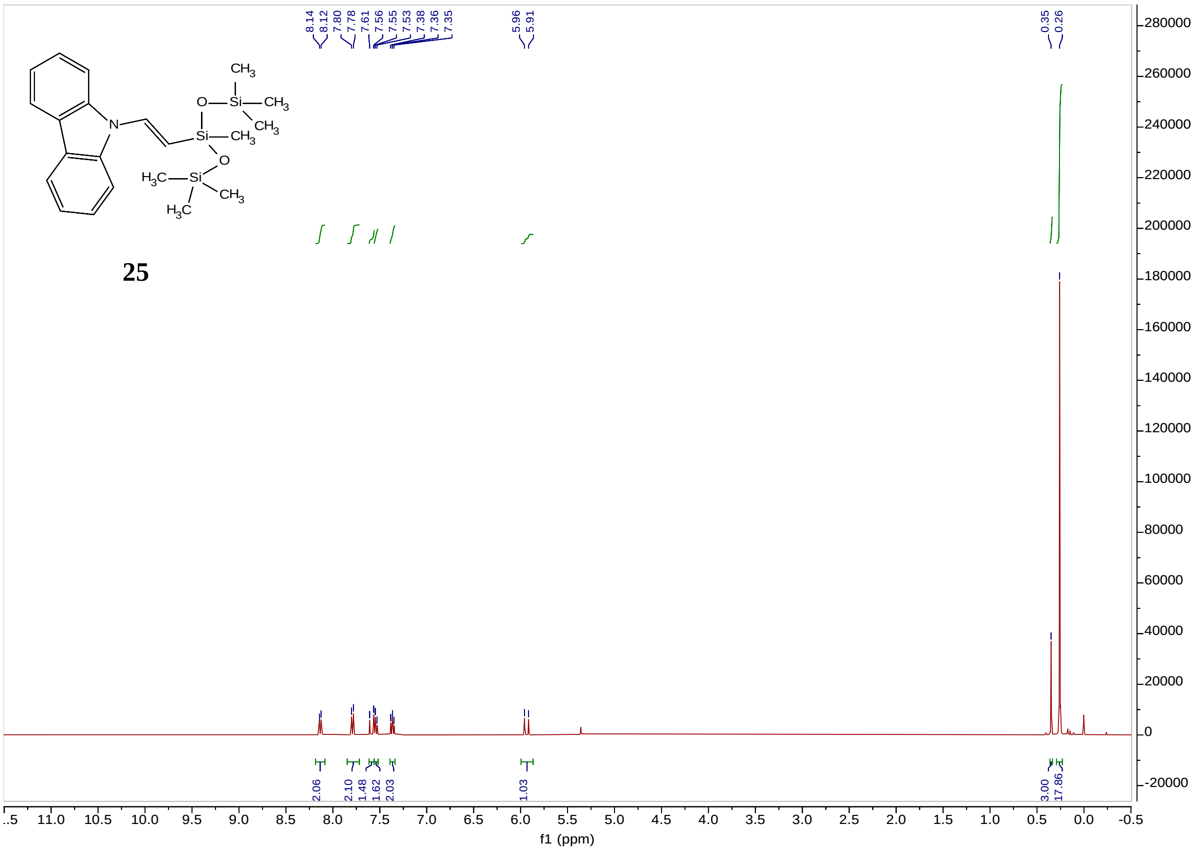
8.14
8.12
7.80
7.78
7.61
7.56
7.55
7.53
7.38
7.36
7.35
5.96
5.91
0.35
0.26
2.06
2.10
1.48
1.62
2.03
1.03
3.00
17.86
f1 (ppm)

25

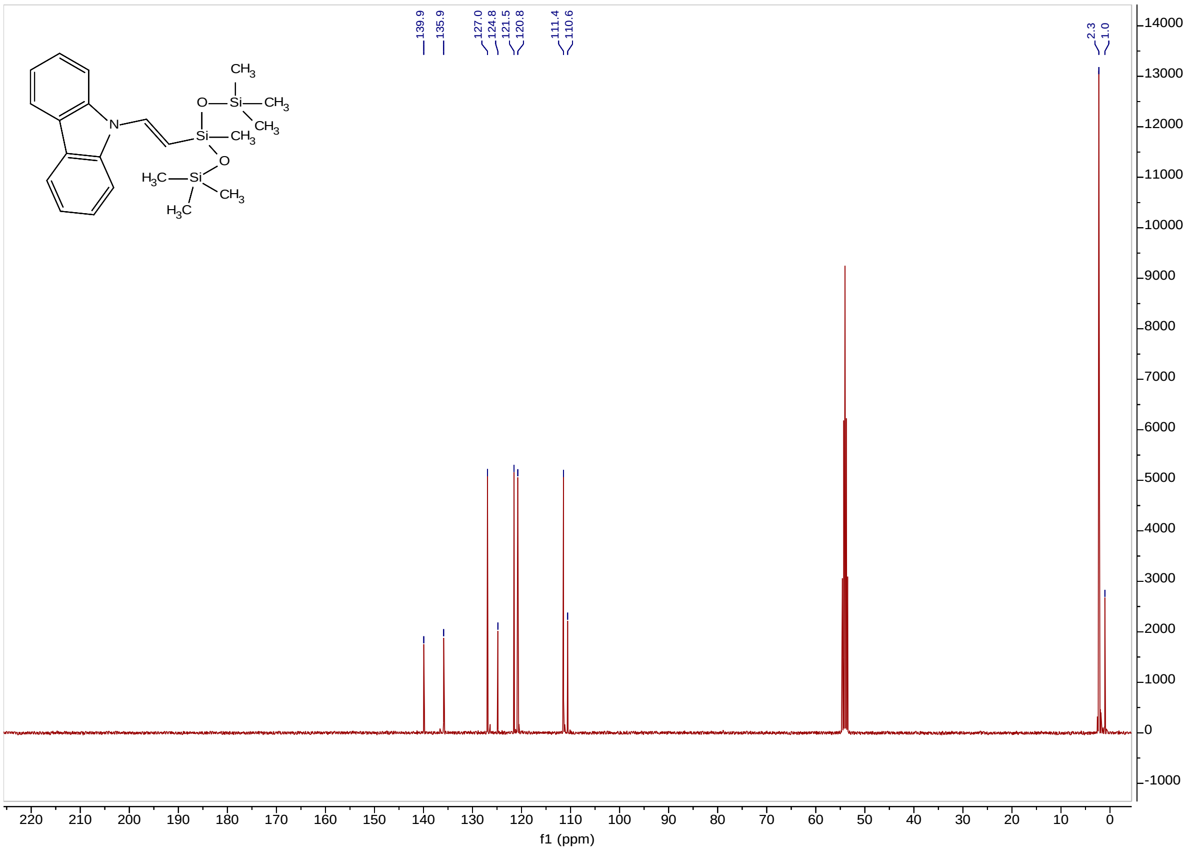
139.9
135.9
127.0
124.8
121.5
120.8
111.4
110.6
2.3
1.0
f1 (ppm)

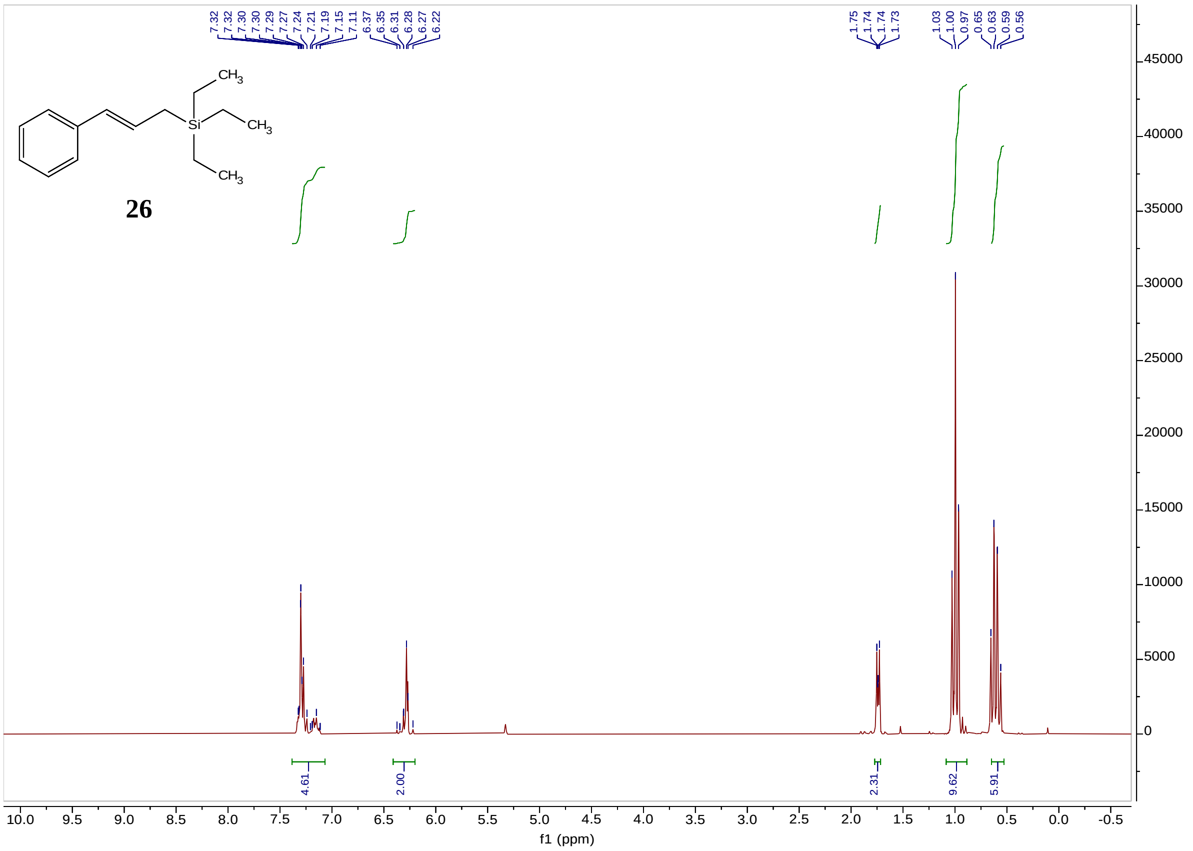
7.32
7.32
7.30
7.30
7.29
7.27
7.24
7.21
7.19
7.15
7.11
6.37
6.35
6.31
6.28
6.27
6.22
1.75
1.74
1.74
1.73
1.03
1.00
0.97
0.65
0.63
0.59
0.56
CH3
Si
CH3
CH3
26
4.61
2.00
2.31
9.62
5.91
f1 (ppm)

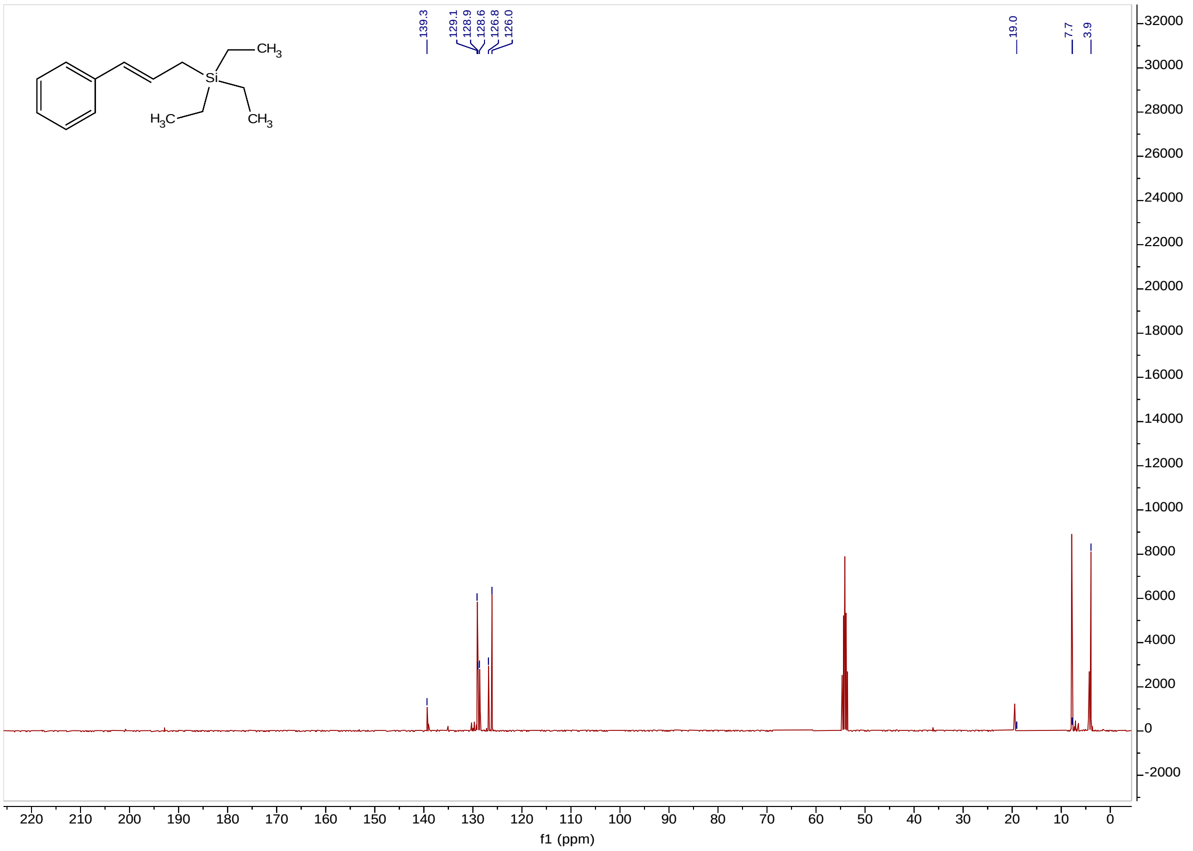
139.3
129.1
128.9
128.6
126.8
126.0
19.0
7.7
3.9
CH3
Si
H3C
CH3
f1 (ppm)

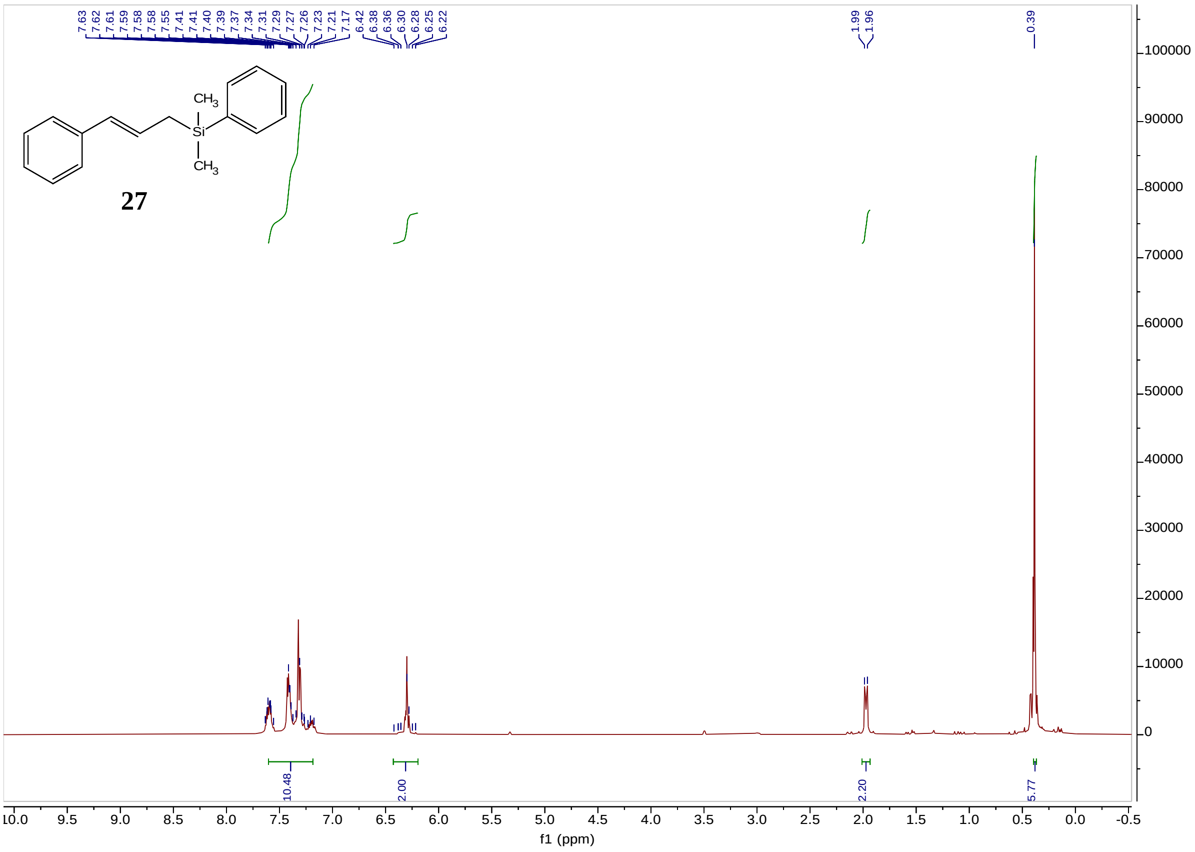
7.63
7.62
7.61
7.59
7.58
7.58
7.55
7.41
7.41
7.40
7.39
7.37
7.34
7.31
7.29
7.27
7.26
7.23
7.21
7.17
6.42
6.38
6.36
6.30
6.28
6.25
6.22
1.99
1.96
0.39
CH3
Si
CH3
27
10.48
2.00
2.20
5.77
f1 (ppm)

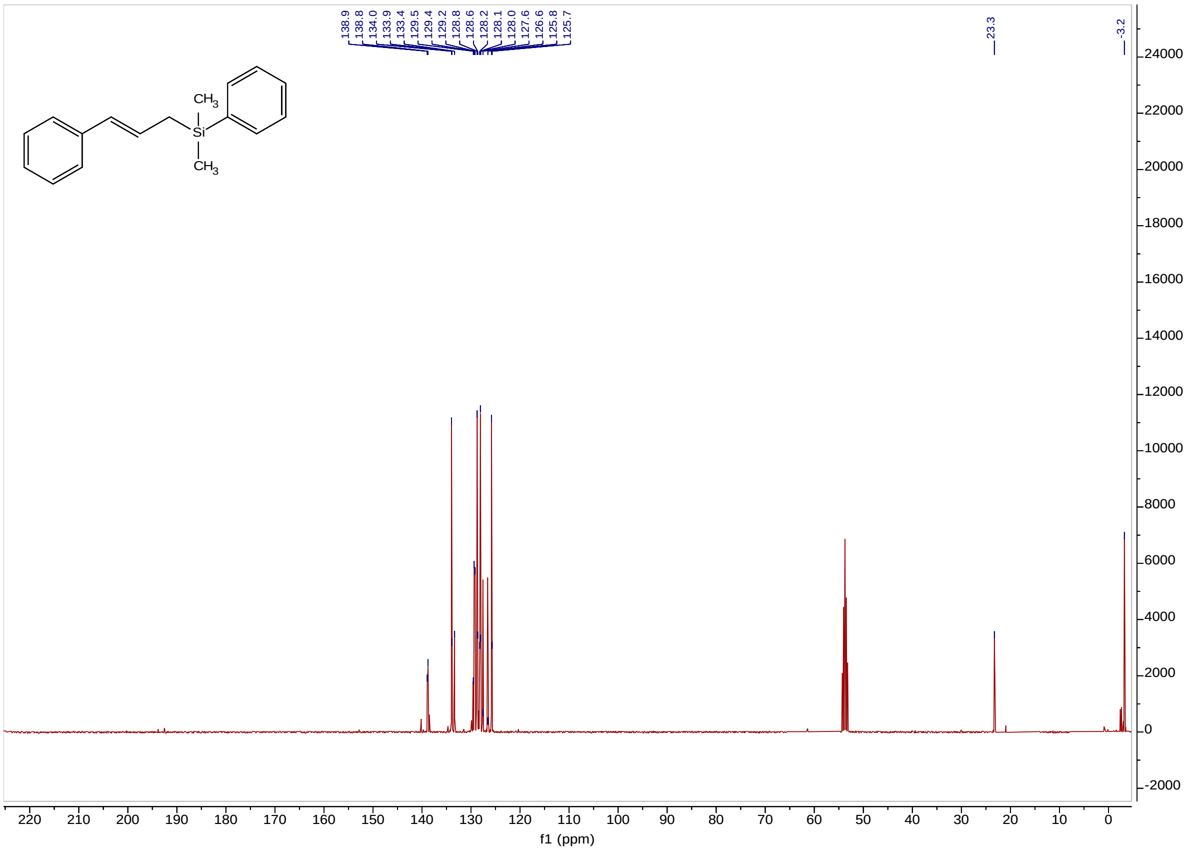
138.9
138.8
134.0
133.9
133.4
129.5
129.4
129.2
128.8
128.6
128.2
128.1
128.0
127.6
126.6
125.8
125.7
23.3
-3.2
CH3
Si
CH3
f1 (ppm)

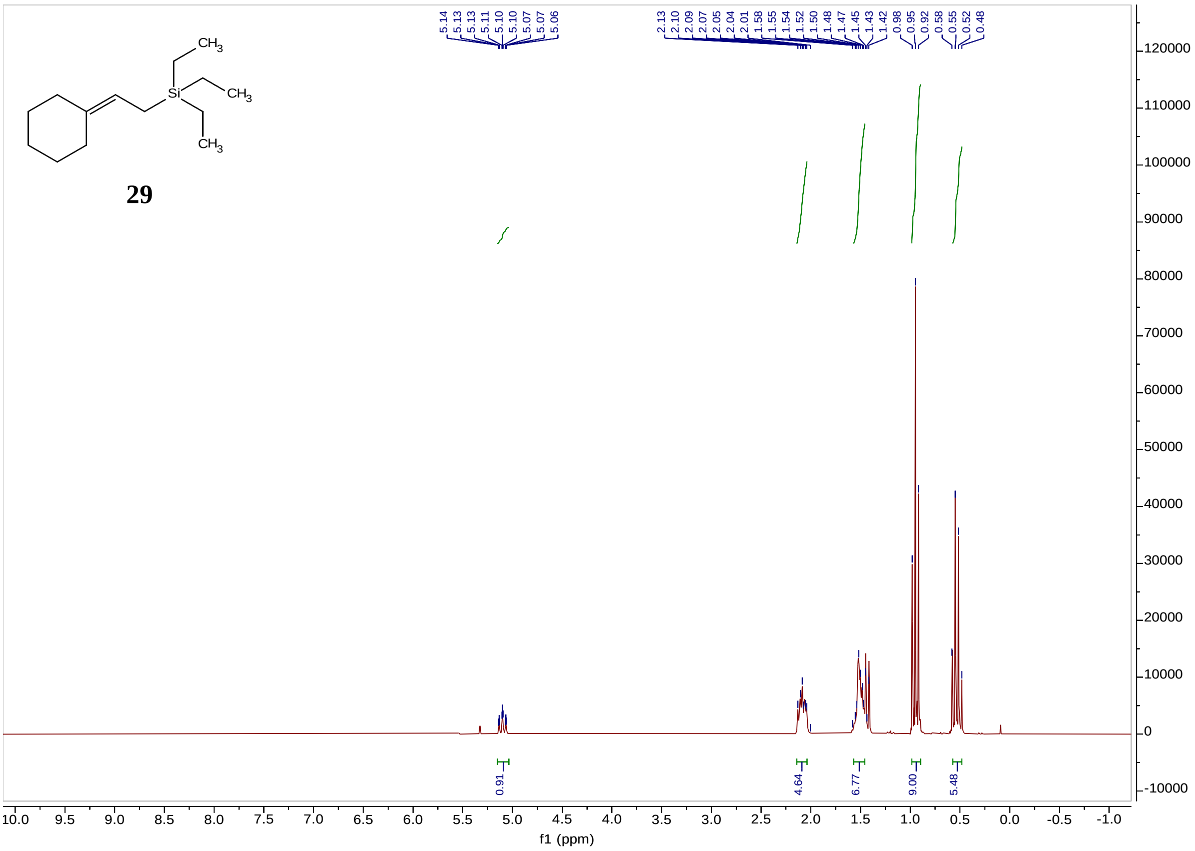
CH3
Si
CH3
CH3
5.14
5.13
5.13
5.11
5.10
5.10
5.07
5.07
5.06
2.13
2.10
2.09
2.07
2.05
2.04
2.01
1.58
1.55
1.54
1.52
1.50
1.48
1.47
1.45
1.43
1.42
0.98
0.95
0.92
0.58
0.55
0.52
0.48
0.91
4.64
6.77
9.00
5.48
f1 (ppm)

29

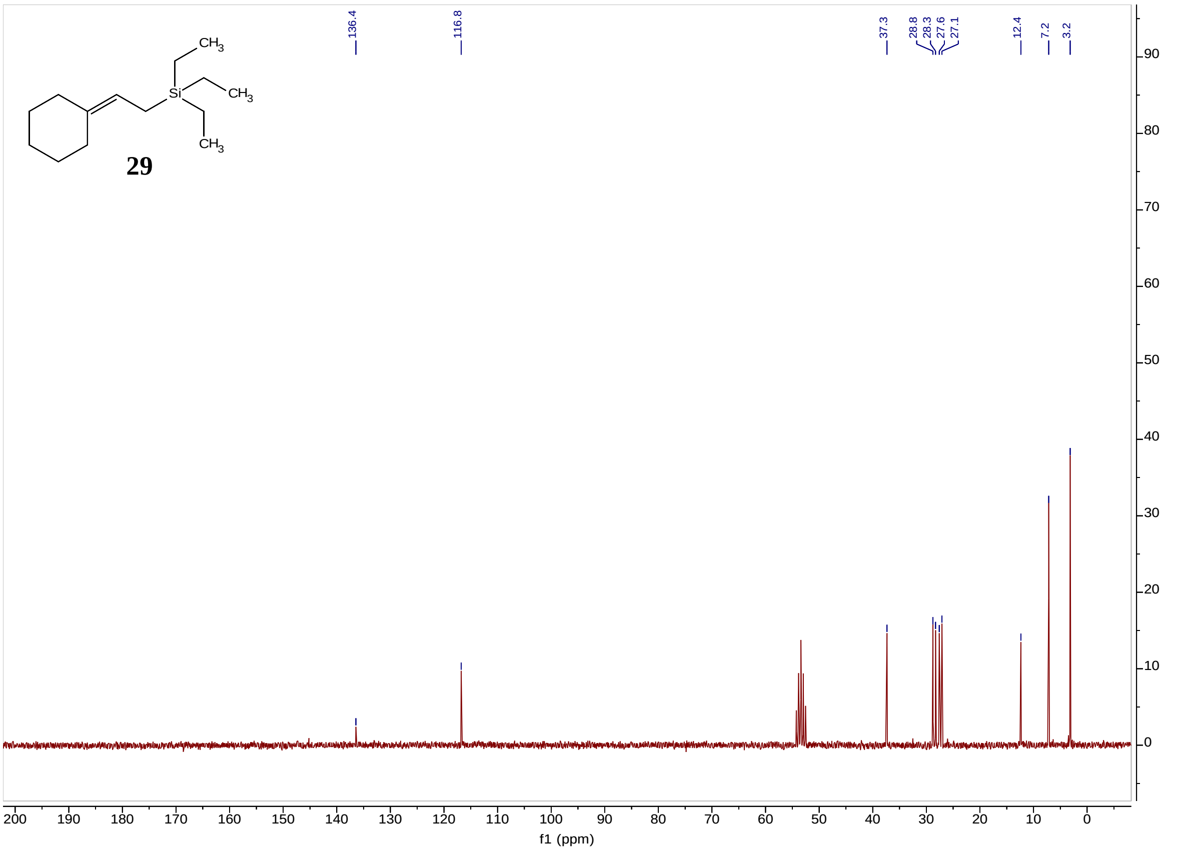
CH3
Si
CH3
CH3
136.4
116.8
37.3
28.8
28.3
27.6
27.1
12.4
7.2
3.2
f1 (ppm)

29

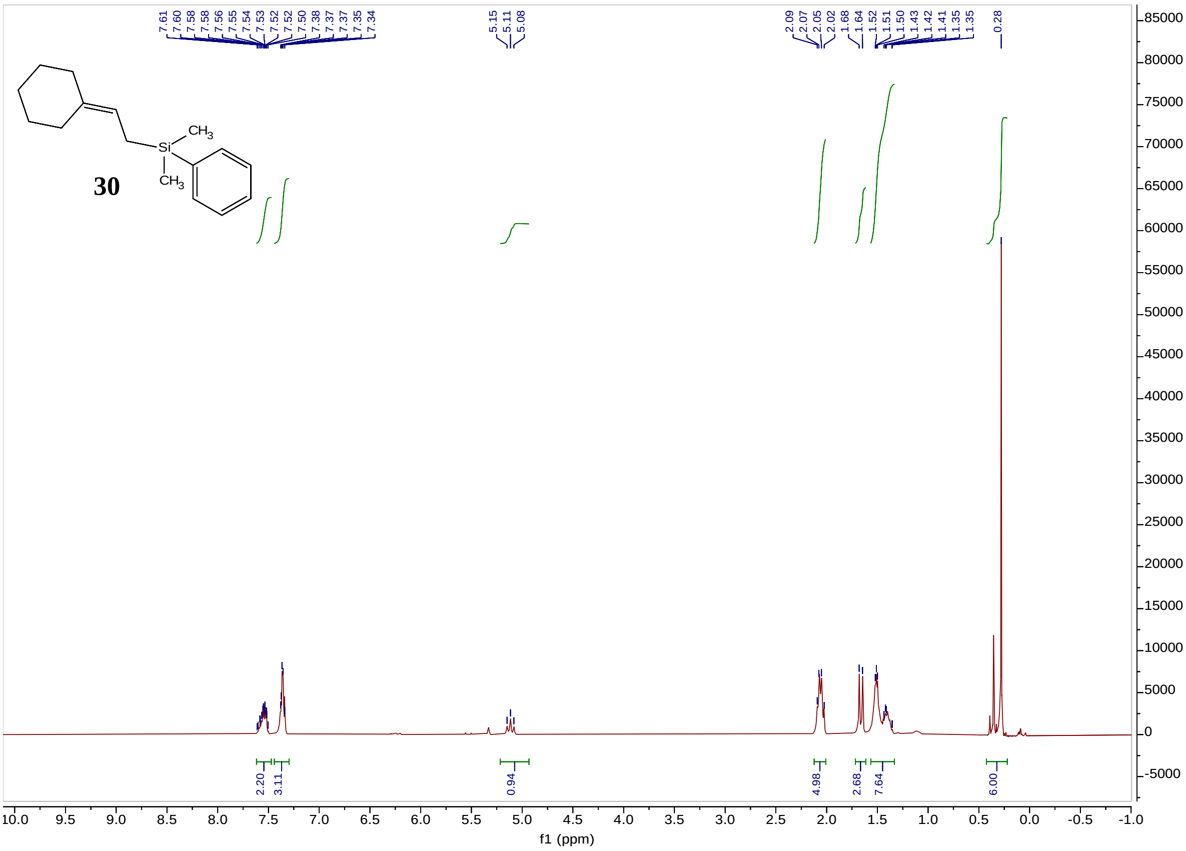
7.61 7.60 7.58 7.58 7.56 7.55 7.54 7.53 7.52 7.52 7.50 7.38 7.37 7.37 7.35 7.34
5.15 5.11 5.08
2.09 2.07 2.05 2.02 1.68 1.64 1.52 1.51 1.50 1.43 1.42 1.41 1.35 1.35
0.28
CH3
Si
CH3
30
2.20 3.11 0.94 4.98 2.68 7.64 6.00
f1 (ppm)

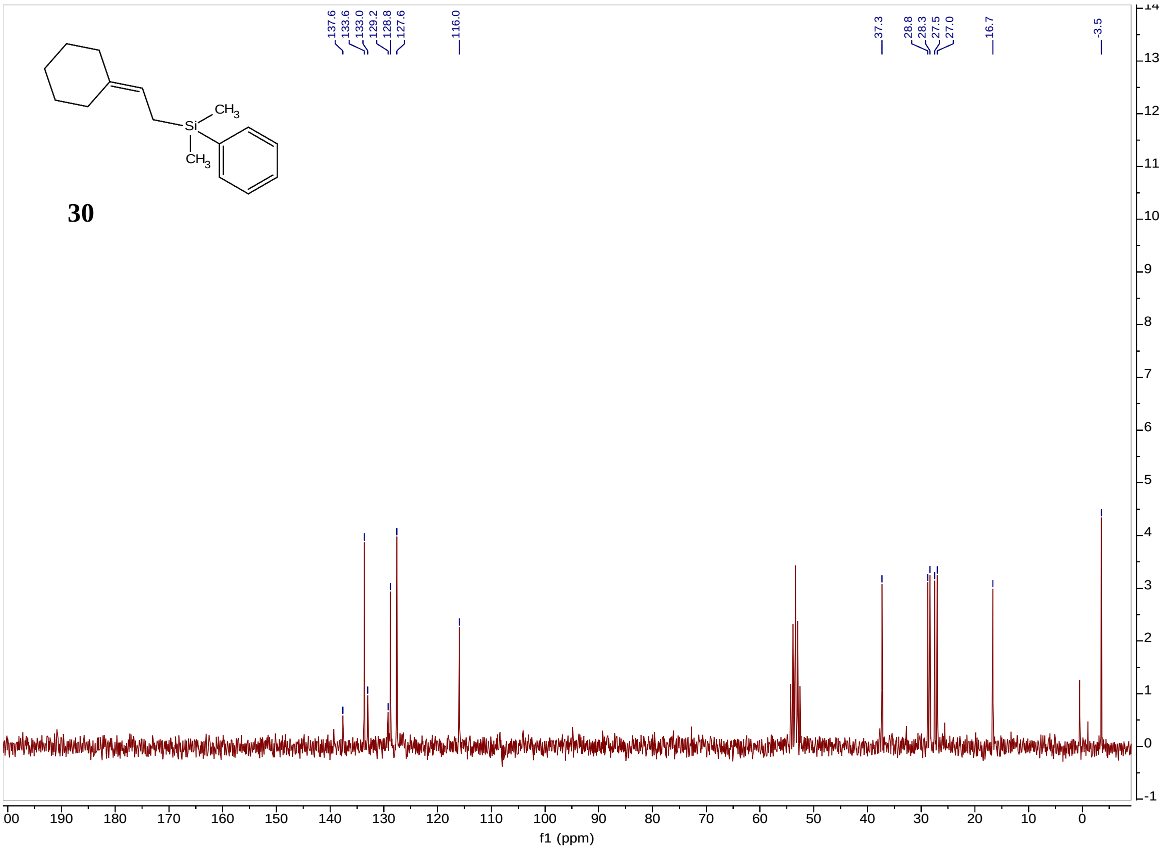
137.6 133.6 133.0 129.2 128.8 127.6
116.0
37.3
28.8 28.3 27.5 27.0
16.7
-3.5
CH3
Si
CH3
30
f1 (ppm)

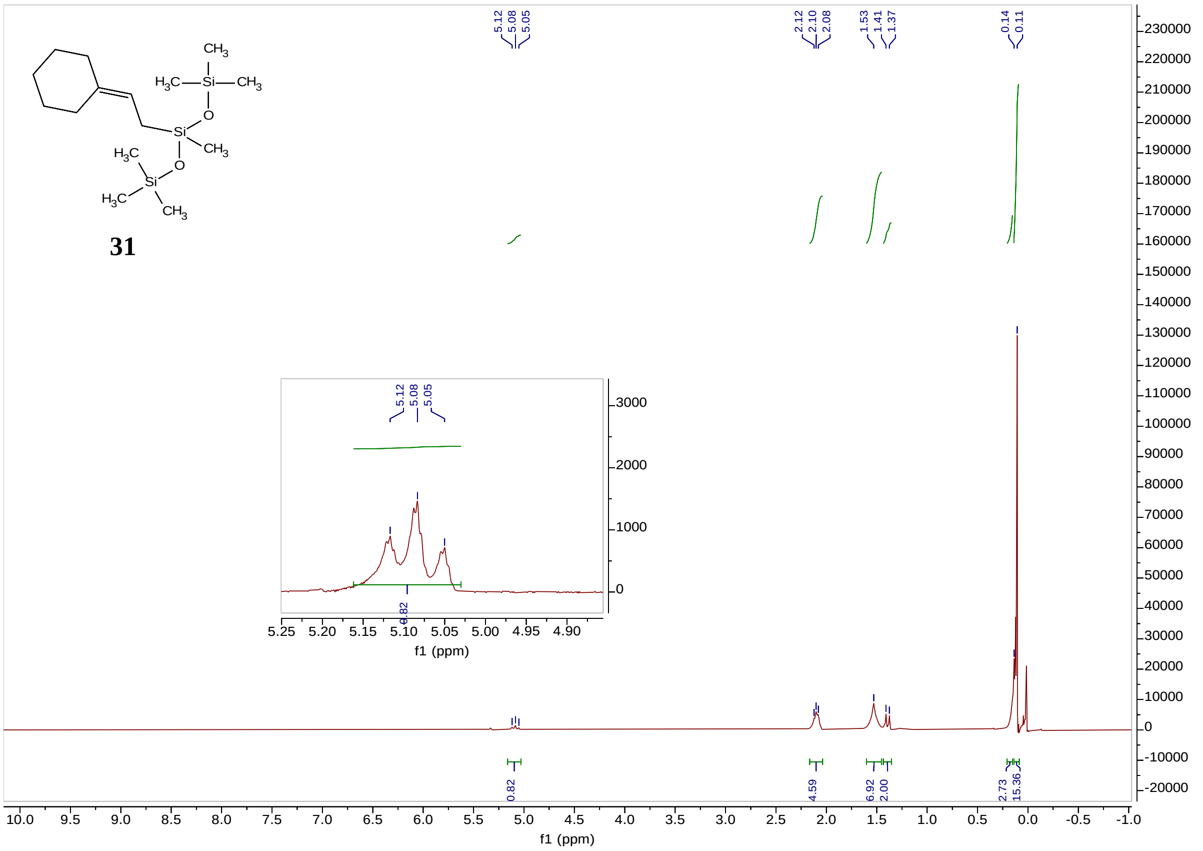
31
5.12
5.08
5.05
2.12
2.10
2.08
1.53
1.41
1.37
0.14
0.11
0.82
4.59
6.92
2.00
2.73
15.36
f1 (ppm)

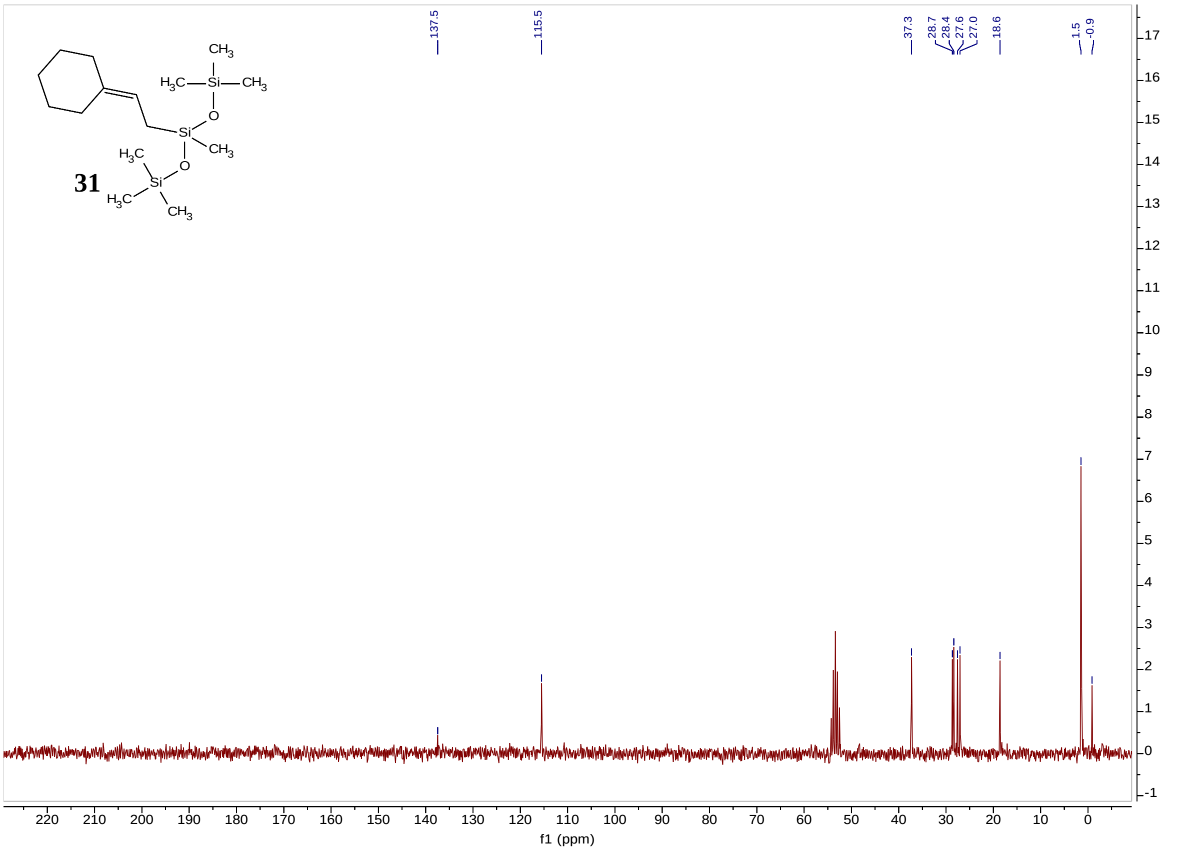
31
137.5
115.5
37.3
28.7
28.4
27.6
27.0
18.6
1.5
-0.9
f1 (ppm)

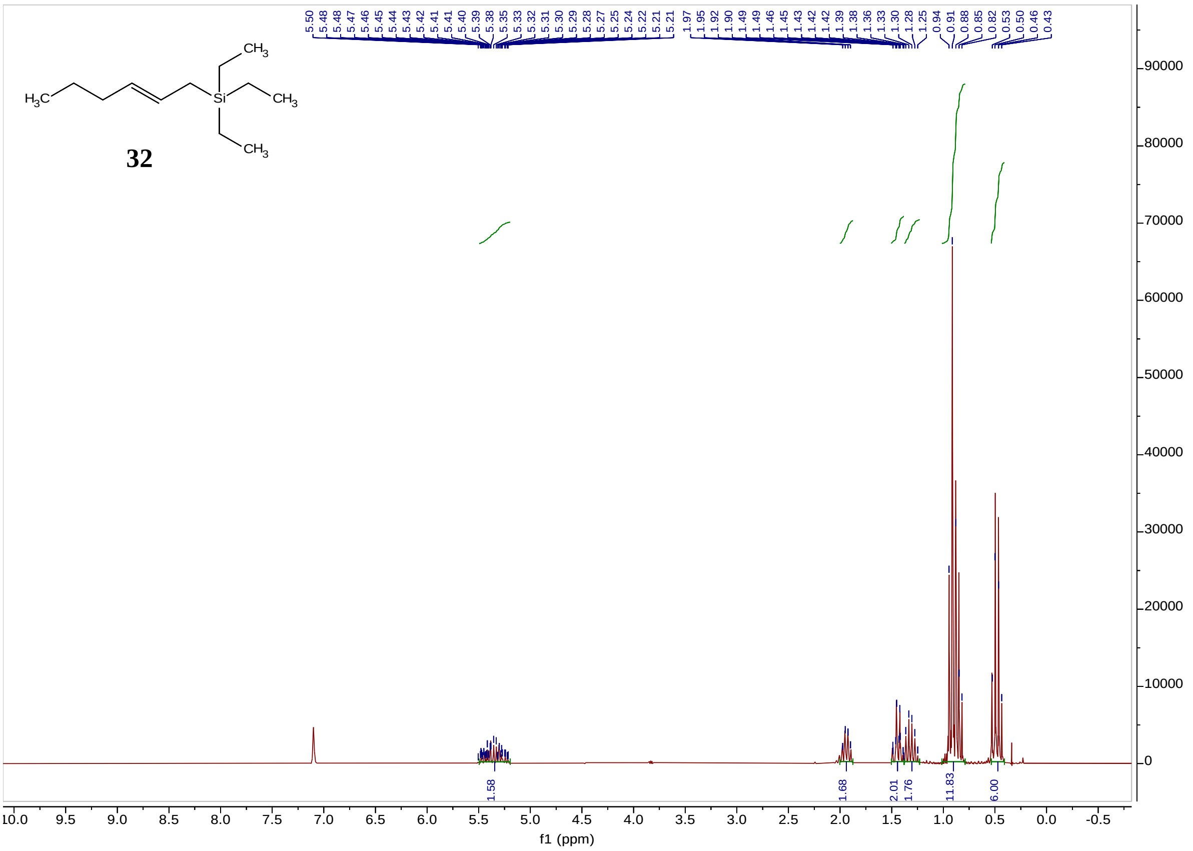

32
5.50 5.48 5.48 5.47 5.46 5.45 5.44 5.43 5.42 5.41 5.41 5.40 5.39 5.38 5.35 5.33 5.32 5.31 5.30 5.29 5.28 5.27 5.25 5.24 5.22 5.21 5.21
1.97 1.95 1.92 1.90 1.49 1.49 1.46 1.45 1.43 1.42 1.42 1.39 1.38 1.36 1.33 1.30 1.28 1.25 0.94 0.91 0.88 0.85 0.82 0.53 0.50 0.46 0.43
1.58
1.68
2.01
1.76
11.83
6.00
f1 (ppm)

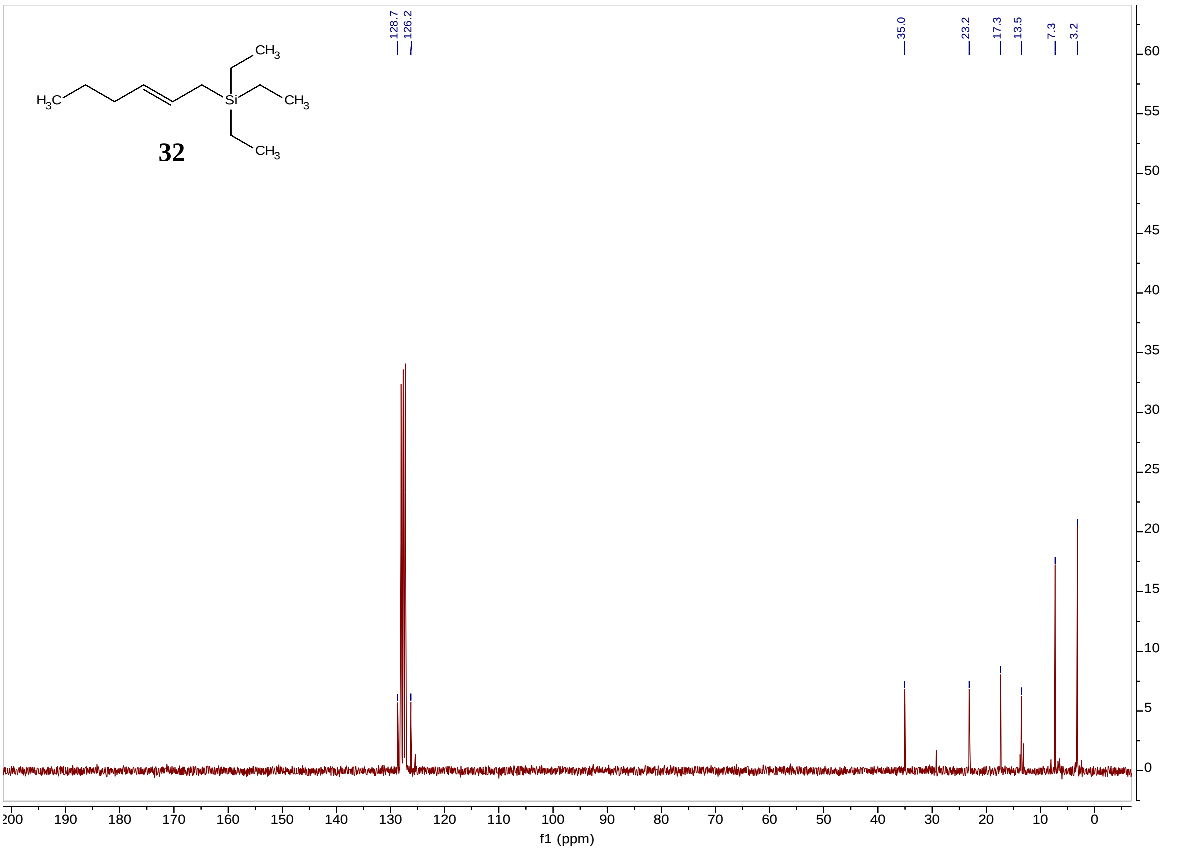

32
128.7
126.2
35.0
23.2
17.3
13.5
7.3
3.2
f1 (ppm)

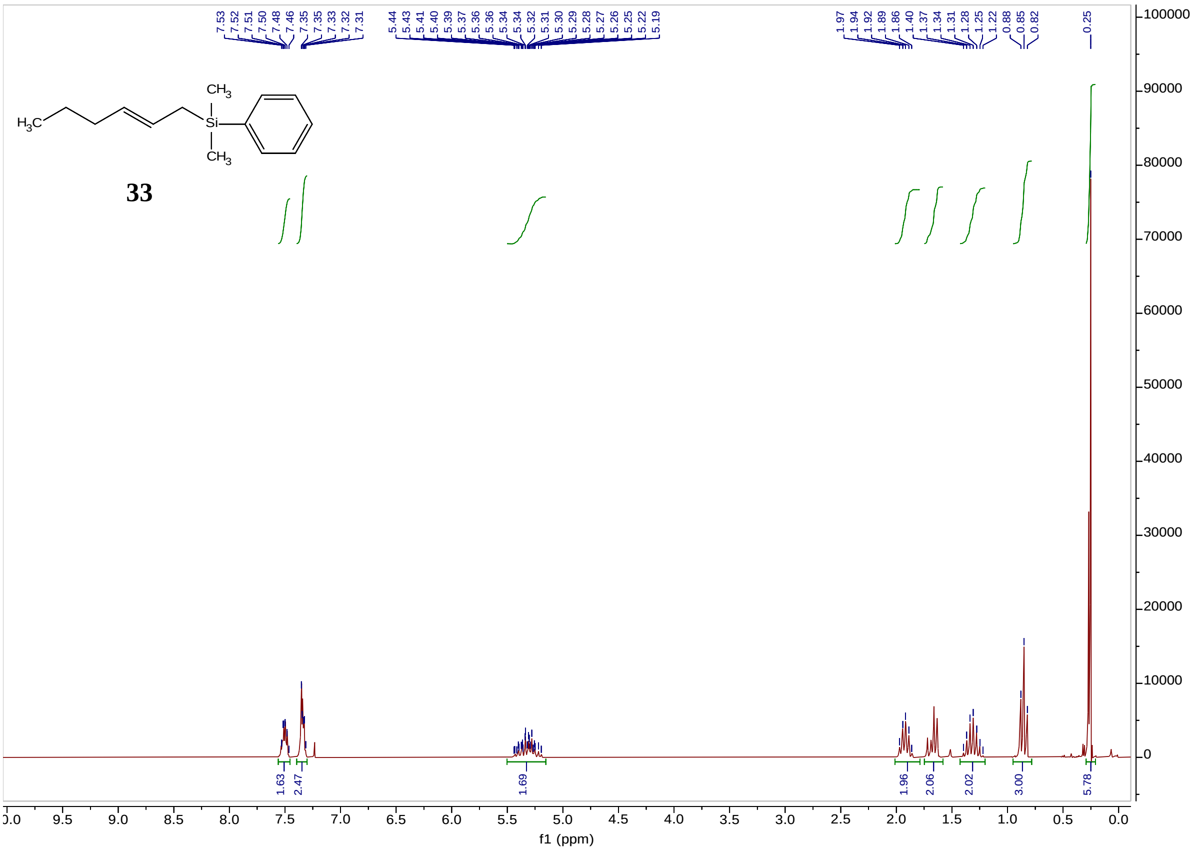
7.53
7.52
7.51
7.50
7.48
7.46
7.35
7.35
7.33
7.32
7.31
5.44
5.43
5.41
5.40
5.39
5.37
5.36
5.36
5.34
5.34
5.32
5.31
5.30
5.29
5.28
5.27
5.26
5.25
5.22
5.19
1.97
1.94
1.92
1.89
1.86
1.40
1.37
1.34
1.31
1.28
1.25
1.22
0.88
0.85
0.82
0.25
33
1.63
2.47
1.69
1.96
2.06
2.02
3.00
5.78
f1 (ppm)

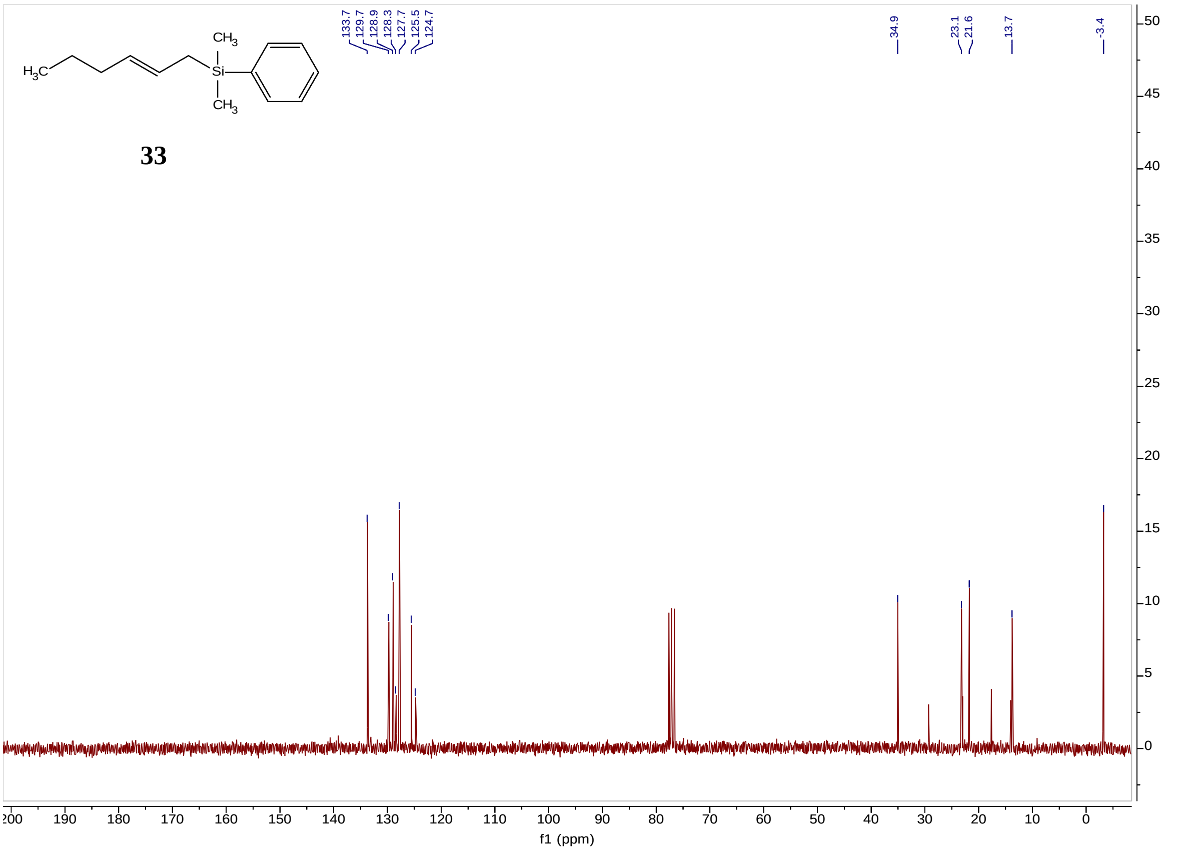
133.7
129.7
128.9
128.3
127.7
125.5
124.7
34.9
23.1
21.6
13.7
-3.4
33
f1 (ppm)
